# Pursuing Heteroleptic Ligand Design Principles for Photoactive Fe Complexes with Ultrafast X‑ray Emission and Variable-Temperature Optical Spectroscopies

**DOI:** 10.1021/jacs.6c09850

**Published:** 2026-07-28

**Authors:** Hyeongtaek Lim, Karl C. Nielsen, Natalia E. Powers-Riggs, Sumana L. Raj, Roberto Alonso-Mori, Leland B. Gee, Tim B. van Driel, Patrick L. Kramer, Matthieu Chollet, Amy A. Cordones, James K. McCusker, Kelly J. Gaffney

**Affiliations:** † Stanford PULSE Institute, SLAC National Accelerator Laboratory, 497525 Stanford University, Menlo Park, California 94025, United States; ‡ Department of Chemistry, 3078 Michigan State University, East Lansing, Michigan 48824, United States; § Linac Coherent Light Source, SLAC National Accelerator Laboratory, 17220 Stanford University, Menlo Park, California 94025, United States

## Abstract

Understanding the key parameters that govern the photophysical and photochemical properties of transition metal complexes is essential for the development of efficient photosensitizers for photocatalytic applications. Achieving this objective necessitates clear and detailed investigations of their electronic excited states, for which time-resolved metal Kβ X-ray emission spectroscopy (XES) has proven highly effective. Here, we present a time-resolved Fe Kβ XES study of a heteroleptic Fe­(II) polypyridyl carbene complex, [Fe­(phen)_2_(C_4_H_10_N_4_)]^2+^ (**1**; phen = 1,10-phenanthroline), utilizing both the valence-to-core and Kβ mainline spectral regions, complemented by variable-temperature transient optical absorption (VT-TA) spectroscopy. Detailed analysis of the time-resolved Kβ XES data, supported by density functional theory (DFT) calculations and an Eyring analysis of the VT-TA data, reveals parallel excited state relaxation dynamics that support an assignment of the long-lived excited state to a triplet metal-centered state. Placing these results in the context of prior studies of heteroleptic Fe­(II) polypyridyl cyanide complexes motivated a series of DFT calculations to investigate the effects of ligand structural flexibility and arrangement. These calculations reinforce the experimentally derived conclusion that constraining structural flexibility with multidentate ligands significantly impacts the excited state relaxation dynamics. Furthermore, our study emphasizes that the arrangement of strong field ligands in heteroleptic complexes substantially affects the energy of Jahn–Teller active triplet metal-centered states in low-spin d^6^ metal complexes. Together, these findings provide synthetic design principles for extending metal-to-ligand charge transfer excited state lifetimes of heteroleptic Fe complexes.

## Graphical abstract

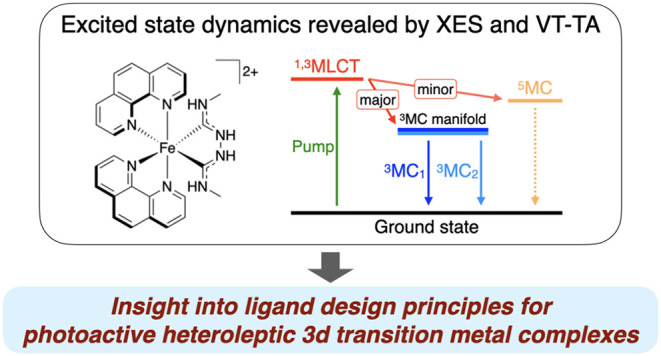

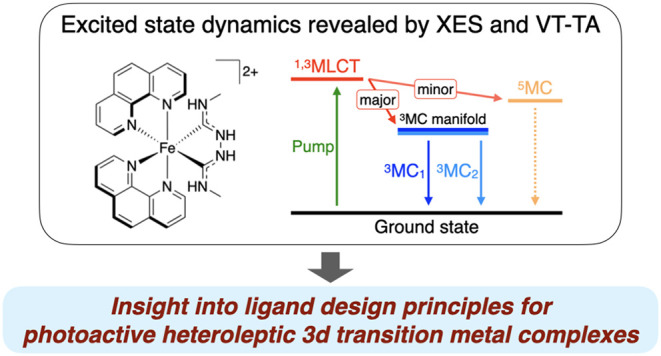

## 1 Introduction

Charge transfer (CT) excited states play crucial roles in the photocatalytic and photosensitizing applications of transition metal complexes.^1–5^ In these applications, a long CT excited state lifetime is an important performance metric. A long-standing goal has been to replace costly and rare second- and third-row transition metals, such as Ru and Ir, with earth-abundant first-row transition metals like Fe.^6–12^ However, first-row transition metal analogues of heavier second- and third-row transition metal complexes generally exhibit much shorter CT excited state lifetimes due to the presence of low-lying metal-centered (MC) excited states, often referred to as ligand-field excited states, that efficiently quench the CT excited states.^13^

One strategy to extend CT excited state lifetimes in first-row transition metal complexes is to employ strongly σ-donating ligands, such as carbenes, to increase the ligand field strength and thereby destabilize MC excited states.^14–18^ We recently adopted a modular approach to synthesize a heteroleptic low-spin pseudo-octahedral Fe­(II) polypyridyl carbene complex, [Fe­(phen)_2_(C_4_H_10_N_4_)]^2+^ (**1**; phen = 1,10-phenanthroline), which is shown in Figure 1.^19^ The design principle underpinning the preparation of complex **1** was to use two chelating polypyridyl phen ligands for light harvesting via metal-to-ligand charge transfer (MLCT)-type absorptions, while the bidentate carbene ligand would serve to modulate the ligand field strength to destabilize the MC excited states relative to the MLCT manifold. Time-resolved optical absorption measurements at room temperature revealed excited state dynamics of complex **1** qualitatively and quantitatively distinct from those of [Fe­(phen)_3_]^2+^ and Fe­(phen)_2_(CN)_2_, two complexes that served as benchmarks for comparison and display dynamics typical of Fe­(II) polypyridyl complexes.^19^ While it was reasonably clear that the effective ligand field strength of complex **1** was sufficient to invert the relative energies of the ^3^T_1_ and ^5^T_2_ states, thereby making the ^3^T_1_ state the lowest-energy MC excited state, the time-resolved optical absorption data did not definitively resolve whether the triplet MLCT (^3^MLCT) state, the ^3^T_1_ state, or a quasi-equilibrium between the two drove the ∼7 ps ground state recovery dynamics.^19^ This is not an isolated occurrence: UV–vis pump–probe spectroscopy often lacks clear or unique signatures of MC excited states, making their potential role in MLCT relaxation difficult to characterize.^20^ The present study therefore employs time-resolved Fe Kβ X-ray emission spectroscopy (XES),^21–25^ an ultrafast technique that has proven to be a powerful complement to optical pump–probe spectroscopy due to its sensitivity to the nature of MC excited states.^20,26–28^

**Figure 1:**
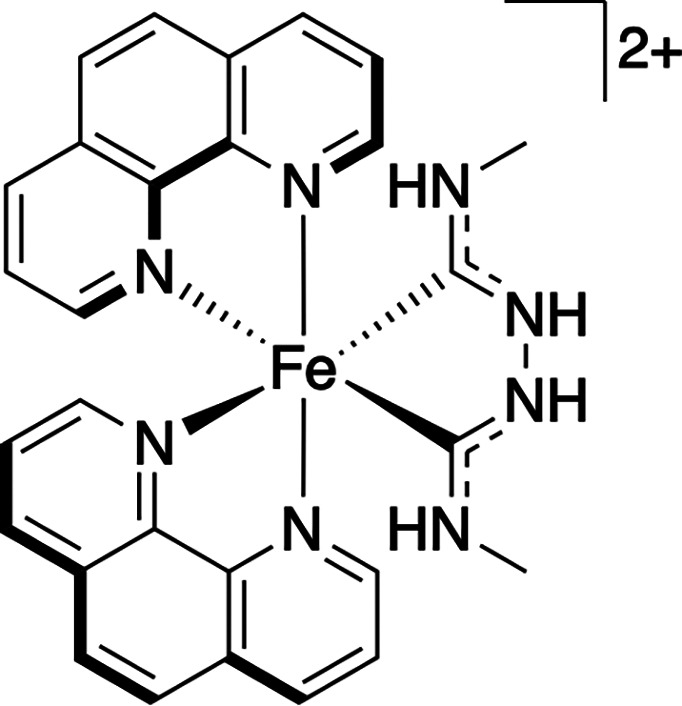
Molecular structure of [Fe­(phen)_2_(C_4_H_10_N_4_)]^2+^ (**1**).

Here, we present a time-resolved Fe Kβ XES study of complex **1**, utilizing both the valence-to-core (VtC) and Kβ mainline regions, and demonstrate that this technique can unambiguously elucidate the role of MC excited states in the excited state relaxation dynamics. The time-resolved Kβ XES data provide a detailed understanding of the excited state relaxation dynamics of complex **1** and enable a more definitive assignment of the excited states responsible for ground state recovery than could be achieved using time-resolved optical absorption data alone. These findings are complemented by variable-temperature transient optical absorption (VT-TA) measurements, which are fully consistent with the time-resolved Kβ XES data and provide additional insight into the thermodynamic parameters governing the ground state recovery dynamics. Together, these methods support an assignment of the long-lived excited state to a triplet MC (^3^MC) state rather than an MLCT state.

Density functional theory (DFT) calculations were further performed to clarify why complex **1** and the structurally similar complex Fe­(phen)_2_(CN)_2_ exhibit such distinct excited state dynamics. While our original report^19^ suggested that the increase in ligand field strength upon replacing two cyanide groups with the more strongly donating bidentate carbene ligand was largely responsible, the calculations in this study indicate that the increased structural rigidity of the bidentate carbene ligand plays a previously underappreciated role in destabilizing the quintet MC (^5^MC) excited state of complex **1** relative to that of Fe­(phen)_2_(CN)_2_. Specifically, the bidentate carbene ligand in complex **1** significantly restricts distortions of both the Fe–C bond lengths and C–Fe–C bond angle required to accommodate the high-spin configuration. These degrees of freedom are not as critical for structural relaxation of the ^3^MC excited state because the geometric difference between the ^1^A_1_ ground state and the ^3^MC excited state emphasizes distortion along a single bonding axis due to the pronounced Jahn–Teller effect associated with the ^3^MC configuration, and this distortion can be accommodated along an axis associated with weaker ligand field Fe–N coordinates. These conclusions are reinforced by the VT-TA measurements, which suggest a smaller degree of structural rearrangement associated with ground state recovery in complex **1** than in other first-row transition metal complexes in which a ^3^MC state is the lowest-energy excited state. The combination of detailed excited state mechanistic studies and DFT calculations used in this study highlights how the application of a suite of techniques, in particular the synergistic use of ultrafast X-ray and optical methods together with computational approaches, can assist in identifying design principles for controlling excited state dynamics of first-row transition metal-based chromophores.

## 2 Results and Analysis

### 2.1 VT-TA Spectroscopy

The initial report on the photophysical properties of complex **1**, summarized in the Introduction, was part of a study examining the effect of ligand field strength on excited state dynamics of Fe­(II) complexes following ^1^A_1_ → singlet MLCT (^1^MLCT) excitation.^19^ Replacement of one phen ligand in [Fe­(phen)_3_]^2+^ with two cyanide ligands to generate Fe­(phen)_2_(CN)_2_, a modification that increases the overall effective ligand field strength imposed on the metal center, affects the rate constants for both MLCT-to-MC conversion and ground state recovery but leaves the excited state relaxation mechanism qualitatively unchanged.^19,27^ Upon replacing the two cyanide ligands with a bidentate Chugaev-type carbene ligand to generate complex **1**, the photophysics changed dramatically, both qualitatively and quantitatively. Specifically, an increase of nearly 2 orders of magnitude in the rate constant of ground state recovery for complex **1** relative to Fe­(phen)_2_(CN)_2_, coupled with a pronounced change in the excited state absorption profile, strongly suggests that the nature of the excited state driving ground state recovery is no longer the ^5^T_2_ state that characterizes both [Fe­(phen)_3_]^2+^ and Fe­(phen)_2_(CN)_2_. Three possible scenarios were proposed: (1) the increase in ligand field strength imposed by the carbene ligand is sufficient to push the complex past the ^5^T_2_/^3^T_1_ crossing point, thereby stabilizing the latter as the lowest-energy excited state; (2) the increase in ligand field strength is sufficient to invert the relative energies of the MC and MLCT manifolds, positioning a ^3^MLCT state as the lowest-energy excited state; or (3) an isoenergetic condition in which the ^3^T_1_ and ^3^MLCT excited states are in quasi-equilibrium. A fourth possibility, in which the ^5^T_2_ state remains the lowest-energy excited state, seemed unlikely but still possible as an explanation for the observed dynamics. The information obtained from room-temperature time-resolved optical absorption data alone was simply insufficient to discriminate among these scenarios. In the present study, we have employed complementary time-resolved Kβ XES and VT-TA spectroscopy to elucidate the nature of the lowest-energy excited state of complex **1**. The VT-TA results presented in this section complement the time-resolved Kβ XES results that form the primary focus of Section 2.2. Notably, we have recently shown that VT-TA spectroscopy is a powerful tool for providing unique insight into the excited state dynamics of transition metal-based chromophores, including information on the spin state and structure of the excited states.^29–31^

Our analysis of the VT-TA data typically employs several theoretical approaches, including an Arrhenius model, transition state theory, and semiclassical Marcus theory. Here, we focus on the results based on transition state theory; a more comprehensive examination of the temperature-dependent photophysics is beyond the scope of the present study and will be reported elsewhere. The room-temperature time-resolved broadband optical absorption data that we previously reported for complex **1** showed a ground state bleach at wavelengths shorter than ∼625 nm and excited state absorption(s) at longer wavelengths,^19^ motivating the probe wavelength selection for the VT-TA measurements in the present study (see SI Note 1.2 for additional details). The kinetic traces measured at 580 nm following 500 nm excitation over the 235–295 K temperature range are shown in Figure 2a. These data cannot be fit with a single-exponential decay model but instead exhibit biphasic kinetics at each temperature, consistent with the two temporally resolved spectral components identified from a global analysis of the aforementioned room-temperature data.^19^ The fast decay component yields a temperature-independent time constant of 0.80 ± 0.25 ps, which is within experimental error of the value obtained from the time-resolved Kβ XES data described below (0.61 ps; see Section 2.3) and consistent with the room-temperature data reported previously.^19^ The absence of a temperature dependence for this fast decay component is consistent with expectations of highly nonequilibrium dynamics on the subpicosecond time scale.^32^

**Figure 2:**
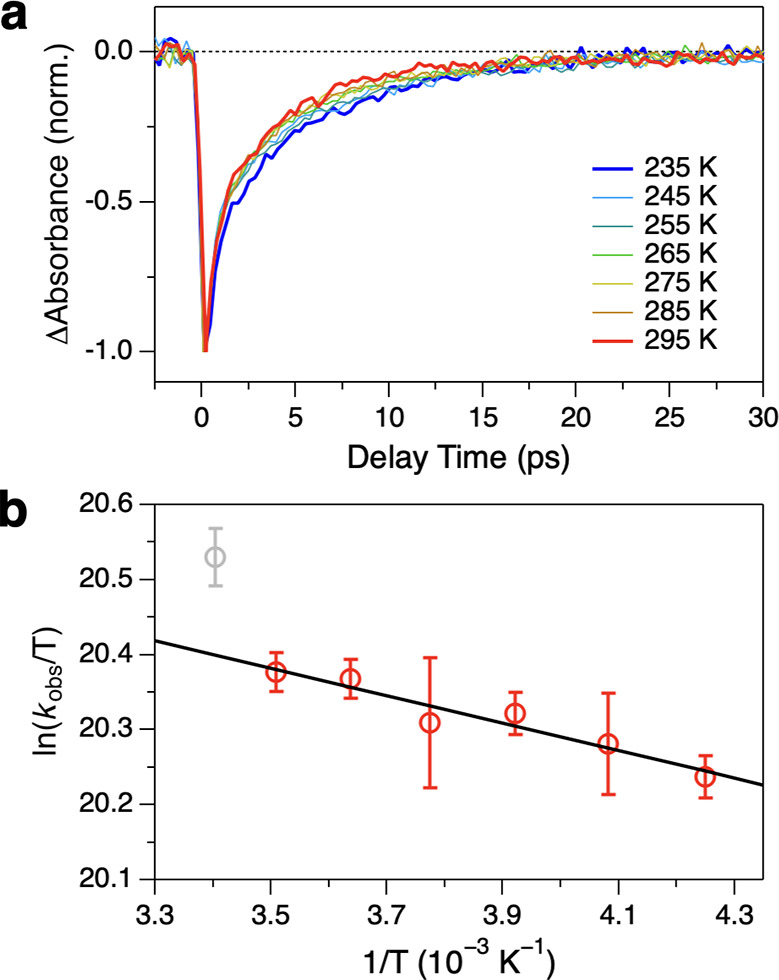
VT-TA data of complex **1** in methanol. (a) Temperature-dependent single-wavelength kinetic traces at 580 nm following excitation at 500 nm. All kinetic traces were fit to a biexponential decay model. (b) Eyring plot of the slow decay component obtained from the biexponential fits to the kinetic traces shown in (a). The slope and *y*-intercept yield Δ*H* ^‡^ = 260 ± 40 cm^–1^ and Δ*S* ^‡^ = −2.0 ± 0.1 cm^–1^ K^–1^, respectively. The 295 K data point (gray) was compromised by sample conditions (see SI Note 1.2). It is shown here for full disclosure but was omitted from the Eyring analysis.

The slow decay component, however, does exhibit a systematic temperature dependence, varying by ∼50% across the 60 K temperature range sampled. The data can be modeled using the Eyring equation:

1 $$k_{\mathrm{obs}}=\kappa \frac{k_{B}T}{h}\exp\left(-\frac{\Delta G^{‡}}{k_{B}T}\right)=\kappa \frac{k_{B}T}{h}\exp\left(\frac{\Delta S^{‡}}{k_{B}}\right)\exp\left(-\frac{\Delta H^{‡}}{k_{B}T}\right)$$

where *k*_obs_ is the observed rate constant; κ is the transmission coefficient (assumed to be 1); *k*_B_ is the Boltzmann constant; *T* is the temperature; *h* is the Planck constant; and Δ*G* ^‡^, Δ*S* ^‡^, and Δ*H* ^‡^ are the Gibbs free energy, entropy, and enthalpy of activation, respectively. The Eyring plot (ln­(*k*_obs_/*T*) vs 1/*T*) linearizes the data, allowing Δ*H* ^‡^ and Δ*S* ^‡^ to be extracted from the slope and *y*-intercept, respectively (Figure 2b). The modest (but clearly observable) change in the rate constant is indicative of a relatively small enthalpic barrier for ground state recovery. Indeed, the value of Δ*H* ^‡^ for complex **1** (260 ± 40 cm^–1^) lies between those of [Fe­(bpy)_3_]^2+^ (125 ± 20 cm^–1^; bpy = 2,2′-bipyridine)^29,33^ and [Fe­(terpy)_2_]^2+^ (465 ± 10 cm^–1^; terpy = 2,2′:6′,2″-terpyridine)^29,33^ and is significantly smaller than values reported for isoelectronic Co­(III) complexes, including that of [Co­(4,4′-OMe-bpy)_3_]^3+^ (900 ± 30 cm^–1^).^30^ It should be noted that ground state recovery in these two Fe­(II) polypyridyl complexes and this Co­(III) complex arises from excited states of decidedly different character: ^5^T_2_ for the Fe­(II) polypyridyls and ^3^T_1_ for the Co­(III) analogue. The intricate interplay of factors governing the magnitude of Δ*H* ^‡^, including excited state and ground state zero-point energies and geometric differences, makes a detailed mechanistic interpretation of Δ*H* ^‡^ difficult.

In contrast, Δ*S* ^‡^ provides significant interpretive value for assessing excited state character. This derives from the physical origins of the sign and magnitude of Δ*S* ^‡^, coupled with the ability to compare excited state properties with those of well-defined reference complexes. MC excited states of six-coordinate low-spin d^6^ metal complexes are interconfigurational in nature and are characterized by transfer of one or more electrons from nominally t_2g_ orbitals to σ* antibonding e_g_ orbitals. Consequently, the equilibrium geometries associated with the MC excited states exhibit longer metal–ligand bond lengths (and thus lower frequency metal–ligand vibrational modes) and larger molecular volumes than the ground state. Relaxation back to the ground state from the MC excited states therefore involves a reduction in both molecular volume and the magnitude of the vibrational partition function; both effects manifest as negative entropies of activation (Δ*S* ^‡^ < 0) for ground state recovery. Studies of spin-crossover complexes, in which the energy difference between the ^1^A_1_ and ^5^T_2_ states is on the order of *k*_B_ *T*, thereby allowing their relative populations to be thermally modulated, exhibit Δ*S* ^‡^ values in the range of −5 to −6 cm^–1^ K^–1^; this provides a useful benchmark for relaxation dynamics from a ^5^MC excited state to a singlet ground state.^34^ In contrast, VT-TA studies of Co­(III) polypyridyl complexes,^30^ as well as recent work on an Fe­(II) polypyridyl carbene complex,^31^ reported Δ*S* ^‡^ values in the range of −3 to −4 cm^–1^ K^–1^. This has been interpreted as indicative of relaxation from an *S* = 1 excited state (e.g., ^3^T_1_), with the smaller magnitude of the Δ*S* ^‡^ value attributed to the (t_2g_)^5^(e_g_)^1^ configuration of this state as opposed to the (t_2g_)^4^(e_g_)^2^ configuration that characterizes the ^5^T_2_ state. While less information is available concerning MLCT states, recent work indicated Δ*S* ^‡^ values in the range of 0 to −1 cm^–1^ K^–1^ for ground state recovery from such excited states,^35^ consistent with the minimal structural differences expected between the ^1^A_1_ ground state and the MLCT excited states. The Δ*S* ^‡^ value for complex **1** obtained from the data shown in Figure 2b is −2.0 ± 0.1 cm^–1^ K^–1^ and therefore implicates a lowest-energy MC excited state that would be *S* = 1 in character. Interestingly, the magnitude of Δ*S* ^‡^, while clearly inconsistent with either a ^5^T_2_ or MLCT state, is nevertheless appreciably smaller than what has been observed for the Co­(III) or Fe­(II) reference complexes. We shall return to this point in the Discussion below.

### 2.2 Kβ XES

Fe Kβ XES measures the X-ray emission generated by the electronic relaxation from occupied orbitals into the Fe 1s core hole following 1s electron ionization with high-energy X-rays.^36–43^ Kβ XES is divided into two spectral regions: the Kβ mainline and VtC regions. Figure 3a presents the Kβ XES spectrum of ground state complex **1**, showing both the Kβ mainline and VtC regions. The Kβ mainline region originates from metal 3p → 1s transitions. This region is predominantly sensitive to the metal spin state, as the 3p–3d exchange interaction in the final state splits the Kβ mainline emissions into the Kβ_1,3_ and Kβ′ features. This spin sensitivity is illustrated in Figure 3b, which shows the Kβ mainline XES spectra of ground state complex **1** (Fe *S* = 0, singlet state) and Fe reference complexes having Fe *S* = 1/2, 1, 3/2, and 2 (doublet, triplet, quartet, and quintet states, respectively).^44^ As the Fe spin state increases, the intense Kβ_1,3_ feature at ∼7058 eV shifts to higher energy, and the Kβ′ feature at ∼7045 eV clearly emerges and gains intensity. The reference difference spectra, obtained by subtracting the singlet state spectrum from the higher spin state spectra, exhibit the characteristic signatures for the different spin states (Figure 3c) and can thus be used to probe the excited state electronic structure from the vantage point of the Fe spin multiplicity.^25^

**Figure 3:**
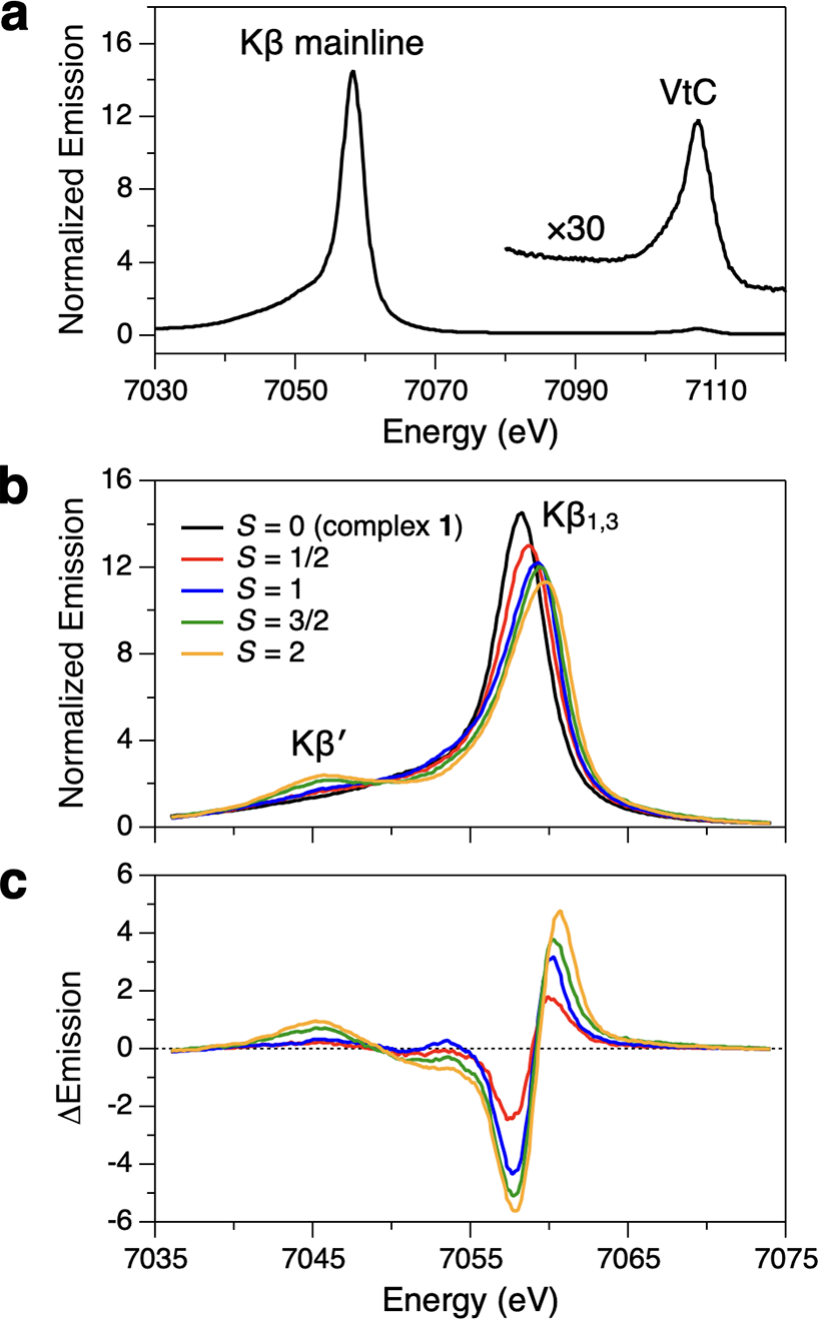
(a) Kβ XES spectrum of ground state complex **1** in methanol. Note the comparatively small cross section for VtC emission (VtC region expanded by ×30). (b) Kβ mainline XES spectra of ground state complex **1** (Fe *S* = 0; black) in methanol and the following Fe reference complexes: [Fe­(bpy)_3_]^3+^ (Fe *S* = 1/2; red), Fe­(II) phthalocyanine (Fe *S* = 1; blue), Fe­(III) phthalocyanine chloride (Fe *S* = 3/2; green), and [Fe­(phen)_2_(NCS)_2_] (Fe *S* = 2; orange).^44^ (c) Kβ mainline XES reference difference spectra constructed by subtracting the singlet state spectrum of complex **1** from the higher spin state spectra shown in (b).

Emissive electronic relaxation from occupied valence orbitals to the metal 1s core hole gives rise to the VtC region. This region is further divided into the Kβ_2,5_ and Kβ″ features, which generally correspond to transitions from ligand *n*p- and *n*s-based donor orbitals, respectively. The VtC emission energy reflects the energy of the donor valence orbitals relative to the metal 1s core hole, making it sensitive to the ligand ionization energy and the metal oxidation state. Since the intensity of VtC emission is primarily governed by the electric dipole transition mechanism (metal p → s), the extent of metal p orbital character mixed into the donor valence orbitals strongly influences the intensity of the VtC transitions. Thus, the VtC region is sensitive to the ligand environment and can provide complementary information to that from the Kβ mainline region. It is noteworthy that DFT calculations can accurately reproduce VtC XES,^39,41,45^ enabling facile theoretical analysis without the need for reference spectra, or the multiplet or multireference computational methods generally used to calculate the Kβ mainline region.^36–38,40,42,46–49^ A major challenge in utilizing time-resolved VtC XES, however, is the inherently weak emission signal, as demonstrated by the significant intensity difference between the Kβ mainline and VtC emission features in Figure 3a. This has been overcome by emerging capabilities at X-ray free electron laser facilities and improvements in X-ray spectrometer throughput.^24^ Notably, the reliable use of time-resolved VtC XES has been demonstrated by our studies of the excited states of several Fe complexes,^50,51^ as well as by Sension et al. in their studies of cobalamin.^52,53^

#### 2.2.1 VtC XES

The two-dimensional time-resolved transient VtC XES difference spectra of complex **1** in the −0.2 to 0.4 ps delay time range are presented in Figure 4a. Building on the general discussion of VtC XES above, the observed emission features between 7095 and 7115 eV for ground state complex **1** (Figure 4b) arise from the filling of the Fe 1s core hole with electrons from frontier molecular orbitals predominantly derived from ligand-based valence orbitals that hybridize with Fe p-symmetry orbitals. As discussed by Ledbetter et al.,^50^ VtC XES is highly sensitive to changes in oxidation state and metal–ligand bond length; for example, higher oxidation state and longer bond lengths lead to spectral shifts to higher energy and a reduction in intensity, respectively. For complex **1**, the strong Fe–carbene σ-bonding interactions generate pronounced VtC XES spectral intensity in the ground state.

**Figure 4:**
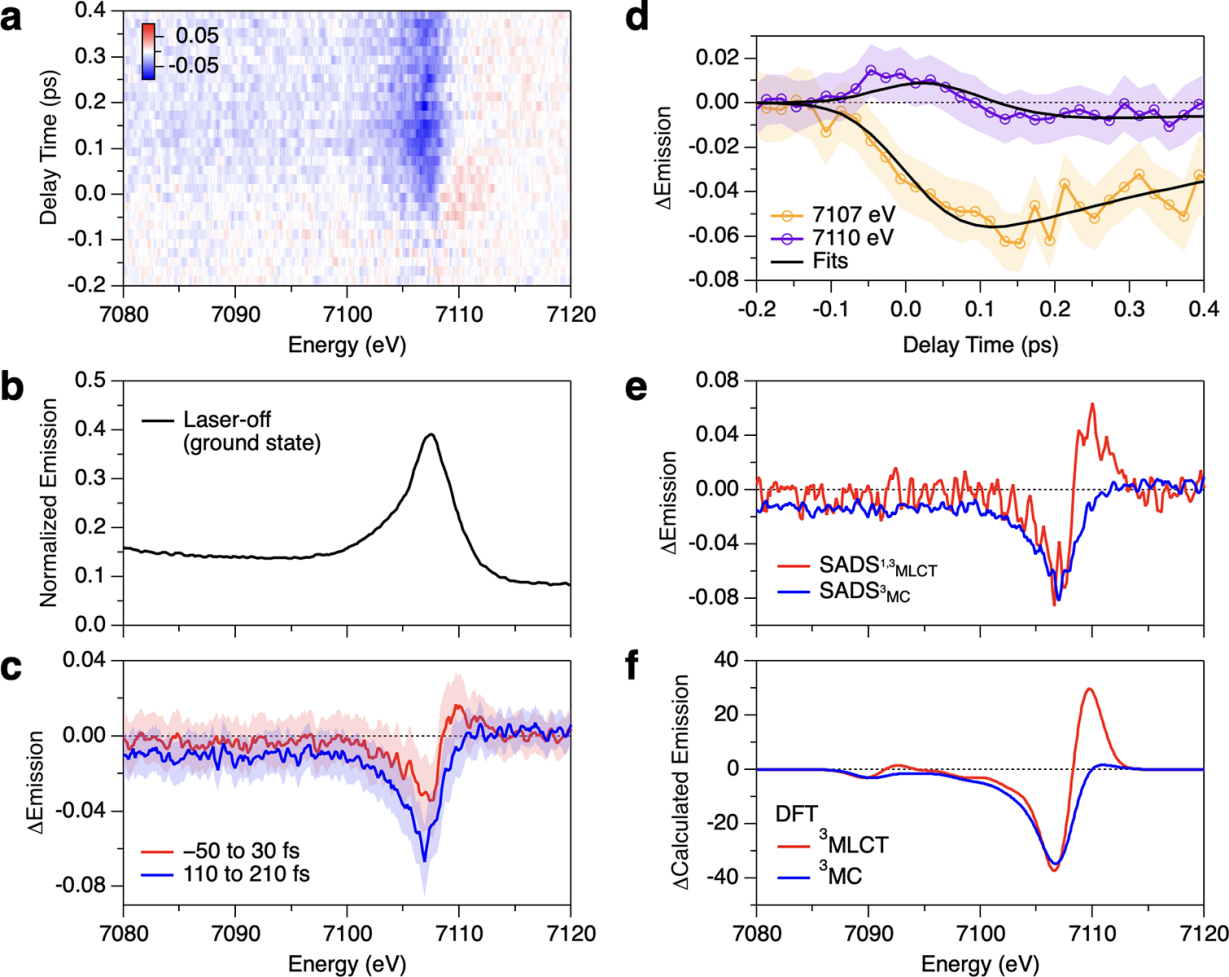
Time-resolved VtC XES data of complex **1** in methanol. (a) Two-dimensional transient VtC XES difference spectra in the −0.2 to 0.4 ps delay time range. (b) Laser-off VtC XES spectrum representing the ground state of complex **1**. (c) Transient VtC XES difference spectra averaged over the −50 to 30 fs and 110 to 210 fs delay time ranges. (d) Kinetic traces at 7107 and 7110 eV (averaged over 1.5 eV range for each energy), and fits to these kinetic traces from the global analysis (see SI Note 3). Shaded areas in (c) and (d) represent the standard deviation from the data averaging. (e) SADS of the ^1,3^MLCT and ^3^MC excited states derived from the global analysis (see SI Note 3). (f) DFT calculated VtC XES difference spectra of the ^3^MLCT and ^3^MC states.

The transient Kβ XES measurements were conducted using a 520 nm optical laser pump that generates an MLCT excited state.^19^ The transient difference data were obtained by subtracting the laser-off spectrum from the laser-on spectra at various delay times between the optical pump and the X-ray probe. The laser-off spectrum, representing the electronic ground state of complex **1**, shows a Kβ_2,5_ feature with a maximum intensity at ∼7107 eV (Figure 4b). The two-dimensional transient VtC XES difference spectra are dominated by a negative feature at ∼7107 eV (Figure 4a). Careful examination reveals a short-lived positive feature at ∼7110 eV, which is clearly observed in the transient difference spectrum at early delay times (averaged over −50 to 30 fs; red spectrum in Figure 4c) and in the kinetic trace at 7110 eV (purple trace in Figure 4d). This positive feature decays within ∼100 fs, after which the transient difference spectrum is characterized by an overall negative feature, as shown in the spectrum averaged over 110 to 210 fs (blue spectrum in Figure 4c).

We performed a global analysis of the transient VtC XES difference data shown in Figure 4a, based on a kinetic model for the excited state relaxation mechanism developed in Section 2.3 by incorporating both the VtC and Kβ mainline XES data (see SI Note 3 for details of this global analysis). The global analysis results, as shown by the two-dimensional fit and residual (Figure S8a) as well as the kinetic trace fits (Figure 4d), demonstrate the quality of the fit obtained. We discuss the kinetic modeling and fit in Section 2.3. Here, we focus on the spectral analysis and excited state assignments.

The species associated difference spectra (SADS) from the global analysis are presented in Figure 4e. These SADS can be compared with DFT calculated difference spectra for assignments of the excited states (see SI Note 2 for details of the DFT calculations which were performed with ORCA version 5.0.3 ^54,55^ ). We note that the calculated VtC XES spectrum of ground state complex **1** reproduces the experimental laser-off spectrum (Figure S6), validating the use of the DFT calculations in the analysis of the VtC XES data. The calculated VtC XES difference spectra for the ^3^MLCT and ^3^MC states are shown in Figure 4f (see Figure S7a for the calculated VtC XES spectra). The SADS of the ^1,3^MLCT excited state closely resembles the calculated difference spectrum for the ^3^MLCT state (red spectra in Figure 4e,f), confirming the assignment of the initial excited state to the ^1,3^MLCT excited state. Note that VtC XES is insensitive to the spin orientation of the electron promoted to the ligand in the MLCT excited state and therefore unable to differentiate the ^1^MLCT and ^3^MLCT excited states. In the ^1,3^MLCT excited state, CT from the Fe center to the ligand increases the effective nuclear charge on Fe, resulting in an overall shift of the VtC XES features to higher energy (as shown in the calculated red spectrum in Figure S7a). This is reflected as negative and positive features at 7107 and 7110 eV, respectively, in the SADS of the ^1,3^MLCT excited state (red spectrum in Figure 4e; also seen in the −50 to 30 fs averaged spectrum in Figure 4c).

The SADS we assigned to a ^3^MC excited state agrees well with the calculated difference spectrum for the lowest-energy ^3^MC state (blue spectra in Figure 4e,f), suggesting that the ^1,3^MLCT excited state decays to ^3^MC excited states. The Fe center in complex **1** exhibits pseudo-octahedral symmetry and thus, in the ^3^MC excited state, a tetragonal pseudo-Jahn–Teller distortion can occur with elongation of the Fe–ligand_axial_ bonds (see SI Note 2.1). This bond elongation reduces Fe p orbital mixing into the ligand-based donor valence orbitals, leading to a decrease in the intensity of the VtC emission features (as shown in the calculated blue spectrum in Figure S7a). This is reflected as an overall negative feature in the SADS of the ^3^MC excited state (blue spectrum in Figure 4e; also seen in the 110 to 210 fs averaged spectrum in Figure 4c).

We also simulated the VtC XES spectrum for the ^5^MC state (orange spectrum in Figure S7a). In the ^5^MC state, the Fe–ligand bonds are symmetrically expanded (as evidenced by the DFT calculations in SI Note 2.1), resulting in an additional decrease in the intensity of the VtC emission features (as shown in the calculated orange spectrum in Figure S7a). While the calculated difference spectrum for the ^5^MC state (orange spectrum in Figure S7b) may appear to be similar to the SADS of the ^3^MC excited state (blue spectrum in Figure 4e), the analysis of the Kβ mainline XES data presented in Section 2.2.2 clearly indicates that the dominant relaxation pathway involves ^1,3^MLCT → ^3^MC, rather than ^1,3^MLCT → ^5^MC.

#### 2.2.2 Kβ Mainline XES

Figure 3a,b shows the Kβ mainline XES spectrum of ground state complex **1**. This represents the singlet ground state of complex **1** and is similar to those of other low-spin Fe­(II) complexes.^20,26,44,56–58^ The strong exchange interaction between Fe 3d and 3p electrons in the final state probed by Kβ mainline XES makes this technique sensitive to the Fe spin moment (as discussed above) and provides an effective approach to characterizing spin state dynamics of first-row transition metal complexes. We have established the use of Kβ mainline XES spectra from reference complexes with well-defined, distinct ground state spin configurations to model excited states with different spin multiplicities.^44^ The justification for, and limitations of, using ground state spectra to model excited states have been discussed by Gaffney.^25^

The transient Kβ mainline XES difference data of complex **1** in the −0.2 to 0.4 ps delay time range, measured simultaneously with the transient VtC XES difference data discussed in Section 2.2.1, predominantly exhibit the spectral signature of a ^3^MC excited state (Figures 5a and S9b; see SI Note 4 for analysis of these transient difference data). While the transient VtC XES difference data clearly reveal the short-lived ^1,3^MLCT excited state, this state is not resolved in the transient Kβ mainline XES difference data. This is expected for three reasons: (1) the ^1,3^MLCT excited state has the weakest difference spectrum in the Kβ mainline region (Figure 3c), in contrast to the VtC region; (2) the ultrashort lifetime, relative to the temporal resolution, suppresses the ^1,3^MLCT excited state signal; and (3) the Kβ mainline XES difference spectra for Fe doublet and triplet states differ primarily in amplitude rather than shape (Figure 3c), making them difficult to differentiate when the kinetics and temporal resolution do not enable them to be separated temporally.

**Figure 5:**
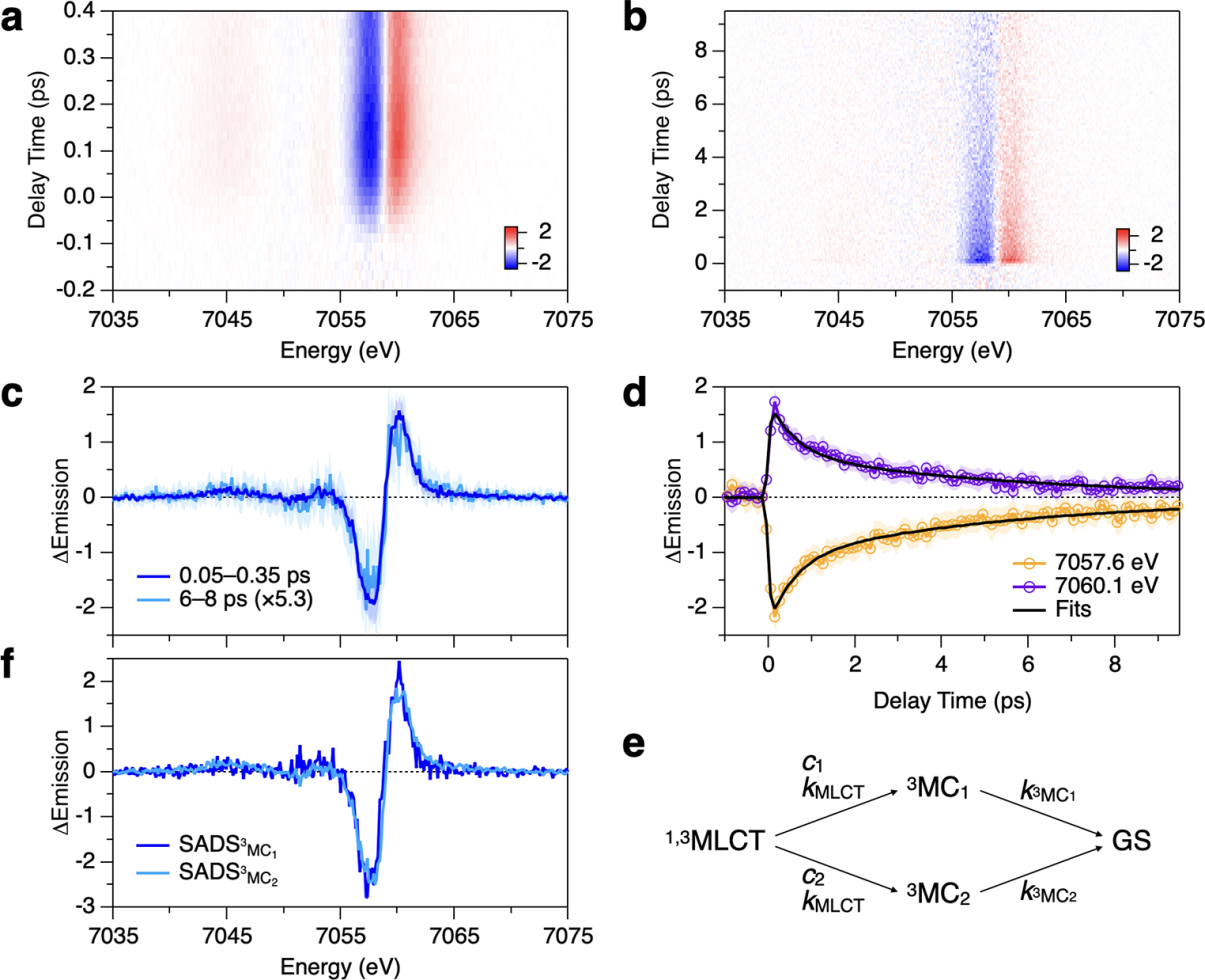
Time-resolved Kβ mainline XES data of complex **1** in methanol. Two-dimensional transient Kβ mainline XES difference spectra in the (a) −0.2 to 0.4 ps and (b) −1.0 to 9.5 ps delay time ranges. (c) Transient Kβ mainline XES difference spectra averaged over the 0.05 to 0.35 ps and 6 to 8 ps delay time ranges from the two-dimensional data shown in (b). The 6 to 8 ps averaged spectrum is multiplied by 5.3 to match the magnitude of the 0.05 to 0.35 ps averaged spectrum. (d) Kinetic traces at 7057.6 and 7060.1 eV (averaged over 1 eV range for each energy), and fits to these kinetic traces from the global analysis (see SI Note 5.2). Shaded areas in (c) and (d) represent the standard deviation from the data averaging. (e) Proposed kinetic model for the dominant excited state relaxation dynamics of complex **1**. GS denotes the ground state. (f) SADS of the ^3^MC_1_ and ^3^MC_2_ excited states from the global analysis (see SI Note 5.2).

The two-dimensional transient Kβ mainline XES difference spectra of complex **1** in the −1.0 to 9.5 ps delay time range are presented in Figure 5b. Two aspects of these spectra should be emphasized. First, the spectral shape of the difference signal remains unchanged over time. This is demonstrated in Figure 5c, where the transient difference spectrum averaged over the 0.05 to 0.35 ps delay time range closely matches that averaged over the 6 to 8 ps range (after scaling). Notably, both spectra are well fit with the triplet reference difference spectrum but not with the quintet spectrum (Figure S10). Second, the kinetic traces at 7057.6 and 7060.1 eV exhibit clear biexponential decays (Figure 5d). These observations, together with the VtC XES analysis in Section 2.2.1, indicate that the initial ^1,3^MLCT excited state rapidly decays to two dynamically distinct but spectroscopically indistinguishable ^3^MC excited states, which we denote ^3^MC_1_ and ^3^MC_2_, that subsequently relax to the ground state. Indeed, within the limitations of Kβ mainline XES, we do not expect distinct ^3^MC states, for example the ^3^T_1_ and ^3^T_2_ excited states or zero-field split components of the ^3^T_1_ state (see the Discussion), to produce measurably different Kβ mainline XES spectra. Constrained by this assumption, we propose that the observed biexponential decays results from parallel relaxation pathways involving the ^3^MC_1_ and ^3^MC_2_ excited states, each decaying to the ground state with a different lifetime. Additionally, the assumption that all ^3^MC states have indistinguishable Kβ mainline XES spectra enables us to rule out a sequential decay mechanism (e.g., ^1,3^MLCT → ^3^MC_1_ → ^3^MC_2_ → ground state). A sequential decay mechanism would exhibit a single exponential decay in the kinetic traces, because the transition from the ^3^MC_1_ to ^3^MC_2_ excited state is not expected to change the total population of the ^3^MC excited states and consequently would not be reflected in the kinetic traces.

### 2.3 Kinetic Modeling and Excited State Relaxation Dynamics

Based on the analysis of the VT-TA and time-resolved Kβ XES data presented in Sections 2.1 and 2.2, a kinetic model for the excited state relaxation dynamics of complex **1** can be proposed (Figure 5e). In this kinetic model, the initial ^1,3^MLCT excited state, formed by the optical pump, rapidly decays to the ^3^MC_1_ and ^3^MC_2_ excited states via parallel relaxation pathways, with a branching ratio of *c*_1_:*c*_2_. These two distinct ^3^MC excited states subsequently relax back to the ground state with different rate constants ( kMC13 and kMC23 ), resulting in the observed biexponential decay profiles.

The global analysis of the transient VtC XES difference data shown in Figure 4a, briefly discussed in Section 2.2.1, yields a lifetime of ≤60 fs for the ^1,3^MLCT excited state (see SI Note 3 for details). The temporal resolution and signal-to-noise ratio preclude the reliable identification of lifetimes shorter than ∼60 fs (see SI Note 3 for details). Based on the parallel decay kinetic model (Figure 5e), a global analysis was also performed for the transient Kβ mainline XES difference data in the −1.0 to 9.5 ps delay time range presented in Figure 5b (see SI Note 5.2 for details). The two-dimensional fit and residual (Figure S11a) as well as the kinetic trace fits (Figure 5d) demonstrate the quality of the fit. From this global analysis, lifetimes of the ^3^MC_1_ and ^3^MC_2_ excited states are determined to be 0.61 and 5.7 ps, respectively, with a branching ratio of *c*_1_ = 52% and *c*_2_ = 48% (Table S4). The SADS of the ^3^MC_1_ and ^3^MC_2_ excited states are nearly identical (Figure 5f) and are well fit with the triplet reference difference spectrum but not with the quintet spectrum (Figure S11b), confirming that the two ^3^MC excited states are indeed generated from the initial ^1,3^MLCT excited state and subsequently decay. SI Note 4 presents a global analysis of the transient Kβ mainline XES difference data in the −0.2 to 0.4 ps delay time range shown in Figure 5a, providing insights into the full width at half-maximum of the instrument response function and the excitation fraction.

The analysis of the transient VtC and Kβ mainline XES difference data reveals the dominant excited state relaxation mechanism for complex **1**: ^1,3^MLCT → ^3^MC_1_ + ^3^MC_2_ → ground state (Figure 5e). To further investigate the excited state at longer delay times, we generated a transient Kβ mainline XES difference spectrum averaged over the 20 to 50 ps delay time range (black spectrum in Figure 6). Inspection of this spectrum clearly shows that the decay of the ^3^MC_1_ and ^3^MC_2_ excited states leaves a weak but persistent difference signal. At delay times ≥20 ps, the population of the ^3^MC excited states is estimated to be <1% and is therefore unlikely to account for this signal.^59^ Fits to this persistent difference signal with the doublet, triplet, and quintet reference difference spectra show that the quintet spectrum provides the best match, based on both visual fit and the highest *R* ^2^ value (Figure 6). This suggests the presence of a minor relaxation pathway in which the ^1,3^MLCT excited state decays to a ^5^MC excited state with a significantly longer lifetime. Ultrafast formation of a ^5^MC excited state following bifurcation from an initially formed ^1^MLCT excited state has recently been demonstrated in [Fe­(bpy)_3_]^2+^ via ultrafast two-dimensional electronic spectroscopy.^60^ The longer lifetime of the ^5^MC excited state is consistent with the expected Δ*S* = 2 nature of its relaxation to the singlet ground state. We interpret the presence of a weak ^5^MC excited state population to indicate parallel ultrafast formation of ^3^MC and ^5^MC excited states from the optically generated ^1,3^MLCT excited state.

**Figure 6:**
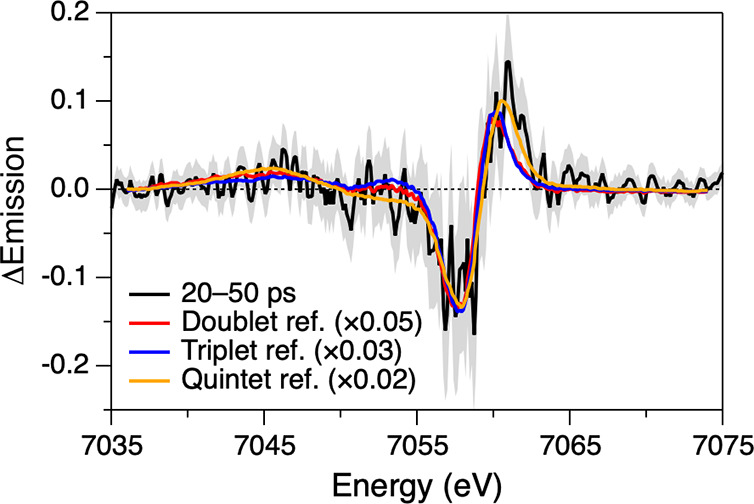
Transient Kβ mainline XES difference spectrum of complex **1** in methanol, averaged over the 20 to 50 ps delay time range, and fits to this spectrum with the doublet, triplet, and quintet reference difference spectra. The *R* ^2^ values are 0.689, 0.687, and 0.806 for the doublet, triplet, and quintet fits, respectively. Shaded areas represent the standard deviation from the data averaging.

A comprehensive mechanism for excited state relaxation dynamics of complex **1**, including the major and minor decay pathways, can now be constructed by combining all of the observations discussed above. The fraction of ^1,3^MLCT excited states forming ^5^MC excited states can be estimated from the fit of the quintet reference difference spectrum to the 20 to 50 ps averaged transient difference spectrum, which finds 0.02 as a scaling factor (Figure 6). Based on this scaling factor and the excitation fraction (65%; see SI Note 4 for details of the excitation fraction determination), it is estimated that 3% of the initial ^1,3^MLCT excited state decays to the ^5^MC excited state. Accounting for this minor decay pathway, the branching ratio to the ^3^MC_1_ and ^3^MC_2_ excited states becomes 50%:47%. The schematic excited state relaxation dynamics of complex **1** is presented in Figure 7.

**Figure 7:**
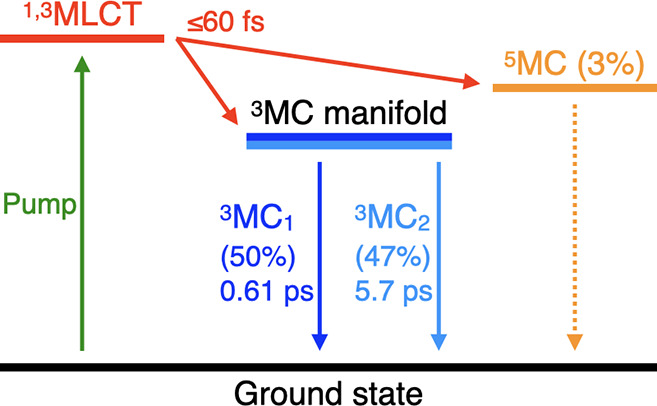
Schematic excited state relaxation dynamics of complex **1** revealed by time-resolved Kβ XES together with VT-TA spectroscopy. The diagram is qualitative; in particular, the vertical placement of the two ^3^MC excited states within the ^3^MC manifold is schematic and does not indicate their relative energies. The decay of the ^5^MC excited state is indicated with a dotted arrow, but its lifetime is not resolved in this study.

## 3 Discussion

In this study, we have elucidated the excited state structure and relaxation dynamics of complex **1** using time-resolved Fe Kβ XES together with VT-TA spectroscopy, demonstrating the utility and complementarity of these experimental techniques for investigating the photoinduced dynamics of transition metal complexes. Specifically, time-resolved Kβ XES enables clear identification of MC (and MLCT) excited states, while VT-TA spectroscopy provides insight into the nature of the excited states from the sign and magnitude of Δ*S* ^‡^. The ∼7 ps kinetic component previously observed in optical TA spectroscopy^19^ can now be assigned to the decay of a ^3^MC excited state, as it is generally consistent with the 5.7 ps lifetime of the ^3^MC_2_ excited state determined in this study (Section 2.3). It is noteworthy that we recently reported a time-resolved XES study of an Fe amido complex, in which the long-lived MLCT excited state assigned from optical TA spectroscopy was reinterpreted as the ^5^MC excited state.^20^ The present study further underscores the need for caution when interpreting optical TA spectroscopy data and highlights that time-resolved XES can provide valuable and detailed information regarding excited state structure and dynamics.

For complex **1**, a parallel decay mechanism involving two dynamically distinct ^3^MC excited states has been revealed. Relaxation of an MLCT excited state via a ^3^MC excited state, without significant involvement of a ^5^MC excited state, has been observed in Fe complexes bearing carbene ligands.^31,57,61–72^ In some related complexes, similar parallel decay mechanisms have been proposed, although the identity of the excited states involved may differ.^57,64,67–70^ As briefly noted in Section 2.2.2, the ^3^MC_1_ and ^3^MC_2_ excited states could correspond to the ^3^T_1_ and ^3^T_2_ states in an ideal octahedral ligand field. However, alternative assignments for the two ^3^MC excited states are possible, given that the Fe center in complex **1** does not exhibit perfect octahedral symmetry. In a study of the excited state dynamics of Fe­(II) carbene complexes, Lindh et al. noted a shallow ^3^MC potential energy surface with several local Jahn–Teller distorted minima that are coupled differently to the ground state, resulting in a distribution of decay times.^65^ In light of the significant difference in ligand field strength imposed by the phen and carbene ligands that define the N_4_C_2_ coordination environment of complex **1**, the two ^3^MC excited states identified from the observed biphasic kinetics could correspond to zero-field split components derived from the ^3^T_1_ state. In our previous study,^19^ the singular value decomposition analysis of the room-temperature optical TA data revealed three kinetic components with time constants of 0.15, 1.2, and 5.3 ps. The fastest component, which was at the limit of our temporal resolution, had spectral features consistent with expectations for an MLCT excited state, whereas the latter two components had time constants comparable to those of the two ^3^MC excited states identified in the present study (Section 2.3). The optical TA spectral profiles of these latter two components were similar, suggesting a common electronic origin and providing support for the possible zero-field split ^3^T_1_ parentage of the two ^3^MC excited states. At present, however, there is insufficient information (experimental or theoretical) to definitively determine the nature of the two ^3^MC excited states of complex **1**, or their relative energies. While further insight into these states would be of interest, it is beyond the scope of the present study.

As one of the ligand design strategies for achieving photoactive transition metal complexes with extended CT excited state lifetimes, heteroleptic systems combining polypyridyl ligands with strong field ligands have been adopted and investigated. In these systems, the polypyridyl ligands serve as light harvesting units that provide desirable optical properties, while the strong field ligands are used to control the excited state dynamics by modulating the ligand field strength and thereby the relative energetics of the CT and MC excited states. Among strong field ligands, the strongly σ-donating carbenes employed in complex **1** have been used to destabilize the e_g_ orbitals that have σ* antibonding metal–ligand character.^14–18^ Alternatively, strongly π-accepting cyanides have also been used to stabilize the t_2g_ orbitals having the π backbonding metal–ligand character.^26–28,73–76^ In both cases, the MC excited states are relatively destabilized with respect to the CT excited states, which can result in long-lived CT excited states.

We previously investigated the effect of cyanide ligand substitution on the excited state relaxation dynamics of Fe­(II) polypyridyl complexes. Specifically, [Fe­(bpy)_3_]^2+^ has exhibited the rapid decay of the initial MLCT excited state on a subpicosecond time scale to the ^5^MC excited state via the ^3^MC excited state.^44,77^ In the complex where one bpy ligand is replaced with two cyanide ligands, Fe­(bpy)_2_(CN)_2_, the relaxation dynamics remains similar to that of [Fe­(bpy)_3_]^2+^.^27^ For [Fe­(bpy)­(CN)_4_]^2–^, in which two bpy ligands are substituted with four cyanide ligands, the relaxation dynamics exhibits strong solvent dependence. In aprotic solvents, the MLCT excited state shows a long lifetime of 19 ps, without evidence of^3^MC or ^5^MC excited state formation.^26^ In contrast, in protic solvents, the MLCT excited state decays within 100 fs to form the ^3^MC excited state with a 13 ps lifetime.^28^ Given that the long-lived ^3^MC excited state is the dominant excited state species, the relaxation mechanism of complex **1** is analogous to that of [Fe­(bpy)­(CN)_4_]^2–^ in protic solvents. This is surprising because complex **1** is structurally more similar to Fe­(bpy)_2_(CN)_2_ than [Fe­(bpy)­(CN)_4_]^2–^. To understand the origin of this experimental finding, we performed a series of DFT calculations to examine how the ligand environment leads to this result.

Our previous study^19^ showed that the excited state relaxation mechanisms of [Fe­(phen)_3_]^2+^ and Fe­(phen)_2_(CN)_2_ are similar to those of [Fe­(bpy)_3_]^2+^ and Fe­(bpy)_2_(CN)_2_, respectively, as expected given that the polypyridyl phen ligand would behave similarly to the bpy ligand in the relaxation dynamics. Thus, DFT calculations were conducted for complex **1**, [Fe­(phen)_3_]^2+^, Fe­(phen)_2_(CN)_2_, and [Fe­(phen)­(CN)_4_]^2–^ (see Figures 8a and S15 for the B3LYP DFT optimized structures of the ground states of these complexes). It has been shown that functionals with a higher fraction of Hartree–Fock exchange tend to stabilize high-spin states relative to low-spin states.^78,79^ Therefore, we first conducted a benchmark study and found that the B3LYP functional is appropriate for comparing the excited state energetics of the Fe complexes considered in this study (SI Note 7.1). Using the B3LYP functional, the ^5^MC state is calculated to lie higher in energy than the ^3^MC state for complex **1** and [Fe­(phen)­(CN)_4_]^2–^, whereas for [Fe­(phen)_3_]^2+^ and Fe­(phen)_2_(CN)_2_, the ^5^MC state lies lower in energy than the ^3^MC state (Figure 8b and Table 1; see SI Note 7.2 for details of the B3LYP DFT results of these complexes). These results are consistent with the experimentally revealed role of MC excited states in the relaxation mechanisms and with the energetic landscape proposed in our previous study.^19^

**Figure 8:**
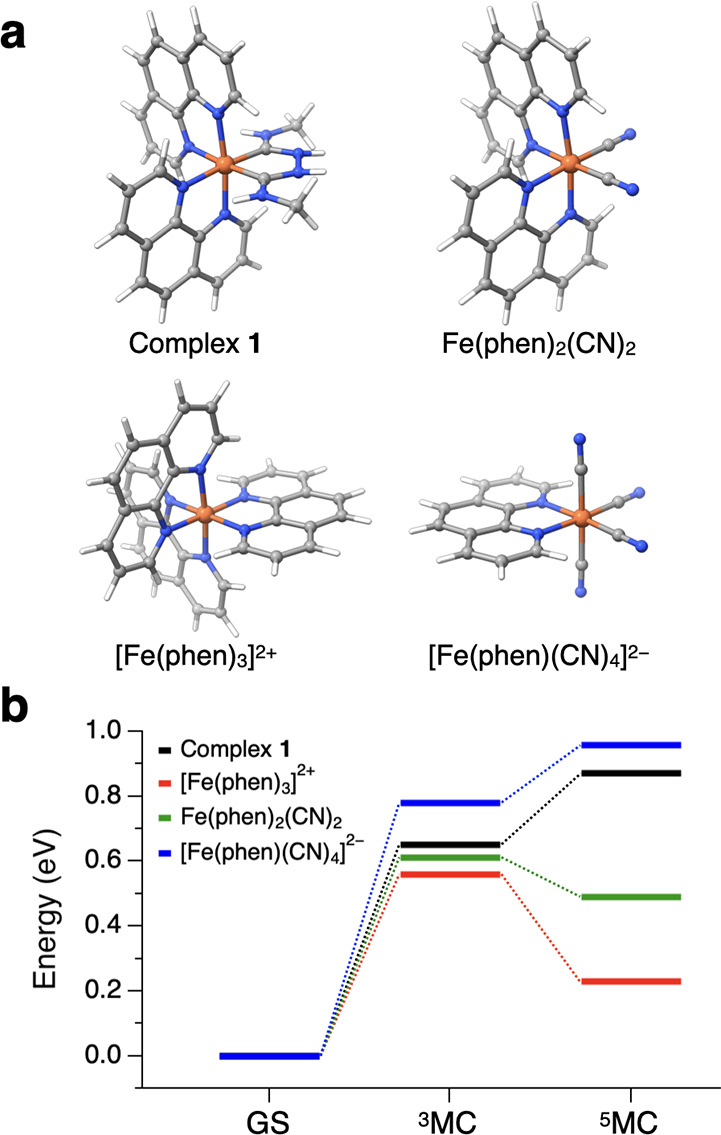
(a) B3LYP DFT optimized structures of the ground states of complex **1**, [Fe­(phen)_3_]^2+^, Fe­(phen)_2_(CN)_2_, and [Fe­(phen)­(CN)_4_]^2–^ (see SI Note 7.2 for details of the B3LYP DFT results of these complexes). Fe is shown in orange, N in blue, C in gray, and H in white. (b) Relative energies of the ^3^MC and ^5^MC states with respect to the ground state (GS), based on the B3LYP DFT optimized structures (Table 1).

**Table 1:** Relative Energies (eV) of the ^3^MC and ^5^MC States with Respect to the Ground State, Based on the B3LYP DFT Optimized Structures of the Fe Complexes

	^3^MC state	^5^MC state
Complex **1**	0.65	0.87
[Fe(phen)_3_]^2+^	0.56	0.23
Fe(phen)_2_(CN)_2_	0.61	0.49
[Fe(phen)(CN)_4_]^2–^	0.78	0.96
Modified complex **1** with monodentate carbenes^a^	0.49	0.50
[Fe(phen)_2_(CN^tBu^Ar_3_NC)]^2+^ ^b^	0.82	0.87
*trans*-[Fe(py)_2_(CN)_4_]^2–^	0.44	0.82
*cis*-[Fe(py)_2_(CN)_4_]^2–^	0.74	0.84

a For this complex, the N–N bond in the bidentate carbene ligand of complex **1** is cleaved to generate two monodentate carbene ligands.

b For this complex, the two monodentate cyanide ligands in Fe­(phen)_2_(CN)_2_ are replaced with a bidentate diisocyanide ligand.

In the B3LYP DFT calculations, the ^3^MC state energies of complex **1** and Fe­(phen)_2_(CN)_2_ are rather similar (0.65 and 0.61 eV, respectively), while the ^5^MC state energy is significantly higher for complex **1** (0.87 vs 0.49 eV) (Figure 8b and Table 1). We consider that this difference would arise from the differing degrees of structural rigidity in the ligand environments that originate from the denticity of the strong field ligands. Specifically, complex **1** contains the one bidentate carbene ligand while Fe­(phen)_2_(CN)_2_ has the two monodentate cyanide ligands (Figures 8a and S15a,c). Importantly, monodentate ligands provide greater flexibility in decoupling metal–ligand bond lengths and bond angles, which is not possible for a bidentate ligand. In particular, upon formation of the ^5^MC state from the ground state, which involves elongation of all Fe–ligand bonds, the C–Fe–C bond angle decreases by ∼4° for complex **1** (Table S6) but increases by ∼9° for Fe­(phen)_2_(CN)_2_ (Table S8). This likely reflects the increased structural constraint imposed by the bidentate carbene ligand in complex **1**, contributing to the higher energy of the ^5^MC state.

To examine the effect of increased structural rigidity imparted by bidentate strong field ligands, we performed DFT calculations on modified complexes that have analogous coordinating bonds to complex **1** and Fe­(phen)_2_(CN)_2_ but distinct extended ligand structures. For complex **1**, breaking the N–N bond in the bidentate carbene ligand generates a modified complex **1** which has two monodentate carbene ligands (Figures 9a and S16). The DFT calculations show that the ^5^MC state energy of this modified complex **1** is significantly decreased relative to that of complex **1** (0.50 vs 0.87 eV; Table 1 and Figure 9b; see SI Note 7.3 for details of the B3LYP DFT results of modified complex **1**), indicating that increased conformational freedom enables better optimization of the Fe–ligand bond lengths and bond angles in the ^5^MC state (including a ∼6° increase in the C–Fe–C bond angle, as shown in Table S10). Note that the ^5^MC state energy of modified complex **1** is comparable to that of Fe­(phen)_2_(CN)_2_ (0.50 vs 0.49 eV; Table 1 and Figure 9b). Albeit less pronounced, the ^3^MC state energy of modified complex **1** is also decreased compared to that of complex **1** (0.49 vs 0.65 eV; Table 1 and Figure 9b), suggesting that structural rigidity has some influence on the pseudo-Jahn–Teller distortion in the ^3^MC state. This could be reflected in the larger axial elongation in the ^3^MC state of modified complex **1** compared to complex **1** (ca. 0.34 vs 0.29 Å elongation; Tables S10 and S6). We have also compared Fe­(phen)_2_(CN)_2_ with [Fe­(phen)_2_(CN^tBu^Ar_3_NC)]^2+^, in which the two monodentate cyanide ligands are replaced with a bidentate diisocyanide ligand (Figures 9a and S17). It is noteworthy that Wenger and coworkers reported that a Cr(0) complex having this bidentate diisocyanide ligand, Cr­(CN^tBu^Ar_3_NC)_3_, exhibits a long-lived ^3^MLCT excited state (2.2 ns lifetime).^80^ Our DFT calculations predict a significantly increased ^5^MC state energy for [Fe­(phen)_2_(CN^tBu^Ar_3_NC)]^2+^ compared to that for Fe­(phen)_2_(CN)_2_ (0.87 vs 0.49 eV; Table 1 and Figure 9b; see SI Note 7.4 for details of the B3LYP DFT results of [Fe­(phen)_2_(CN^tBu^Ar_3_NC)]^2+^). This again highlights the impact of structural rigidity, where increased rigidity destabilizes the ^5^MC state by hindering simultaneous optimization of metal–ligand bond lengths and bond angles (including a ∼1° decrease in the C–Fe–C bond angle, as shown in Table S11). The ^5^MC state energy of [Fe­(phen)_2_(CN^tBu^Ar_3_NC)]^2+^ becomes comparable to that of complex **1** (0.87 vs 0.87 eV; Table 1 and Figure 9b). We note that, across these complexes, upon formation of the ^5^MC state from the ground state, the C–Fe–C bond angle decreases for the complexes containing the bidentate strong field ligands, whereas this angle increases for the complexes with the two monodentate strong field ligands. The ^3^MC state energy of [Fe­(phen)_2_(CN^tBu^Ar_3_NC)]^2+^ is also increased compared to that of Fe­(phen)_2_(CN)_2_ (0.82 vs 0.61 eV; Table 1 and Figure 9b), similar to the case of modified complex **1**, suggesting again that the pseudo-Jahn–Teller distortion in the ^3^MC state is affected by structural rigidity. These calculations for modified complex **1** and [Fe­(phen)_2_(CN^tBu^Ar_3_NC)]^2+^ clearly demonstrate that structural rigidity of the ligand environment can be used to manipulate the electronic structure of MC excited states and highlight that destabilizing MC excited states through increased structural rigidity is an important design criterion for extending CT excited state lifetimes.

**Figure 9:**
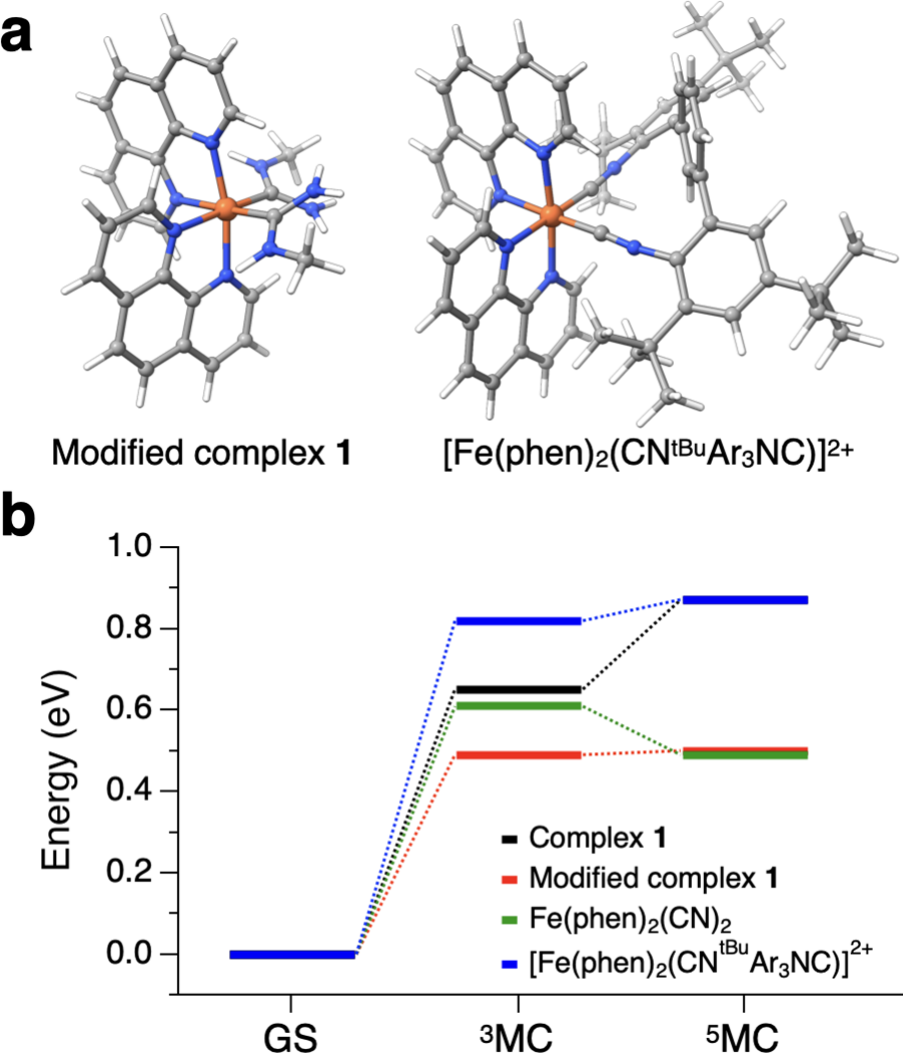
(a) B3LYP DFT optimized structures of the ground states of modified complex **1** and [Fe­(phen)_2_(CN^tBu^Ar_3_NC)]^2+^ (see SI Notes 7.3 and 7.4 for details of the B3LYP DFT results of these complexes). Fe is shown in orange, N in blue, C in gray, and H in white. (b) Relative energies of the ^3^MC and ^5^MC states with respect to the ground state (GS), based on the B3LYP DFT optimized structures (Table 1).

The insights provided by these computational studies now allow us to revisit the VT-TA measurements, specifically the Δ*S* ^‡^ value obtained from the Eyring analysis (Section 2.1). It was noted that complexes whose lowest-energy excited states are derived from the (t_2g_)^5^(e_g_)^1^ configuration typically exhibit Δ*S* ^‡^ values in the range of −3 to −4 cm^–1^ K^–1^. However, for complex **1**, which has now been definitively shown to have the ^3^MC state as its lowest-energy excited state, the Δ*S* ^‡^ value obtained (−2.0 ± 0.1 cm^–1^ K^–1^) is notably smaller in magnitude. A reduction in the magnitude of Δ*S* ^‡^ could arise from a dynamic equilibrium between the ^3^MC state and an energetically accessible ^3^MLCT state, in which case Δ*S* ^‡^ would reflect an average over these structurally distinct states; however, the time-resolved Kβ XES data clearly rule out this possibility. Instead, we propose that the smaller magnitude of Δ*S* ^‡^ directly reflects the steric constraints highlighted by the DFT calculations above. Recalling that the sign and magnitude of Δ*S* ^‡^ are dictated in large part by the degree of structural distortion associated with the rate-limiting step for ground state recovery, the restricted degrees of freedom, such as those identified by the DFT calculations, should lead to a smaller structural distortion in the thermalized ^3^MC excited state relative to the ground state. This would lead to a smaller overall volume change (and therefore a Δ*S* ^‡^ value that is smaller in magnitude but still negative) during ground state recovery consistent with experiment and simulation.

Additionally, for complex **1**, [Fe­(phen)_3_]^2+^, Fe­(phen)_2_(CN)_2_, and [Fe­(phen)­(CN)_4_]^2–^, the B3LYP DFT calculations show that the ^5^MC state energies are more widely distributed (0.23 to 0.96 eV) than the ^3^MC state energies (0.56 to 0.78 eV) (Figure 8b and Table 1). The optimized structures of the ^5^MC states for these Fe complexes show elongation of all Fe–ligand bonds (SI Note 7.2). In contrast, the optimized structures of the ^3^MC states reveal distinct distortion patterns: complex **1** and Fe­(phen)_2_(CN)_2_ exhibit predominantly axial elongation, whereas [Fe­(phen)_3_]^2+^ and [Fe­(phen)­(CN)_4_]^2–^ show clear equatorial elongation (SI Note 7.2). These would highlight that elongation of all Fe–ligand bonds in the ^5^MC state accentuates the influence of the strong field ligands in heteroleptic complexes while the pseudo-Jahn–Teller distortion in the ^3^MC state favors expansion along the moderate field Fe–pyridyl N bonds. Heinze and coworkers investigated *trans*- and *cis*-[Fe­(pdmi)_2_]^2+^ (H_2_pdmi = (2-pyridyl)­di­(3-methylimidazolium-1-yl)­methane), which feature two pyridine and four carbene ligands, using VT-TA spectroscopy.^72^ They found that, for the ^3^MLCT → ^3^MC transition, the *trans* isomer exhibits an unobservable activation barrier whereas the *cis* isomer shows a barrier of 492 cm^–1^. They compared this experimental finding to DFT calculations, which showed that the ^3^MC state energy of the *cis* isomer is higher than that of the *trans* isomer due to its coordination environment. Specifically, the *cis* isomer lacks the *trans*-N–Fe–N arrangement, which enables an easy Jahn–Teller distortion, but instead features only *trans*-N–Fe–C and *trans*-C–Fe–C arrangements. The similar influence of ligand arrangement in the ^3^MC state energy was computationally examined for cyclometalated Fe­(II) complexes by Dixon and coworkers,^81,82^ and was considered for Fe­(II) complexes bearing bidentate pyridyl-carbene ligands by Gros and coworkers.^67^

We also computationally explored the effect of ligand arrangement on the relative energies of the MC excited states. DFT calculations were performed on two [Fe­(py)_2_(CN)_4_]^2–^ (py = pyridine) isomers: *trans*-[Fe­(py)_2_(CN)_4_]^2–^, in which the two moderate field py ligands occupy the axial positions, and *cis*-[Fe­(py)_2_(CN)_4_]^2–^, where the two py ligands neighbor each other (Figures 10a and S18; see SI Note 7.5 for details of the B3LYP DFT results of these complexes). These calculations show that, while the ^5^MC state energies are similar for both isomers (0.82 and 0.84 eV for the *trans* and *cis* isomers, respectively), the ^3^MC state energy is significantly lower for the *trans* isomer (0.44 vs 0.74 eV) (Figure 10b and Table 1). In the optimized structures of the ^3^MC states, the *trans* isomer shows axial elongation along the two moderate field Fe–py N bonds, whereas the *cis* isomer displays equatorial elongation involving both the two moderate field Fe–py N bonds and the two strong field Fe–cyanide C bonds (SI Note 7.5). These results again underscore the importance of ligand arrangement in modulating the energetics of ^3^MC excited states. Specifically, when designing the ligand environment to destabilize MC excited states, strong field ligands need to be properly arranged such that the pseudo-Jahn–Teller distortion in the ^3^MC state involves Fe–strong field ligand bonds.

**Figure 10:**
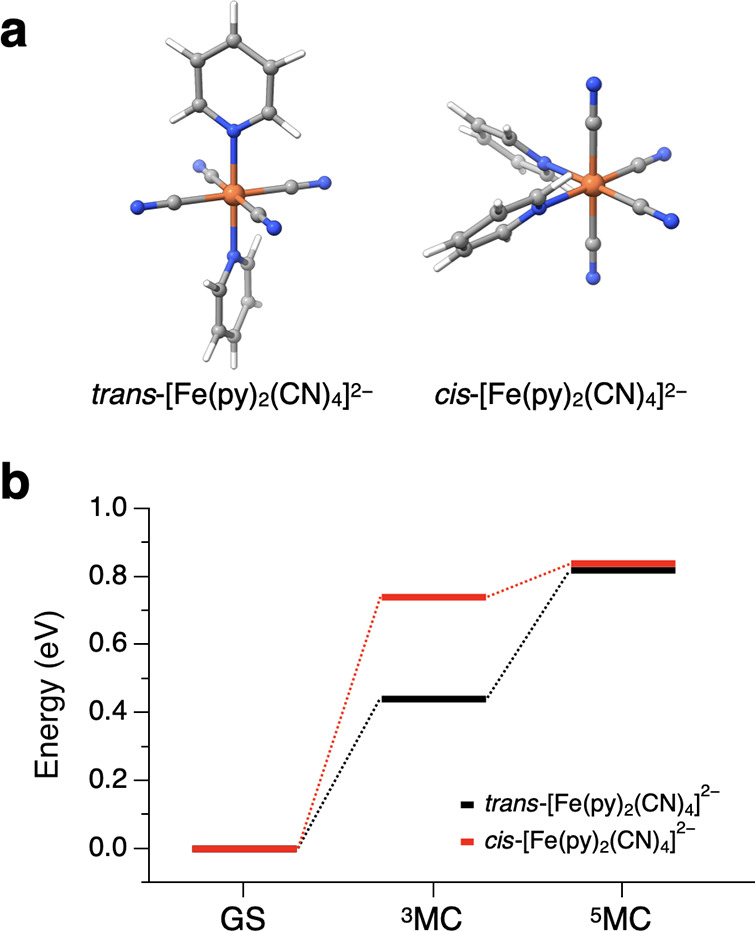
(a) B3LYP DFT optimized structures of the ground states of *trans*- and *cis*-[Fe­(py)_2_(CN)_4_]^2–^ (see SI Note 7.5 for details of the B3LYP DFT results of these complexes). Fe is shown in orange, N in blue, C in gray, and H in white. (b) Relative energies of the ^3^MC and ^5^MC states with respect to the ground state (GS), based on the B3LYP DFT optimized structures (Table 1).

## 4 Conclusion

Time-resolved Fe Kβ XES (utilizing both the VtC and Kβ mainline spectral regions), complemented by VT-TA spectroscopy, clearly elucidates the excited state structure and relaxation dynamics of complex **1**. The dominant relaxation pathways involve the rapid decay of the initial ^1,3^MLCT excited state to two dynamically distinct ^3^MC excited states (likely two zero-field split components of the ^3^T_1_ state), each exhibiting a different lifetime for ground state recovery. This work complements and expands upon the previous room-temperature optical TA spectroscopy study by Paulus et al.,^19^ leading to a definitive assignment of the long-lived excited state of complex **1** as a ^3^MC state. Generation of the ^5^MC excited state from the optically excited ^1,3^MLCT state also appears to provide a relaxation channel for complex **1**; however, it represents only a minor contribution to the overall excited state dynamics. The present study highlights the capability of time-resolved Kβ XES to characterize the role of MC excited states in the relaxation mechanisms of transition metal complexes, as well as the value of the synergistic use of multiple techniques to obtain detailed insight into their excited state dynamics.

Comparison of the excited state relaxation mechanism of complex **1** with those of the Fe polypyridyl cyanide complexes, supported by the DFT calculations, reveals that structural rigidity and ligand arrangement play crucial roles in controlling photophysical and photochemical properties. Specifically, increased structural rigidity can raise MC excited state energies by hindering optimization of metal–ligand bond lengths and bond angles in MC states, and the relative coordination positions of moderate/weak field ligands in heteroleptic complexes can influence ^3^MC excited state energies through distinct pseudo-Jahn–Teller distortion patterns. These results further highlight the importance of rational ligand design, through control of structural rigidity and ligand arrangement, for tuning photophysical and photochemical properties of transition metal complexes.

## Supplementary Material



## Notes

Methods; supporting data and analysis of time-resolved Kβ XES and DFT calculations (PDF)

## #

RIKEN SPring-8 Center, Sayo, Hyogo 679-5148, Japan
