# Supplementary material for: Pursuing Heteroleptic Ligand Design Principles for Photoactive Fe Complexes with Ultrafast X‑ray Emission and Variable-Temperature Optical Spectroscopies
Source: J Am Chem Soc. 2026 Jul 28;148(31):33607–21. doi: 10.1021/jacs.6c09850 (PMC13474941; doi:10.1021/jacs.6c09850)
Supplement: Supplementary file 1 [file ja6c09850_si_001.pdf]

Supporting Information

Pursuing Heteroleptic Ligand Design Principles for Photoactive Fe Complexes with Ultrafast X-ray Emission and Variable-Temperature Optical Spectroscopies

Hyeongtaek Lim,*[,1,†] Karl C. Nielsen,[2] Natalia E. Powers-Riggs,[1] Sumana L. Raj,[1,3] Roberto Alonso-Mori,[3] Leland B. Gee,[3] Tim B. van Driel,[3] Patrick L. Kramer,[3] Matthieu Chollet,[3] Amy A. Cordones,[1] James K. McCusker,*[,2] and Kelly J. Gaffney*[,1]

[1]Stanford PULSE Institute, SLAC National Accelerator Laboratory, Stanford University, Menlo Park, California 94025, United States
[2]Department of Chemistry, Michigan State University, East Lansing, Michigan 48824, United States
[3]Linac Coherent Light Source, SLAC National Accelerator Laboratory, Stanford University, Menlo Park, California 94025, United States

*Corresponding Authors:
Hyeongtaek Lim – Email: limh@spring8.or.jp
James K. McCusker – Email: jkm@chemistry.msu.edu
Kelly J. Gaffney – Email: kgaffney@slac.stanford.edu

Present Address:
†Hyeongtaek Lim – RIKEN SPring-8 Center, Sayo, Hyogo 679-5148, Japan

Table of Contents

SI Note 1: Methods S3

SI Note 2: DFT Calculations of Complex **1** S7

SI Note 3: Global Analysis of the Transient VtC XES Difference Data in the −0.2 to 0.4 ps Delay Time Range S13

SI Note 4: Analysis of the Transient Kβ Mainline XES Difference Data in the −0.2 to 0.4 ps Delay Time Range S15

SI Note 5: Analysis of the Transient Kβ Mainline XES Difference Data in the −1.0 to 9.5 ps Delay Time Range S18

SI Note 6: Dependence of the Transient Kβ Mainline XES Difference Data on Laser Pulse Energy S21

SI Note 7: Additional DFT Calculations of the Fe Complexes S23

SI Note 8: Cartesian Coordinates of the DFT Optimized Structures S34

References S65

SI Note 1: Methods

1.1. Sample preparation

$[Fe(phen)_2(C_4H_{10}N_4)]^{2+}$ (**1**; phen = 1,10-phenanthroline) was prepared as previously described.[1] For spectroscopic measurements, solution samples of complex **1** in methanol were prepared.

1.2. Variable-temperature transient optical absorption spectroscopy

Variable-temperature transient optical absorption (VT-TA) spectroscopy was performed using a setup described previously.[2] For these measurements, the solution sample of complex **1** in spectrophotometric grade methanol was loaded into a 1 mm pathlength cryostat cuvette, and the concentration was adjusted to give an absorbance of 0.65 at the 500 nm excitation wavelength. The sample was placed in an optical dewar and cooled to 235 K. VT-TA data were acquired as a function of temperature in 10 K increments from 235 to 295 K, with a 15 min equilibration period at each temperature prior to measurement. The data were collected at 580 and 680 nm, corresponding to the ground state bleach and excited state absorption, respectively. These probe wavelengths were selected based on our previously reported room-temperature time-resolved broadband optical absorption data of complex **1**,[1] which show the ground state bleach at wavelengths shorter than ~625 nm and the excited state absorption at longer wavelengths, with the largest signal magnitudes at 580 and 680 nm, respectively. These spectral features exhibit minimal spectral shifting over the relevant time range at room temperature, supporting the use of these wavelengths to track the VT-TA kinetics. The kinetic traces were found to be independent of probe wavelength, and therefore only the data acquired at 580 nm are presented in this study. The kinetic traces were fit with a biexponential function in Igor Pro. The data obtained at 580 nm were fit to a linearized Eyring model to extract thermochemical parameters. For the Eyring analysis, only the 235–285 K data were included in the fit, as the 295 K data point deviated markedly from the linear trend. This deviation is most likely attributable to an experimental artifact rather than to the intrinsic temperature dependence. One possible origin of this deviation is the release of trace accumulated moisture into the vapor phase as the sample is warmed from low to high temperature. The kinetics of complex **1** are likely sensitive to the solvent environment, potentially in part because the protonated carbene backbone can participate in hydrogen bonding. The sensitivity of complex **1** to its solvent environment is the subject of ongoing investigation. Importantly, we note that the Eyring analysis based on the 235–285 K data is sufficient for the purposes of the present study.

1.3. Time-resolved Fe Kβ X-ray emission spectroscopy

Time-resolved Fe Kβ X-ray emission spectroscopy (XES) was performed at the X-ray Correlation Spectroscopy (XCS) instrument at the Linac Coherent Light Source (LCLS).[3,4] The solution sample of complex **1** in methanol (~15 mM) was delivered using a 50 μm diameter cylindrical

liquid jet using an HPLC pump to recirculate the sample. The jet was enclosed in a chamber with Kapton tape windows, and He was continuously flowed inside the chamber. Optical excitation of the sample was achieved using laser pulses centered at 520 nm, generated from an optical parametric amplifier pumped by the 800 nm fundamental of a Ti:sapphire laser system, in a nearly colinear geometry with respect to the X-ray probe. The optical pump pulses had a 120 Hz repetition rate, 2.2 μJ pulse energy, ~50 fs pulse duration, and ~80 μm diameter spot size at the sample position. The pulse energy of 2.2 μJ was selected based on the linear regime observed in a laser pulse energy titration scan (Figure S12) and is not expected to cause multiphoton absorption effects. The excitation fraction is estimated to be 65% based on the analysis of the transient Kβ XES data (see SI Note 4). The X-ray probe pulses were generated by self-amplified spontaneous emission (SASE) operation with a 120 Hz repetition rate and ~40 fs pulse duration. The X-ray pulses were centered at 8 keV in pink mode and focused with Be compound refractive lenses. The incident X-ray intensity (I_0) was measured using intensity position monitors. A multicrystal analyzer spectrometer employing four cylindrically bent Ge(620) crystals arranged in the von Hamos geometry was used to analyze Fe Kβ emission energies,[5] and the analyzed emission signals were measured using an ePix100 detector.[6,7] The emitted beam path was enclosed by a He-filled bag to minimize signal attenuation. Scattering signals were simultaneously measured with an ePix10k 2M detector,[6,8] and these were used as initial normalization factors for the emission signals due to an advantage of monitoring fluctuations in the liquid jet in addition to I_0 fluctuations. The time tool was used to account for the inherent jitter in the arrival time between the optical pump and X-ray probe pulses.[9]

Two-dimensional transient Kβ XES data were collected by continuously scanning the delay stage over the defined delay time range. Every two of every seven X-ray shots were taken without the optical pump, and these were used to construct the laser-off spectra representing the electronic ground state. A single date-collection run generally included ~40,000 or ~70,000 X-ray shots depending on the defined delay time range. For each run, the data were filtered to remove “bad” shots (e.g., outliers in the I_0 and scattering signals, time tool parameters, and I_0–scattering signal correlations), such that ~10% of the shots were filtered out of the data. The filtered data were then binned with respect to the delay time and normalized by setting the area under the Kβ mainline region (7037–7072 eV) to 100. These filtered, binned, and normalized data were averaged across multiple runs, weighted by the number of shots. The emission energy was calibrated by matching the laser-off Kβ mainline XES spectrum to a singlet state reference spectrum from $[Fe(bpy)_3]^{2+}$ (bpy = 2,2′-bipyridine).[10]

For the valence-to-core (VtC) XES data (Figure 4 and SI Notes 2 and 3) and the transient Kβ mainline XES difference spectrum averaged over the 20 to 50 ps delay time range (Figure 6), three-point moving average smoothing along the energy axis was applied to improve the signal-to-noise ratio. While these smoothed data were used for the analysis presented in this study, we note that the analysis of the unsmoothed data yields equivalent results.

1.4. Global analysis

Global analysis of the transient Kβ XES data was performed using an in-house written Python script that utilized the LMFIT package (version 1.3.2).[11] Details of this global analysis script have been described elsewhere.[12] In the present study, the least-squares minimization in the global analysis employed χ^2 that includes a weighting factor corresponding to the data uncertainty, which was estimated as the standard deviation from the data averaging. We note that time zero (t_0) of the transient Kβ XES data presented in this study is based on the results of the global analysis.

1.5. Calculations

Density functional theory (DFT) calculations were performed with ORCA (version 5.0.3).[13,14] For complex **1**, geometry optimizations were conducted using the def2-TZVP basis set[15] and the def2/J auxiliary basis set[16] on all atoms, Grimme's DFT-D3 dispersion correction with Becke–Johnson damping (D3BJ),[17,18] and the *TightSCF* and *SlowConv* convergence criteria. The conductor-like polarizable continuum model (CPCM)[19] was used to account for solvent effects (methanol). To examine the influence of the functional, geometry optimizations were performed with various functionals, including the generalized gradient approximation (GGA) (BP86[20,21] and PBE[22]), hybrid (B3LYP[23,24] and PBE0[25,26]), meta-GGA (TPSS[27]), and hybrid meta-GGA (TPSSh[28]) functionals. The ground state structure of complex **1** was optimized using the crystal structure as the starting model.[1] The quintet metal-centered (^{5}MC) state structure was optimized starting from the optimized ground state structure by specifying the appropriate spin multiplicity. For the metal-to-ligand charge transfer (MLCT) state, only the triplet MLCT (^{3}MLCT) state was considered in our DFT calculations. The ^{3}MLCT and triplet metal-centered (^{3}MC) states have the same spin multiplicity. Considering that the ^{3}MC state structure is expected to be closer to the ^{5}MC state structure than to the ground state structure, the ^{3}MC state structure was optimized using the optimized ^{5}MC state structure as the starting model by specifying the appropriate spin multiplicity. In contrast, the ^{3}MLCT state structure, which is expected to be closer to the ground state structure, was optimized starting from the optimized ground state structure by specifying the appropriate spin multiplicity. The assignments of the states for the optimized structures were made based on the spin population on Fe. At the level of theory used in these calculations, among the tested functionals, only the BP86 functional enabled the geometry optimization of the ^{3}MLCT state; other functionals led to the same optimized ^{3}MC state structure for the triplet spin multiplicity, regardless of the starting structure. Therefore, the results from the BP86 calculations were used for the analysis of the VtC XES data of complex **1**. The optimized ground state structure and the molecular orbitals (MOs) of complex **1** were visualized using UCSF ChimeraX (versions 1.8 and 1.9)[29] and its SEQCROW bundle (version 1.8.16).[30,31]

VtC XES spectra of complex **1** were calculated for the BP86 optimized structures using the BP86 functional and the same level of theory as employed in the geometry optimizations, except that the CP(PPP) basis set[32] was used for Fe. Spin–orbit coupling was included, and thus the spin–orbit coupling corrected VtC XES calculation results are presented in this study. The

simulated VtC XES spectra were generated by broadening the calculated transitions with a Gaussian function having a 3.5 eV full width at half maximum (FWHM) using the orca_mapspc program, which is part of the ORCA package. For facile comparison with the experimental data, the simulated VtC XES spectra were shifted by +182.15 eV to match the simulated and experimental $K\beta_{2,5}$ features of the ground state. We note that, for reference, VtC XES spectra were also calculated using the B3LYP, TPSS, and TPSSh functionals with their respective optimized structures. The calculated spectral changes for the ^{3}MC and ^{5}MC states relative to the ground state spectrum in these B3LYP, TPSS, and TPSSh calculations were similar to those in the BP86 calculations. Thus, although the VtC XES spectrum for the ^{3}MLCT state was calculated only with the BP86 functional (as it was the only functional that allowed for the geometry optimization of the ^{3}MLCT state), we consider that other functionals would generate ^{3}MLCT state spectra equivalent to that from the BP86 functional, relative to their respective ground state spectra (if the ^{3}MLCT state structures were optimized for these other functionals).

Additional DFT calculations were conducted for $[Fe(phen)_3]^{2+}$, $Fe(phen)_2(CN)_2$, $[Fe(phen)(CN)_4]^{2-}$, modified complex **1**, $[Fe(phen)_2(CN^{tBu}Ar_3NC)]^{2+}$, *trans*-$[Fe(py)_2(CN)_4]^{2-}$, and *cis*-$[Fe(py)_2(CN)_4]^{2-}$ (py = pyridine) (SI Note 7). For these Fe complexes, geometry optimizations targeting the ground, ^{3}MLCT, ^{3}MC, and ^{5}MC states were performed as described above for complex **1**. For $[Fe(phen)_3]^{2+}$, $Fe(phen)_2(CN)_2$, and $[Fe(phen)(CN)_4]^{2-}$ (including complex **1**), a benchmark study of functionals was conducted to compare the BP86, B3LYP, and PBE0 results, which suggested that the B3LYP functional is appropriate for comparing the excited state energetics (SI Note 7.1). Accordingly, the B3LYP functional was used for the other complexes. For $[Fe(phen)_3]^{2+}$, $Fe(phen)_2(CN)_2$, and $[Fe(phen)(CN)_4]^{2-}$, their respective crystal structures were used as the starting models for the geometry optimizations of the ground states (note that the crystal structure used for $[Fe(phen)(CN)_4]^{2-}$ corresponds to the Fe(III) complex).[33–35] The starting models for modified complex **1**, $[Fe(phen)_2(CN^{tBu}Ar_3NC)]^{2+}$, and the two isomers of $[Fe(py)_2(CN)_4]^{2-}$ were generated using Avogadro (version 1.2.0).[36] Specifically, the starting model for modified complex **1** was created from the B3LYP optimized ground state structure of complex **1** by breaking the N–N bond in the bidentate carbene ligand and appropriately attaching H atoms. The starting model for $[Fe(phen)_2(CN^{tBu}Ar_3NC)]^{2+}$ was generated by replacing the two monodentate cyanide ligands in the B3LYP optimized ground state structure of $Fe(phen)_2(CN)_2$ with a bidentate diisocyanide ligand.[37] The starting models for the two isomers of $[Fe(py)_2(CN)_4]^{2-}$ were prepared by modifying the optimized ground state structure of $[Fe(py)_6]^{2+}$, which was obtained from its crystal structure.[38] We note that, similar to complex **1**, the optimized structure for the ^{3}MLCT state was obtained in only a few cases (including the BP86, B3LYP, and PBE0 calculations for $[Fe(phen)(CN)_4]^{2-}$). Thus, in this study, only the ^{3}MC and ^{5}MC states are considered for these Fe complexes. The optimized structures of the ground states of these Fe complexes were also visualized using UCSF ChimeraX (versions 1.8 and 1.9).[29]

SI Note 2: DFT Calculations of Complex 1

2.1. Geometric and electronic structures

In SI Note 2, the BP86 DFT calculation results of complex **1** are presented. In the electronic ground state, complex **1** is a low-spin pseudo-octahedral Fe(II) complex. The optimized structure of the ground state of complex **1** is shown in Figure S1 and is broadly similar to those of the ^{3}MLCT, ^{3}MC, and ^{5}MC states. The energies, Fe–ligand bond lengths, and spin populations on Fe of the optimized structures are summarized in Table S1. Note that the spin population on Fe for each state (i.e., 0, ~1, ~2, and ~4 for the ground, ^{3}MLCT, ^{3}MC, and ^{5}MC states, respectively) confirms that the optimized structures obtained correspond to the intended states. The Fe 3d-based MOs and the MOs related to the MLCT are shown in Figures S2–S5. For simplicity, although the Fe center in complex **1** does not exhibit a perfect octahedral geometry, octahedral symmetry labels are used here.

The optimized structure of the ground state agrees well with the crystal structure[1] within 0.02 Å in the Fe–ligand bond lengths (Table S1). Figure S2 shows the selected MOs for the ground state. MO135, MO136, and MO137 are the occupied orbitals corresponding to the t_{2g} set of the Fe 3d orbitals, while MO144 and MO148 are the unoccupied orbitals corresponding to the e_g set. MO138 is the lowest unoccupied molecular orbital (LUMO), which is ligand-based with predominant phen character.

Relative to the ground state, the ^{3}MLCT state does not exhibit significant changes in the bond lengths (only 0.02 Å increase in the average Fe–ligand bond length; Table S1). Formation of the ^{3}MLCT state from the ground state involves the transfer of an electron from the t_{2g} orbital to the ligand-based orbital as well as the spin moment change. In the ^{3}MLCT state calculated in this study, the MLCT corresponds to the MO137 → MO138 transition from the ground state (i.e., highest occupied molecular orbital (HOMO) to LUMO transition; Figure S2). As a result, in the ^{3}MLCT state, MO138α (the phen-based orbital) is occupied and MO137β (one of the t_{2g} orbitals) is unoccupied (Figure S3).

Formation of the ^{3}MC state from the ground state requires the promotion of an electron from the t_{2g} orbital to the e_g orbital as well as the spin moment change. The e_g orbitals possess the σ* antibonding Fe–ligand character. A single electron in the e_g orbital leads to the tetragonal Jahn–Teller distortion, which splits the e_g orbitals into the a_{1g} and b_{1g} orbitals and the t_{2g} orbitals into the e_g and b_{2g} orbitals, and causes Fe–ligand bond elongation. In the ^{3}MC state calculated in this study, MO138α which is the Fe $3d_{z2}$-based orbital (e_g in the octahedral symmetry and a_{1g} after the tetragonal Jahn–Teller distortion) is occupied (Figure S4), leading to Fe–ligand$_{axial}$ bond elongation. This is evidenced by the optimized ^{3}MC state structure, in which the bond lengths for the axial ligands (N1 and N4) are significantly increased (0.25 Å increase in the average Fe–ligand$_{axial}$ bond length; Table S1). In contrast, the bond lengths for the equatorial ligands (C1, C2, N2, and N3) show only minor changes (0.04 Å increase in the average Fe–ligand$_{equatorial}$ bond length; Table S1).

For the ^{5}MC state, two electrons should be promoted from the t_{2g} orbitals to the e_g orbitals, and their spin moments also need to be changed. This results in symmetric elongation of all Fe–ligand bonds. In the optimized ^{5}MC state structure, the bond lengths for both the axial and equatorial ligands are significantly increased (0.22 Å increase in the average Fe–ligand bond length; Table S1). Note that, as shown in Figure S5, all Fe 3d-based orbitals are occupied in the α manifold (MO130α, MO131α, MO136α, MO138α, and MO139α), while one t_{2g} orbital is occupied in the β manifold (MO135β).

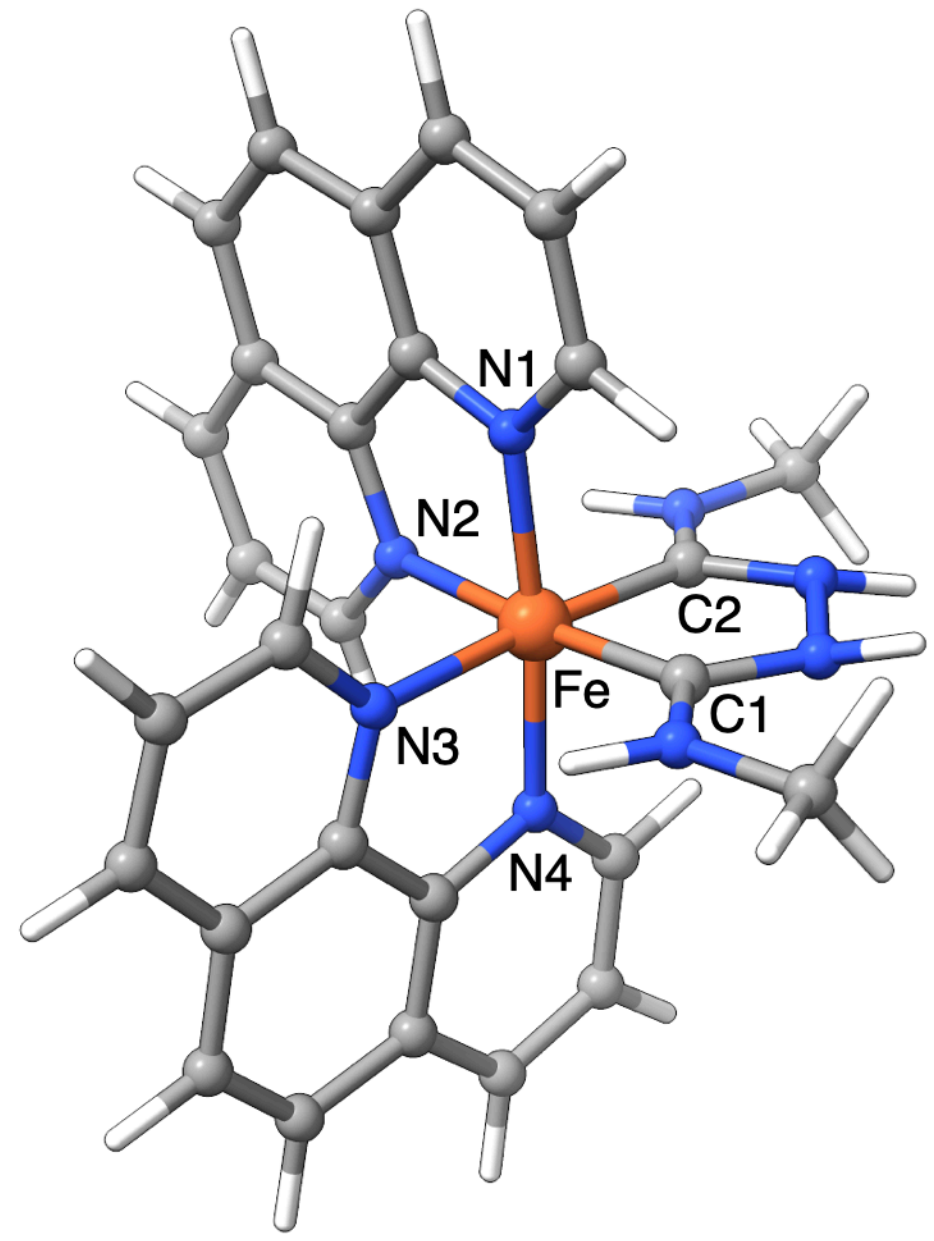

Figure S1. BP86 DFT optimized structure of the ground state of complex **1**. Fe is shown in orange, N in blue, C in gray, and H in white.

Table S1. Energies (eV), Fe–ligand bond lengths (Å), and spin populations on Fe for the BP86 DFT optimized structures of the ground, ^{3}MLCT, ^{3}MC, and ^{5}MC states of complex **1**. Bond lengths from the crystal structure are included for comparison. Numbering for C and N atoms corresponds to that in Figure S1.

	Crystal	DFT Ground state	DFT ^{3}MLCT state	DFT ^{3}MC state	DFT ^{5}MC state
<u>*Energy*</u>	-	0.00	1.60	1.46	2.32
<u>*Bond length*</u>					
Fe–C1	1.92	1.91	1.94	1.95	2.13
Fe–C2	1.92	1.91	1.94	1.95	2.13
Fe–N1	1.97	1.95	1.96	2.20	2.17
Fe–N2	2.01	2.00	2.03	2.03	2.21
Fe–N3	2.02	2.00	2.00	2.02	2.20
Fe–N4	1.97	1.95	1.96	2.20	2.17
<u>*Spin population on Fe*</u>					
Mulliken	-	0.00	1.20	2.13	3.76
Löwdin	-	0.00	1.07	1.97	3.58

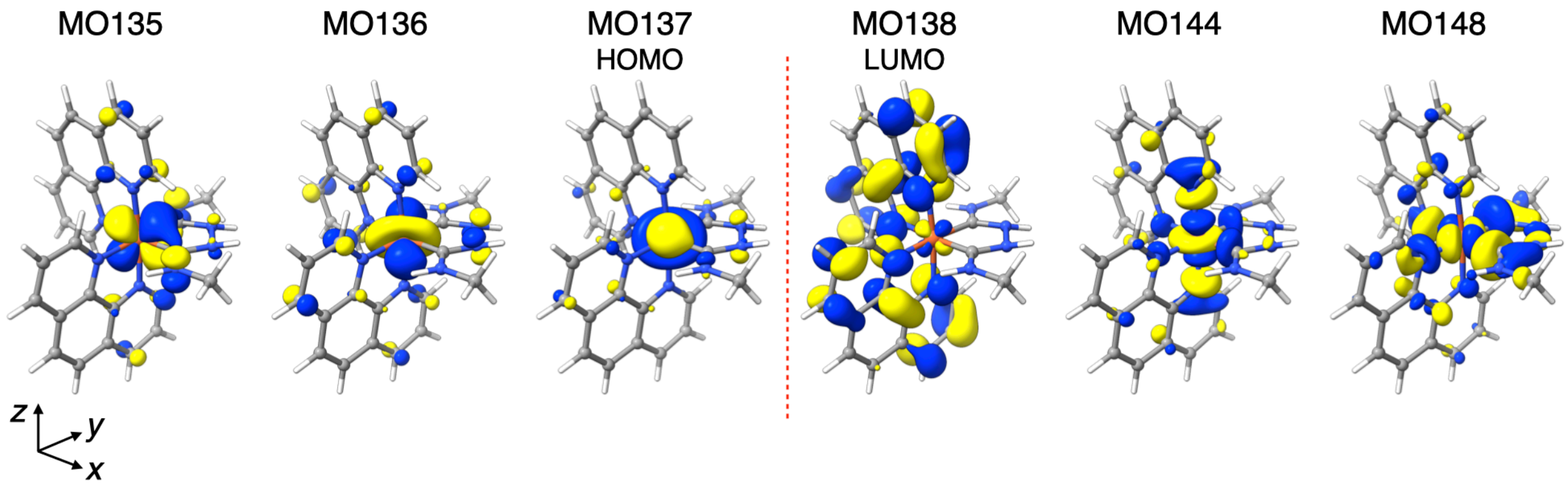

Figure S2. Selected MOs for the ground state of complex **1**. Occupied and unoccupied MOs are separated by a red dashed line. The molecular orientation is the same as that shown in Figure S1.

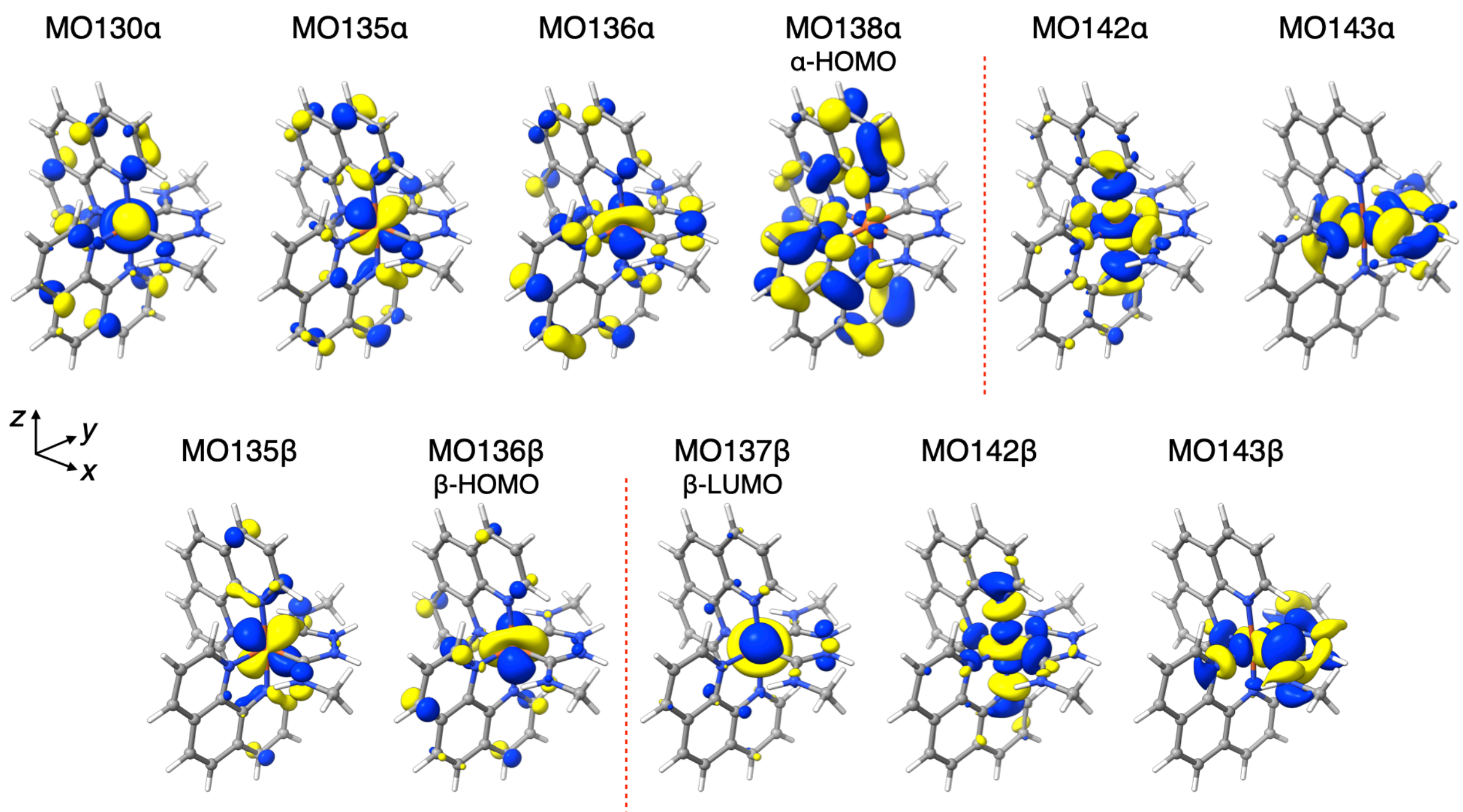

Figure S3. Selected MOs for the ^{3}MLCT state of complex **1**. Occupied and unoccupied MOs are separated by red dashed lines. The molecular orientation is similar to that shown in Figure S1.

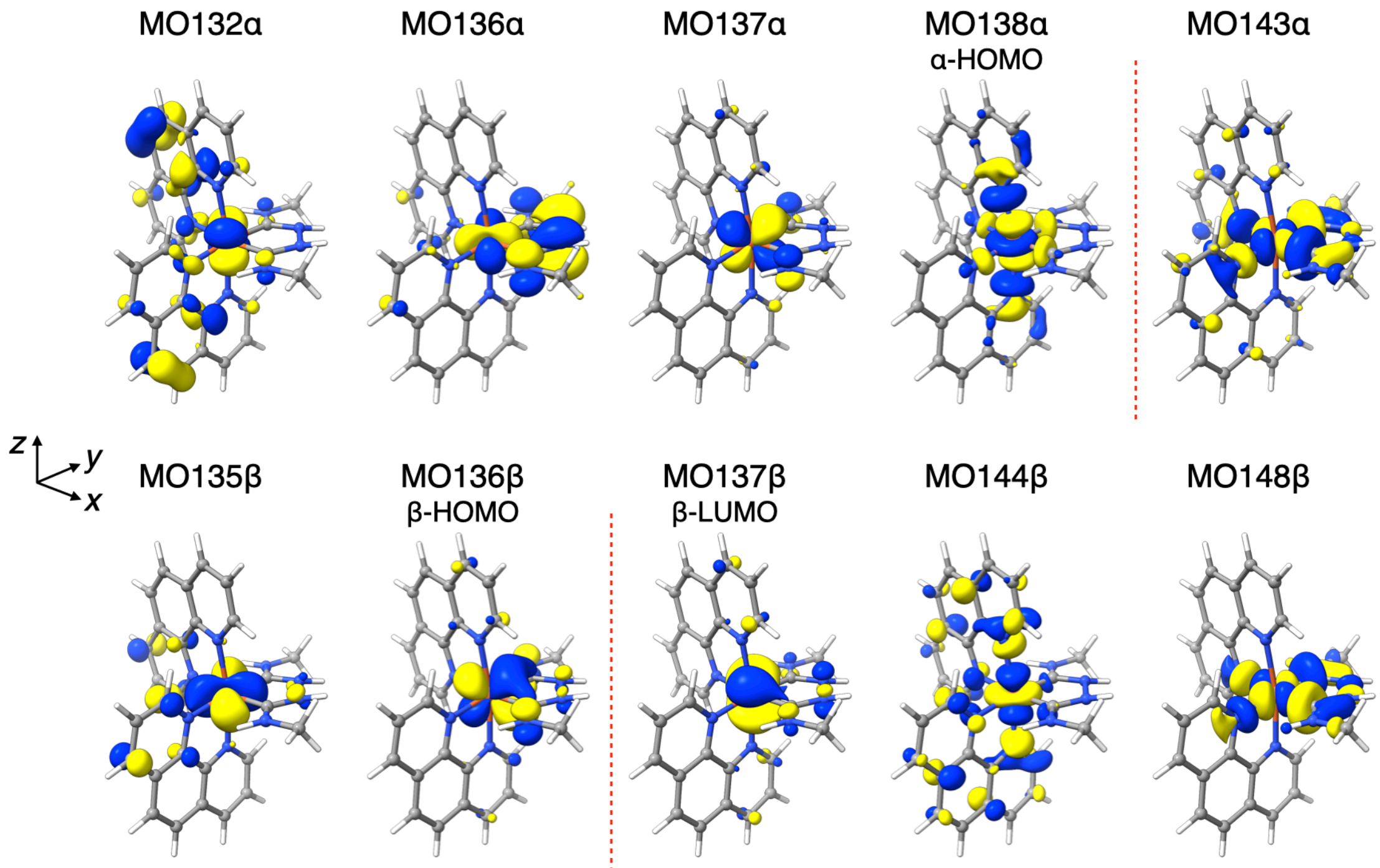

Figure S4. Selected MOs for the ³MC state of complex **1**. Occupied and unoccupied MOs are separated by red dashed lines. The molecular orientation is similar to that shown in Figure S1.

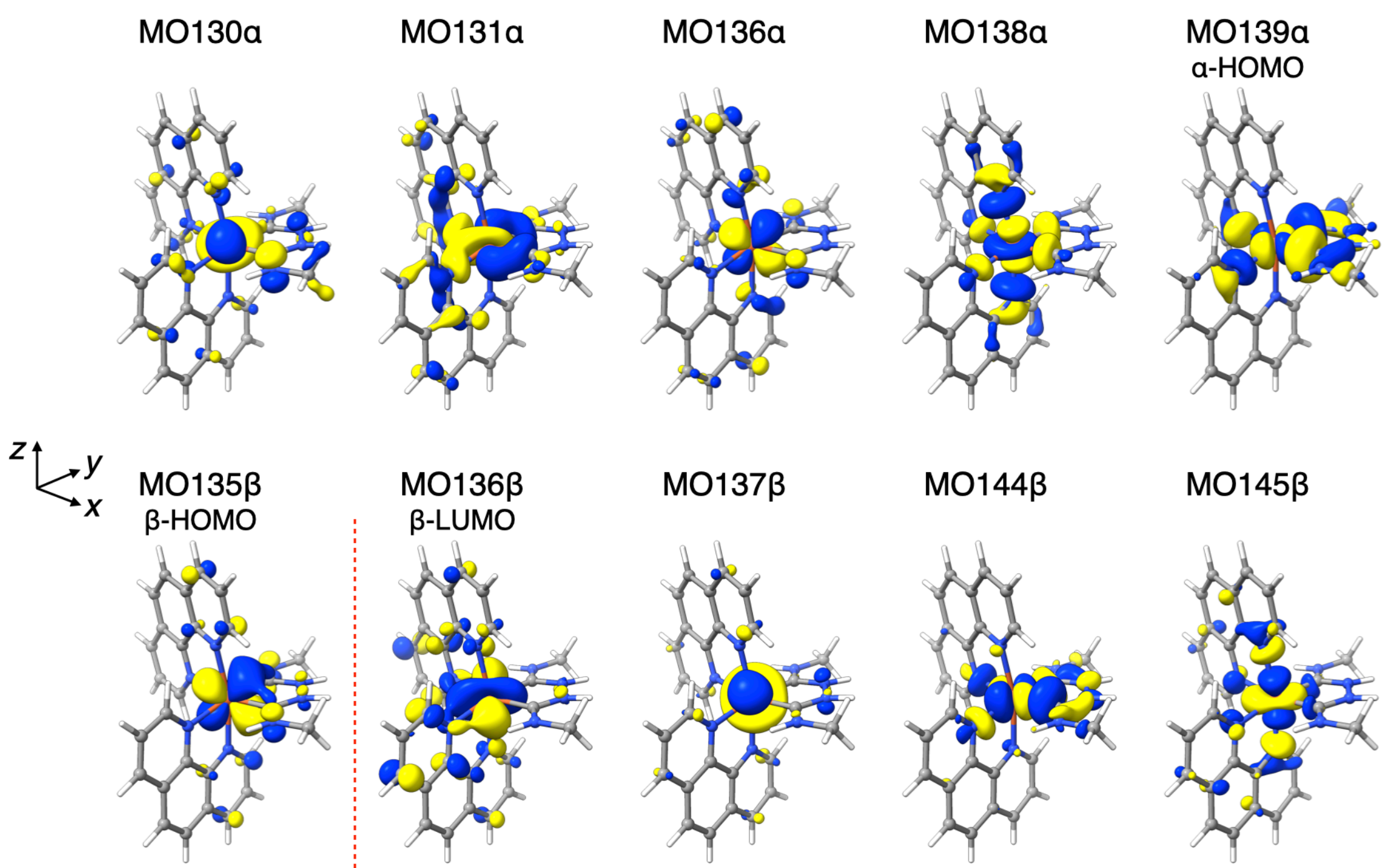

Figure S5. Selected MOs for the ⁵MC state of complex **1**. Occupied and unoccupied MOs are separated by a red dashed line. The molecular orientation is similar to that shown in Figure S1.

2.2. Calculations of VtC XES spectra

The BP86 DFT calculated VtC XES spectrum for the ground state of complex **1** is compared with the experimental laser-off spectrum in Figure S6, showing that the DFT calculation well reproduces the experimental data. The BP86 DFT calculated VtC XES spectra for the ground, ^{3}MLCT, ^{3}MC, and ^{5}MC states of complex **1** are shown in Figure S7a. The difference spectra, obtained by subtracting the ground state spectrum from the ^{3}MLCT, ^{3}MC, and ^{5}MC state spectra, are presented in Figure S7b.

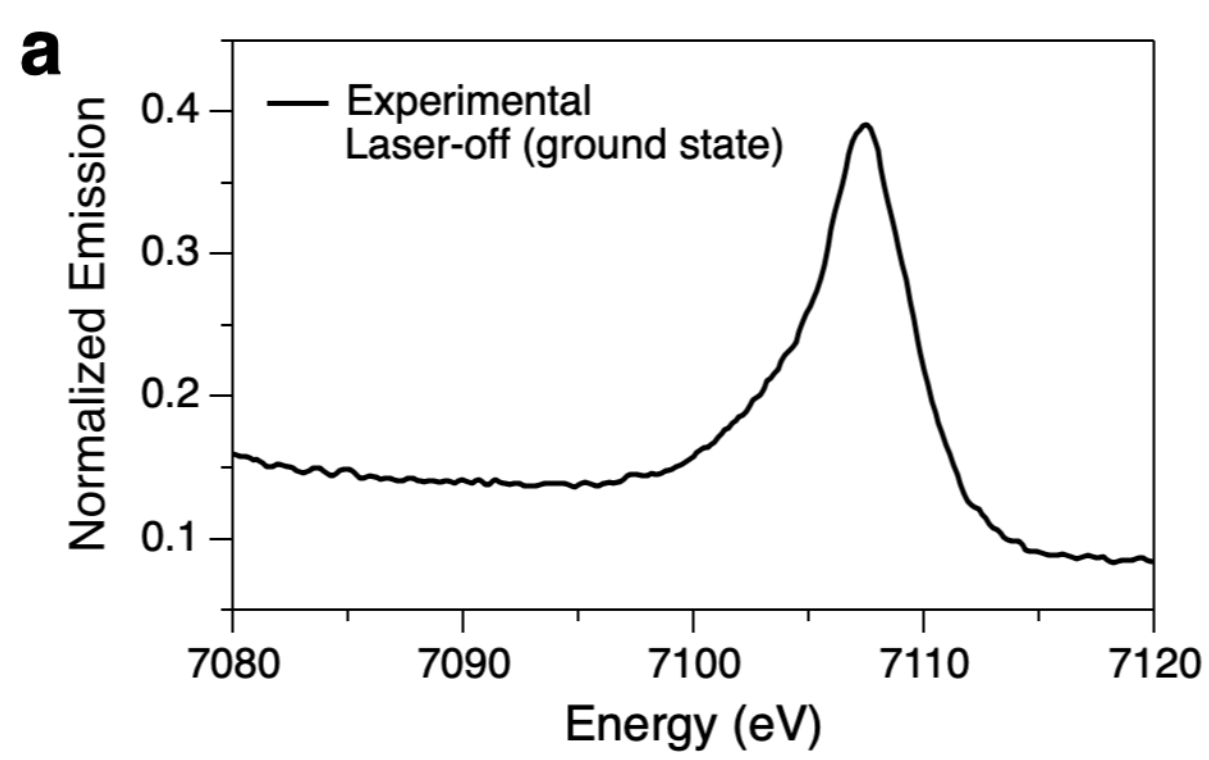

Figure S6. (a) Experimental and (b) BP86 DFT calculated VtC XES spectra of the ground state of complex **1**.

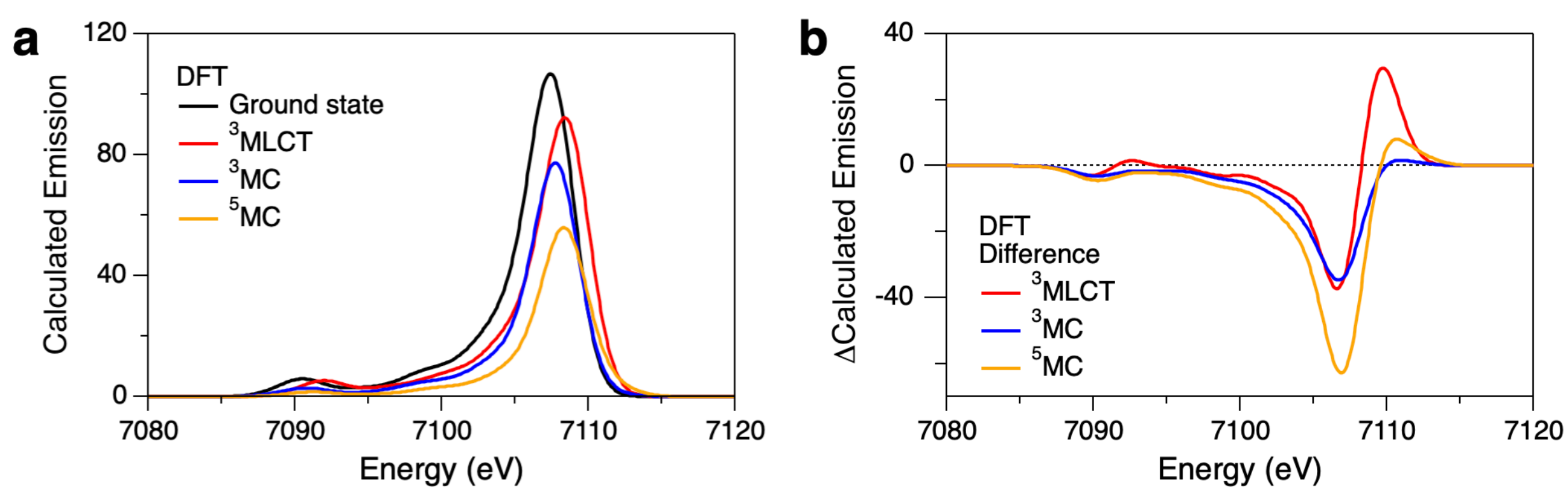

Figure S7. (a) BP86 DFT calculated VtC XES spectra of the ground, ^{3}MLCT, ^{3}MC, and ^{5}MC states of complex **1**. (b) BP86 DFT calculated VtC XES difference spectra for the ^{3}MLCT, ^{3}MC, and ^{5}MC states of complex **1**, obtained by subtracting the ground state spectrum from the respective spectra shown in (a).

SI Note 3: Global Analysis of the Transient VtC XES Difference Data in the −0.2 to 0.4 ps Delay Time Range

A global analysis of the transient VtC XES difference data of complex **1** in the −0.2 to 0.4 ps delay time range shown in Figure 4a was performed based on the excited state relaxation kinetic model developed in Section 2.3 of the main text (Figure 5e). Note that these transient difference data cover only up to 0.4 ps and therefore do not fully capture the biexponential decay profiles corresponding to the decays of the $^3MC_1$ and $^3MC_2$ excited states (see Sections 2.2.2 and 2.3 of the main text). Thus, a simplified kinetic model (^{1,3}MLCT → ^{3}MC → ground state) was considered for this global analysis. In this simplified kinetic model, the ^{3}MC excited state is considered to primarily represent the $^3MC_1$ excited state in the full kinetic model (Figure 5e). A system of differential rate equations can be constructed from this simplified kinetic model (eq S1) and solved to obtain the time-dependent excited state populations, which are used in the global analysis fitting.

$$\frac{\mathrm{d}[^{1,3}\mathrm{MLCT}]}{\mathrm{d}t} = -k_{\mathrm{MLCT}}[^{1,3}\mathrm{MLCT}] \tag{S1}$$
$$\frac{\mathrm{d}[^{3}\mathrm{MC}]}{\mathrm{d}t} = k_{\mathrm{MLCT}}[^{1,3}\mathrm{MLCT}] - k_{^3\mathrm{MC}}[^{3}\mathrm{MC}]$$

The initial conditions were [^{1,3}MLCT] = 1 and [^{3}MC] = 0 at $t = t_0$, implying that the appropriate excitation fraction (65%; see SI Note 4) should be applied when considering the populations from the fit.

In the fitting process, while the lifetimes ($\tau = 1/k$) of the ^{1,3}MLCT and ^{3}MC excited states were allowed to vary, the lower bound for τ_{MLCT} was set to the σ of the Gaussian instrument response function (IRF), which approximates the temporal resolution of the transient measurements. In addition, as the transient VtC XES difference signals are generally weak and the signals corresponding to the ^{1,3}MLCT excited state rapidly decay, the IRF FWHM and t_0 were fixed as obtained from the fit of the simultaneously measured transient Kβ mainline XES difference data shown in Figure 5a (see SI Note 4).

The measured data, global analysis fit, and residual are shown in Figure S8a, and the kinetic trace fits at 7107 and 7110 eV are presented in Figure 4d. These demonstrate that a good fit was obtained. The species associated difference spectra (SADS) and the time-dependent populations of the excited state species from this global analysis are shown in Figures 4e and S8b, respectively. The fit results are summarized in Table S2. We consider that the lifetime of the ^{3}MC excited state obtained from this global analysis (0.53 ps; Table S2) is reasonably close to that of the $^3MC_1$ excited state determined from the global analysis of the transient Kβ mainline XES difference data in the −1.0 to 9.5 ps delay time range shown in Figures S11a (left) and 5b (0.61 ps; see SI Note 5.2).

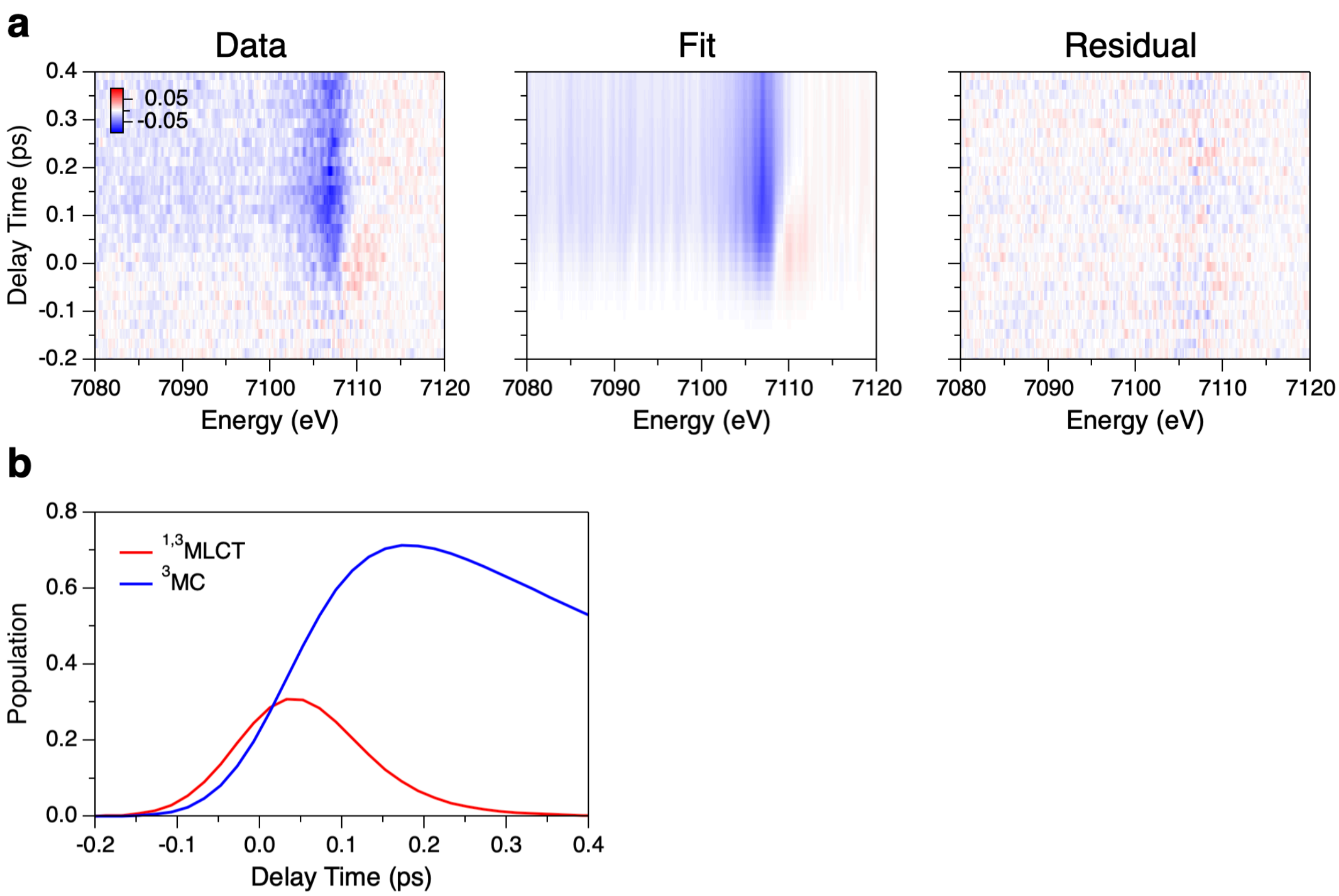

Figure S8. Time-resolved VtC XES data of complex **1** in methanol. (a) Measured transient VtC XES difference data (left), global analysis fit to the data (middle), and residual (right) in the −0.2 to 0.4 ps delay time range. (b) Time-dependent populations of the ^{1,3}MLCT and ^{3}MC excited states from the global analysis.

Table S2. Fit results from the global analysis of the transient VtC XES difference data of complex **1** in the −0.2 to 0.4 ps delay time range.[a]

τ_{MLCT} (fs)	60 (lower bound)
$\tau_{^3MC}$ (ps)	0.529 ± 0.039
IRF FWHM (ps)	0.145 (fixed)
R^2	0.735

[a]In the fitting process, the lower bound for τ_{MLCT} was set to the σ of the Gaussian IRF. The IRF FWHM and t_0 were fixed as obtained from the fit of the simultaneously measured transient Kβ mainline XES difference data (see SI Note 4). An uncertainty for $\tau_{^3MC}$ represents one standard deviation error that is calculated from the covariance matrix (in the fit using the LMFIT package).

SI Note 4: Analysis of the Transient Kβ Mainline XES Difference Data in the −0.2 to 0.4 ps Delay Time Range

The two-dimensional transient Kβ mainline XES difference spectra of complex **1** in the −0.2 to 0.4 ps delay time range, which were simultaneously measured with the transient VtC XES difference data (Figure 4a), are shown in Figures S9a (left) and 5a. The transient difference signals exhibit maximum intensity at ~0.1 ps and decay without pronounced spectral changes. The transient Kβ mainline XES difference spectrum at 0.13 ps is well fit with the triplet reference difference spectrum but not with the quintet spectrum (Figure S9b), indicating that the ^{3}MC excited state dominates the excited state dynamics in this delay time range.

As discussed in Section 2.2.2 of the main text, the short-lived ^{1,3}MLCT excited state is not resolved in these transient Kβ mainline XES difference spectra. Furthermore, as noted in SI Note 3, these transient difference data, covering only up to 0.4 ps, do not fully capture the biexponential decay profiles corresponding to the decays of the $^3MC_1$ and $^3MC_2$ excited states, in contrast to the transient difference data in the −1.0 to 9.5 ps delay time range shown in Figures S11a (left) and 5b. Thus, for a global analysis of these −0.2 to 0.4 ps transient difference data, we again employed a simplified kinetic model assuming a single exponential decay of the ^{3}MC excited state, which would primarily represent the $^3MC_1$ excited state in the full kinetic model (Figure 5e). In the global analysis fitting process, the lifetime of the ^{3}MC excited state ($\tau_{^3MC}$), IRF FWHM, and t_0 were allowed to vary.

The measured data, global analysis fit, and residual are shown in Figure S9a, and the kinetic trace fits at 7057.6 and 7060.1 eV are presented in Figure S9c. These demonstrate that a good fit was obtained. The SADS and the time-dependent population of the ^{3}MC excited state from this global analysis are shown in Figures S9d and S9e, respectively. The fit results are summarized in Table S3. The SADS of the ^{3}MC excited state is well fit with the triplet reference difference spectrum but not with the quintet spectrum (Figure S9d), confirming that the ^{3}MC excited state is the dominant excited state species in this delay time range. Based on this triplet reference fit, the excitation fraction is estimated to be 65% (Figure S9d). We consider that the lifetime of the ^{3}MC excited state obtained from this global analysis (0.73 ps; Table S3) is reasonably close to that of the $^3MC_1$ excited state determined from the global analysis of the transient Kβ mainline XES difference data in the −1.0 to 9.5 ps delay time range shown in Figures S11a (left) and 5b (0.61 ps; see SI Note 5.2).

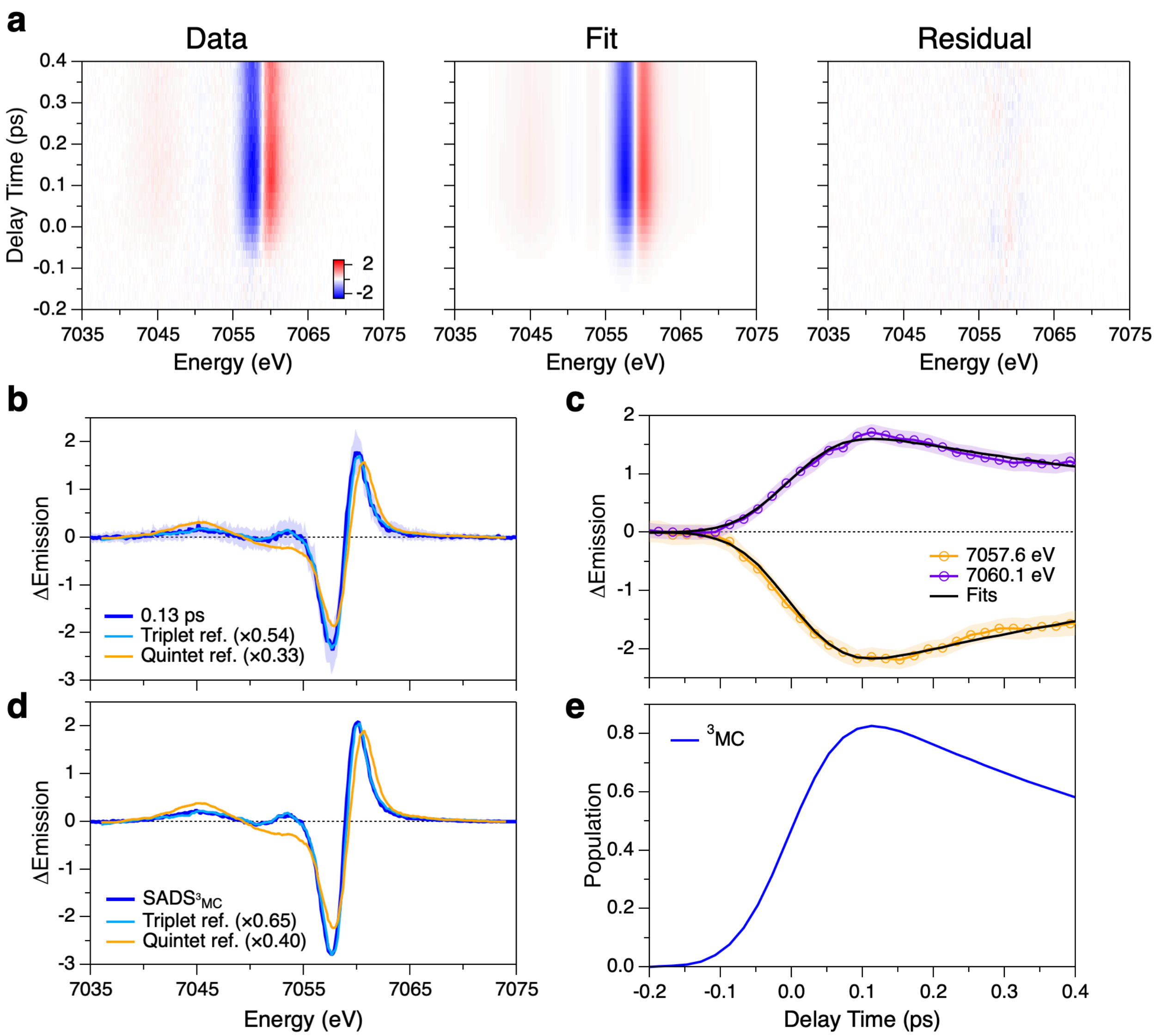

Figure S9. Time-resolved Kβ mainline XES data of complex **1** in methanol. (a) Measured transient Kβ mainline XES difference data (left), global analysis fit to the data (middle), and residual (right) in the −0.2 to 0.4 delay time range. (b) Transient Kβ mainline XES difference spectrum at 0.13 ps, and fits to this spectrum with the triplet and quintet reference difference spectra. (c) Kinetic traces at 7057.6 and 7060.1 eV (averaged over 1 eV range for each energy), and fits to these kinetic traces from the global analysis. Shaded areas in (b) and (c) represent the standard deviation from the data averaging. (d) SADS of the ^{3}MC excited state from the global analysis, and fits to this SADS with the triplet and quintet reference difference spectra. (e) Time-dependent population of the ^{3}MC excited state from the global analysis.

Table S3. Fit results from the global analysis of the transient Kβ mainline XES difference data of complex **1** in the −0.2 to 0.4 ps delay time range.[a]

$\tau_{^3MC}$ (ps)	0.733 ± 0.015
IRF FWHM (ps)	0.145 ± 0.002
R^2	0.986

[a]Uncertainties represent one standard deviation error on the fit parameters that is calculated from the covariance matrix (in the fit using the LMFIT package).

SI Note 5: Analysis of the Transient Kβ Mainline XES Difference Data in the −1.0 to 9.5 ps Delay Time Range

5.1. Fits to the transient Kβ mainline XES difference spectra

The transient Kβ mainline XES difference spectra of complex **1** averaged over the 0.05 to 0.35 ps and 6 to 8 ps delay time ranges are fit with the triplet and quintet reference difference spectra (Figure S10). This shows that the triplet reference difference spectrum, but not the quintet spectrum, well fits the transient difference spectra for both delay time ranges.

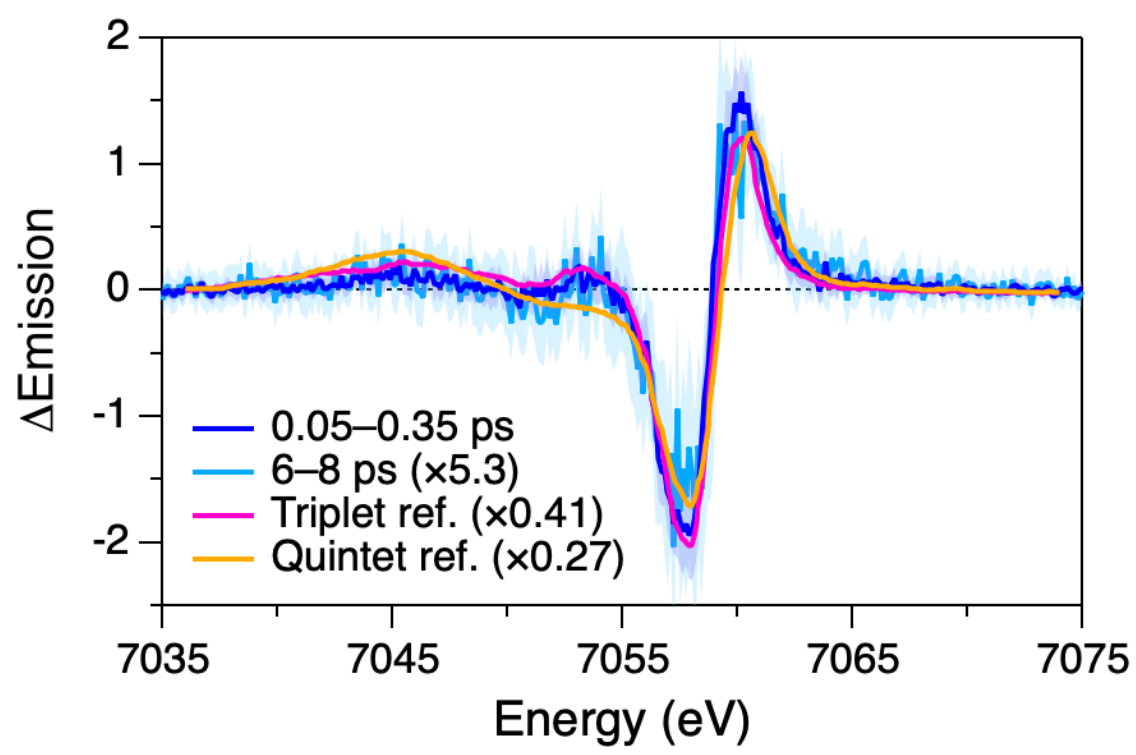

Figure S10. Transient Kβ mainline XES difference spectra of complex **1** in methanol averaged over the 0.05 to 0.35 ps and 6 to 8 ps delay time ranges, and fits to these spectra with the triplet and quintet reference difference spectra. The 6 to 8 ps averaged spectrum is multiplied by 5.3 to match the magnitude of the 0.05 to 0.35 ps averaged spectrum. Shaded areas represent the standard deviation from the data averaging.

5.2. Global analysis

A global analysis of the transient Kβ mainline XES difference data of complex **1** in the −1.0 to 9.5 ps delay time range, shown in Figures S11a (left) and 5b, was conducted based on the parallel decay kinetic model for the excited state relaxation dynamics developed in Section 2.3 of the main text (Figure 5e). As discussed in Section 2.2.2 of the main text, the short-lived ^{1,3}MLCT excited state is not resolved in these transient difference data and is therefore not considered in this global analysis. A system of differential rate equations for this kinetic model (eq S2) can be solved to obtain the time-dependent excited state populations, which are used in the global analysis fitting.

$$\frac{\mathrm{d}[^3\mathrm{MC}_1]}{\mathrm{d}t} = -k_{^3\mathrm{MC}_1}[^3\mathrm{MC}_1] \tag{S2}$$

$$\frac{\mathrm{d}[^3\mathrm{MC}_2]}{\mathrm{d}t} = -k_{^3\mathrm{MC}_2}[^3\mathrm{MC}_2]$$

The initial conditions were [$^3MC_1$] = c_1 and [$^3MC_2$] = $c_2 = 1 - c_1$ at $t = t_0$, where the ratio c_1:c_2 represents the branching ratio for the $^3MC_1$ and $^3MC_2$ excited states, which are assumed in this global analysis to fully form from the initial ^{1,3}MLCT excited state at $t = t_0$. Note that this again implies that the proper excitation fraction (65%; see SI Note 4) should be applied when considering the populations from the fit. In this global analysis, the $^3MC_1$ excited state is considered to correspond to the fast decay component.

With this kinetic model, an initial global analysis fit was performed by allowing all fit parameters (the lifetimes of the $^3MC_1$ and $^3MC_2$ excited states, c_1, IRF FWHM, and t_0) to vary (Fit 1). In Fit 1, the c_1 fit value was found to significantly depend on the initial parameters, while consistent fit values were obtained for the other parameters. Thus, several fits were conducted with c_1 fixed to systematically varied values while allowing the other parameters to vary (Fit 2). These yielded consistent fit values for the lifetimes, IRF FWHM, and t_0, while the relative intensities of the SADS for the $^3MC_1$ and $^3MC_2$ excited states were found to vary systematically with the c_1 value. As distinct ^{3}MC excited states are not expected to generate measurably different Kβ mainline XES spectra (as discussed in Section 2.2.2 of the main text), c_1 was determined from a new fit in which the difference between the SADS of the $^3MC_1$ and $^3MC_2$ excited states was minimized, while the other parameters were fixed as obtained from Fit 2 (Fit 3). Final fit values were then obtained in a subsequent fit where the lifetimes, IRF FWHM, and t_0 were allowed to vary but c_1 was fixed as obtained from Fit 3 (Fit 4). In this study, the results of Fit 4 are presented and analyzed, except that the c_1 fit value is taken from Fit 3.

The measured data, global analysis fit, and residual are shown in Figure S11a, and the kinetic trace fits at 7057.6 and 7060.1 eV are presented in Figure 5d. These demonstrate that a good fit was obtained. The SADS of the $^3MC_1$ and $^3MC_2$ excited states, which are nearly identical, are shown in Figures S11b and 5f, and the time-dependent populations of these excited state species are shown in Figure S11c. The fit results are summarized in Table S4. The SADS of the $^3MC_1$ and $^3MC_2$ excited states are well fit with the triplet reference difference spectrum but not with the quintet spectrum (Figure S11b), confirming that the two dynamically distinct ^{3}MC excited states form from the initial ^{1,3}MLCT excited state and subsequently decay with the different lifetimes. We note that the IRF FWHM obtained from this global analysis (137 fs; Table S4) is reasonably close to that from the global analysis of the transient Kβ mainline XES difference data in the −0.2 to 0.4 ps delay time range (145 fs; see SI Note 4).

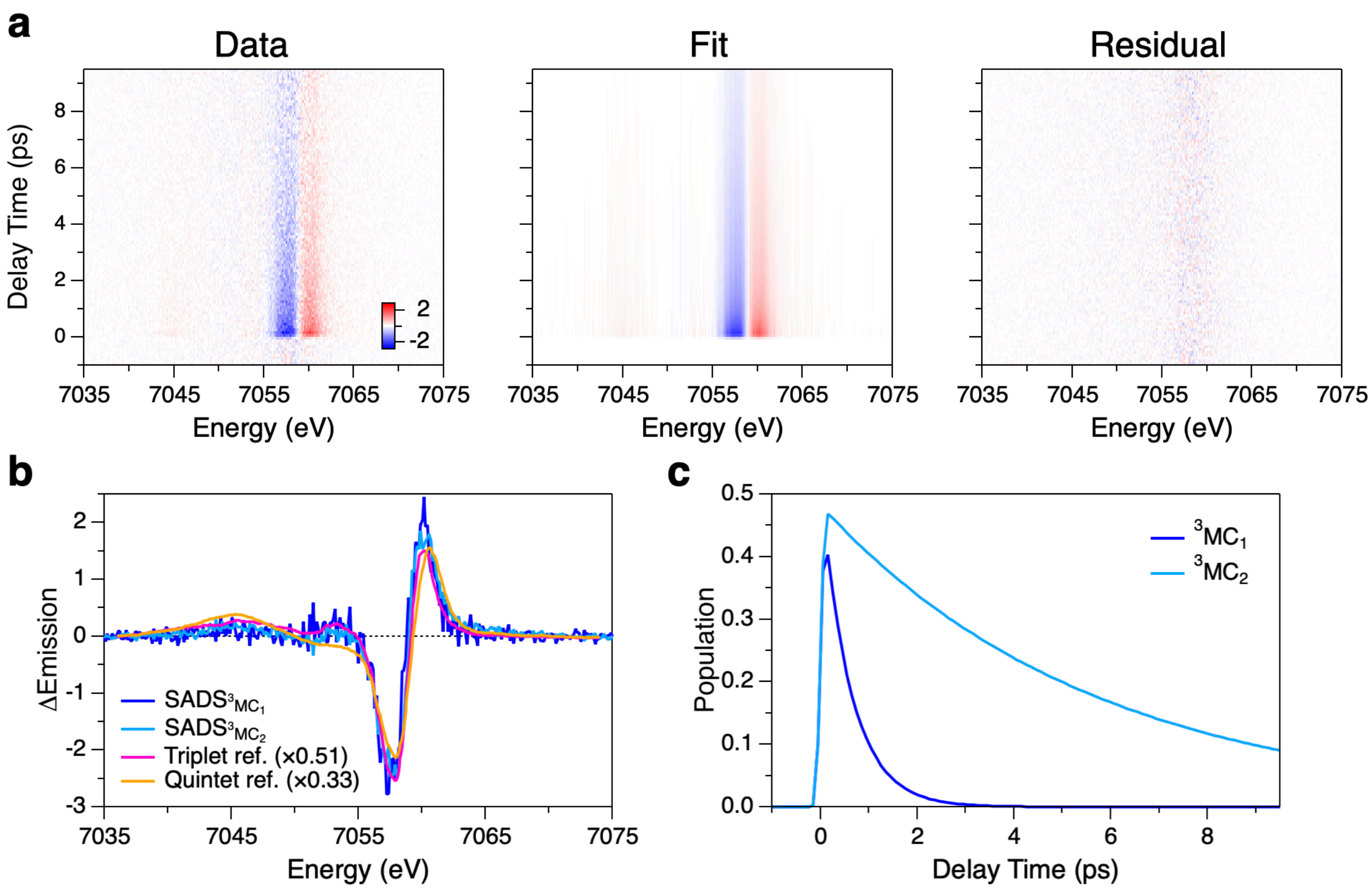

Figure S11. Time-resolved Kβ mainline XES data of complex **1** in methanol. (a) Measured transient Kβ mainline XES difference data (left), global analysis fit to the data (middle), and residual (right) in the −1.0 to 9.5 ps delay time range. (b) SADS of the $^3MC_1$ and $^3MC_2$ excited states from the global analysis, and fits to these SADS with the triplet and quintet reference difference spectra. (c) Time-dependent populations of the $^3MC_1$ and $^3MC_2$ excited states from the global analysis.

Table S4. Fit results from the global analysis of the transient Kβ mainline XES difference data of complex **1** in the −1.0 to 9.5 ps delay time range.[a]

$\tau_{^3MC_1}$ (ps)	0.609 ± 0.035
$\tau_{^3MC_2}$ (ps)	5.656 ± 0.151
c_1	0.517 ± 0.006
IRF FWHM (ps)	0.137 ± 0.008
R^2	0.753

[a]Uncertainties represent one standard deviation error on the fit parameters that is calculated from the covariance matrix (in the fit using the LMFIT package). The c_1 fit value is taken from Fit 3, while the other fit values are obtained from Fit 4.

SI Note 6: Dependence of the Transient Kβ Mainline XES Difference Data on Laser Pulse Energy

6.1. Laser pulse energy titration scan

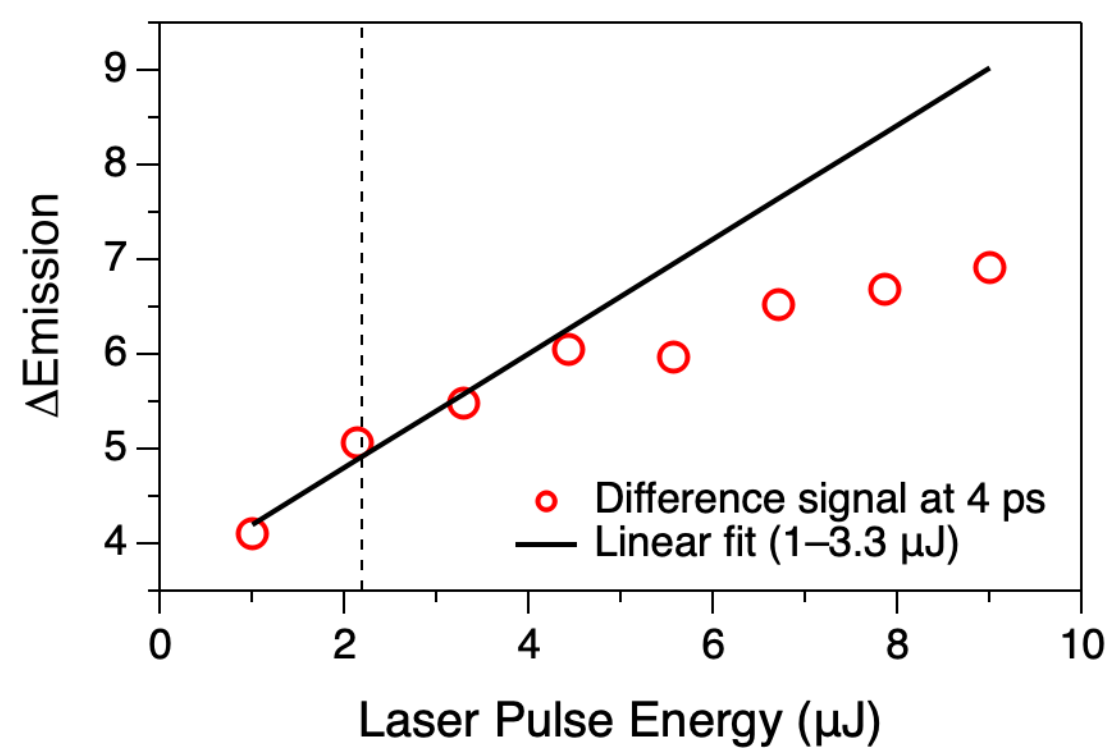

Figure S12. Laser pulse energy titration scan using the integrated transient Kβ mainline XES difference signals of complex **1** in methanol at a 4 ps delay time. A linear fit is conducted for the 1–3.3 μJ range. This fit shows that the difference signal is linear up to ~3–4 μJ. A vertical dashed line indicates the laser pulse energy (2.2 μJ) used for sample excitation in the transient Kβ XES measurements in this study.

6.2. Transient Kβ mainline XES difference data with different laser pulse energies

To rule out multiphoton ionization as the origin of the persistent difference signal (at delay times ≥20 ps) discussed in Section 2.3 of the main text, we examined the dependence of the transient Kβ mainline XES difference data of complex **1** on the laser pump pulse energy. The kinetic traces collected with a laser pulse energy of 2.2 μJ, the energy used for sample excitation in the transient Kβ XES measurements in this study (SI Note 1.3), exhibit decay profiles that are indistinguishable from those measured with a lower pulse energy of 0.87 μJ, within the signal-to-noise ratio of the data (Figure S13). This provides strong evidence that the persistent difference signal observed at delay times ≥20 ps does not result from multiphoton ionization.

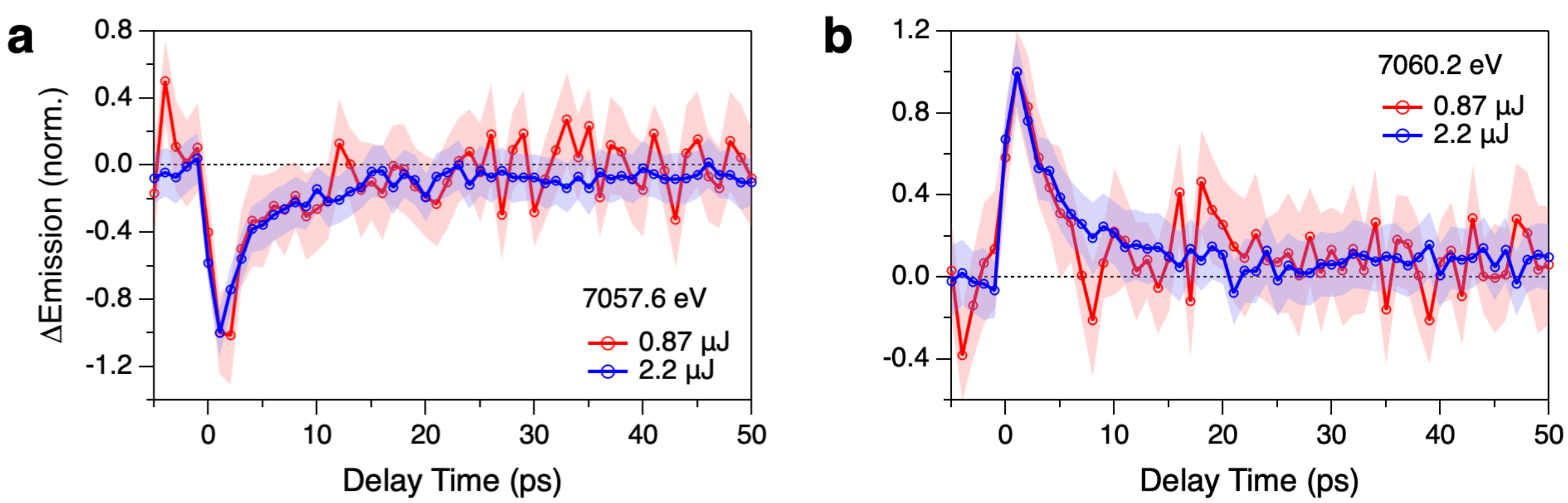

Figure S13. Kinetic traces of complex **1** in methanol at (a) 7057.6 and (b) 7060.2 eV (averaged over 2 eV range for each energy) for sample excitation laser pulse energies of 0.87 and 2.2 μJ. The kinetic traces are normalized such that their maximum magnitudes are set to 1.0. Shaded areas represent the standard deviation from the data averaging.

SI Note 7: Additional DFT Calculations of the Fe Complexes

7.1. Benchmark study of functionals

As discussed in the Discussion section of the main text, it has been shown that functionals with a higher fraction of Hartree–Fock exchange tend to stabilize high-spin states relative to low-spin states.[39,40] Thus, we conducted a benchmark study to compare results from the different functionals, namely BP86, B3LYP, and PBE0, which incorporate 0%, 20%, and 25% Hartree–Fock exchange, respectively. The relative energies of the ^{3}MC and ^{5}MC states with respect to the ground state, based on the DFT optimized structures, are presented in Table S5 and Figure S14. We note that geometry optimizations targeting the ^{3}MLCT state were also attempted, but this state was obtained in only a few cases (see SI Note 1.5). Thus, only the ^{3}MC and ^{5}MC states are considered here.

As discussed in the Discussion section of the main text, the experimental results showed that complex **1** and $[Fe(bpy)(CN)_4]^{2-}$ (in protic solvents) undergo the excited state relaxation through the long-lived ^{3}MC excited state that decays to the ground state without significant formation of the ^{5}MC excited state.[41] In contrast, it was shown that $[Fe(bpy)_3]^{2+}$ and $Fe(bpy)_2(CN)_2$, as well as $[Fe(phen)_3]^{2+}$ and $Fe(phen)_2(CN)_2$, relax via the long-lived ^{5}MC excited state.[1,10,42,43] (Here, we assume that the relaxation mechanism of $[Fe(phen)(CN)_4]^{2-}$ is similar to that of $[Fe(bpy)(CN)_4]^{2-}$, given that the phen and bpy ligands would exhibit similar behavior.) These suggest that the ^{3}MC excited state should be lower in energy than the ^{5}MC excited state for complex **1** and $[Fe(phen)(CN)_4]^{2-}$, whereas the opposite trend is expected for $[Fe(phen)_3]^{2+}$ and $Fe(phen)_2(CN)_2$. These expected energy trends are reproduced in both the B3LYP and PBE0 results (Table S5 and Figure S14). However, the PBE0 functional yields the similar energies for the ^{3}MC and ^{5}MC states in complex **1** and $[Fe(phen)(CN)_4]^{2-}$, and the anomalously low energy for the ^{5}MC state in $[Fe(phen)_3]^{2+}$, which is too close to that of the ground state. Thus, we consider the B3LYP functional to be the most appropriate for comparing the excited state energetics of the Fe complexes considered in this study.

Table S5. Relative energies (eV) of the ^{3}MC and ^{5}MC states with respect to the ground state, based on the DFT optimized structures of the Fe complexes.

		Complex **1**	$[Fe(phen)_3]^{2+}$	$Fe(phen)_2(CN)_2$	$[Fe(phen)(CN)_4]^{2-}$
BP86	^{3}MC	1.46	1.15	1.43	1.50
	^{5}MC	2.32	1.47	1.90	2.62
B3LYP	^{3}MC	0.65	0.56	0.61	0.78
	^{5}MC	0.87	0.23	0.49	0.96
PBE0	^{3}MC	0.67	0.65	0.63	0.83
	^{5}MC	0.69	0.00[a]	0.34	0.89

[a]Although shown as 0.00 eV due to rounding, this corresponds to 0.05 kcal/mol.

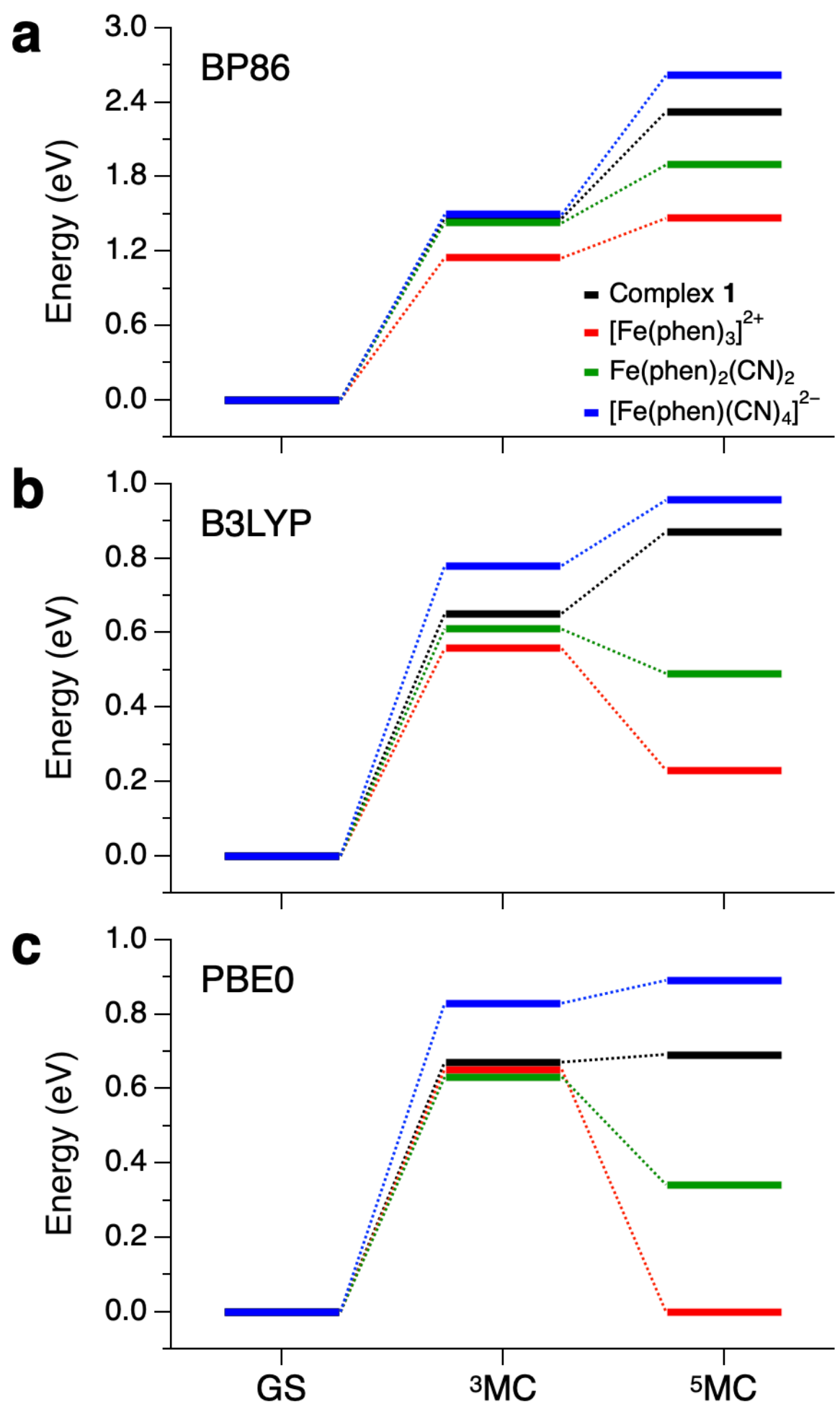

Figure S14. Relative energies of the ^{3}MC and ^{5}MC states with respect to the ground state (GS), based on the DFT optimized structures of the Fe complexes for the (a) BP86, (b) B3LYP, and (c) PBE0 functionals (Table S5).

7.2. Calculations of complex 1, $[Fe(phen)_3]^{2+}$, $Fe(phen)_2(CN)_2$, and $[Fe(phen)(CN)_4]^{2-}$

DFT calculations were performed to gain insight into the excited state relaxation mechanisms of complex **1**, $[Fe(phen)_3]^{2+}$, $Fe(phen)_2(CN)_2$, and $[Fe(phen)(CN)_4]^{2-}$. The optimized structures of the ground states of these complexes are shown in Figures S15 and 8a, and are broadly similar to those of their respective ^{3}MC and ^{5}MC states. The energies, Fe–ligand bond lengths, bond angles, and spin populations on Fe of the optimized structures of complex **1**, $[Fe(phen)_3]^{2+}$, $Fe(phen)_2(CN)_2$, and $[Fe(phen)(CN)_4]^{2-}$ are summarized in Tables S6–S9, respectively. Note that the spin population on Fe for each state (i.e., 0, ~2, and ~4 for the ground, ^{3}MC, and ^{5}MC states, respectively) confirms that the optimized structures obtained correspond to the intended states.

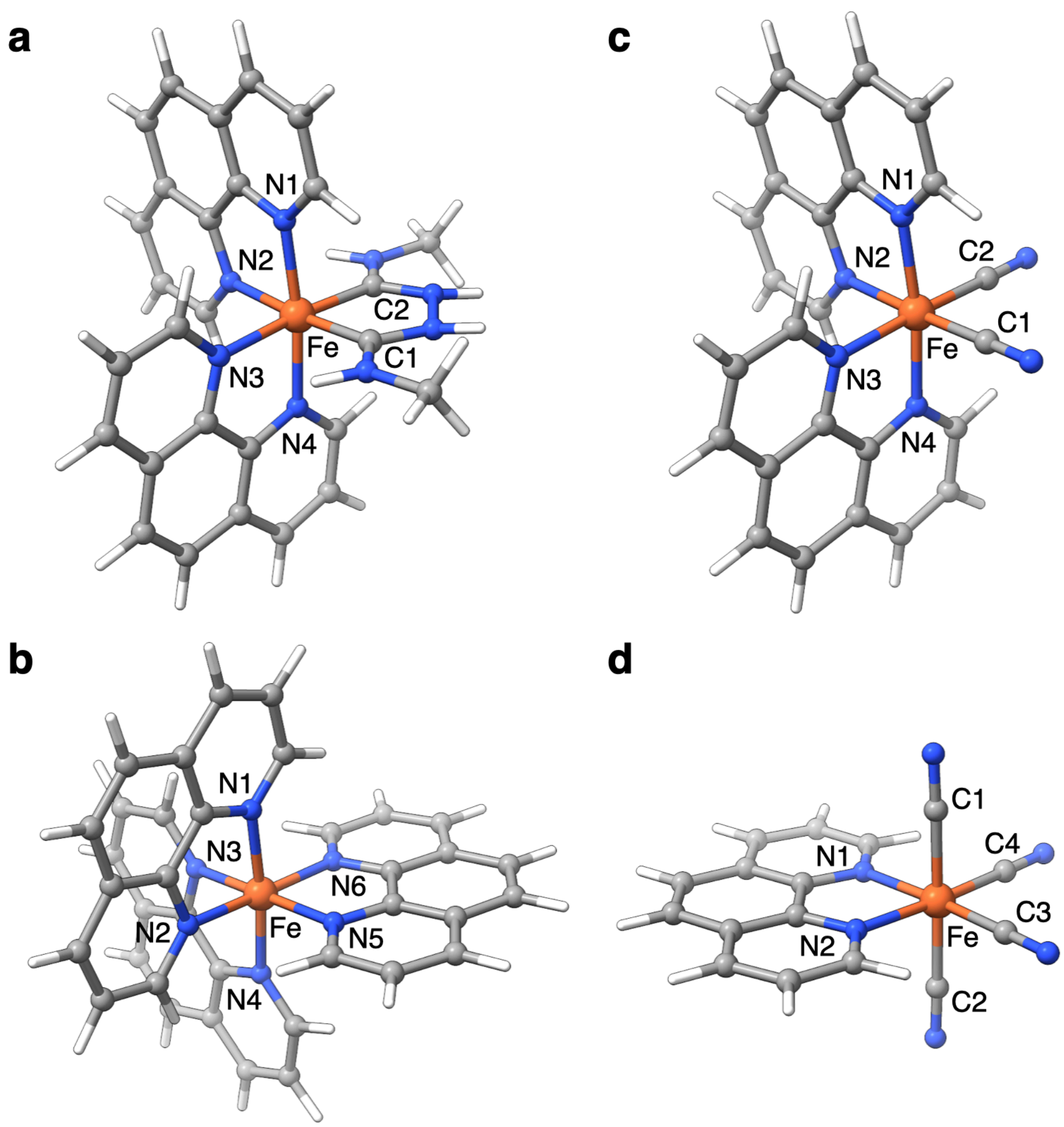

Figure S15. B3LYP DFT optimized structures of the ground states of (a) complex **1**, (b) $[Fe(phen)_3]^{2+}$, (c) $Fe(phen)_2(CN)_2$, and (d) $[Fe(phen)(CN)_4]^{2-}$. Fe is shown in orange, N in blue, C in gray, and H in white.

Table S6. Energies (eV), Fe–ligand bond lengths (Å), bond angles (º), and spin populations on Fe for the B3LYP DFT optimized structures of the ground, ^{3}MC and ^{5}MC states of complex **1**. Numbering for C and N atoms corresponds to that in Figure S15a.

	Ground state	^{3}MC state	^{5}MC state
Energy	0.00	0.65	0.87
Bond length			
Fe–C1	1.95	1.99	2.17
Fe–C2	1.94	1.98	2.17
Fe–N1	2.00	2.29	2.22
Fe–N2	2.04	2.07	2.24
Fe–N3	2.04	2.06	2.23
Fe–N4	2.00	2.29	2.23
Bond angle			
C1–Fe–C2	81.96	80.16	77.69
Spin population on Fe			
Mulliken	0.00	2.11	3.79
Löwdin	0.00	2.02	3.69

Table S7. Energies (eV), Fe–ligand bond lengths (Å), and spin populations on Fe for the B3LYP DFT optimized structures of the ground, ^{3}MC and ^{5}MC states of $[Fe(phen)_3]^{2+}$. Numbering for N atoms corresponds to that in Figure S15b.

	Ground state	^{3}MC state	^{5}MC state
Energy	0.00	0.56	0.23
Bond length			
Fe–N1	2.00	2.00	2.18
Fe–N2	2.00	2.15	2.18
Fe–N3	2.00	2.16	2.20
Fe–N4	2.00	2.00	2.18
Fe–N5	2.00	2.23	2.20
Fe–N6	2.00	2.20	2.18
Spin population on Fe			
Mulliken	0.00	2.01	3.81
Löwdin	0.00	1.95	3.72

Table S8. Energies (eV), Fe–ligand bond lengths (Å), bond angles (º), and spin populations on Fe for the B3LYP DFT optimized structures of the ground, ^{3}MC and ^{5}MC states of $Fe(phen)_2(CN)_2$. Numbering for C and N atoms corresponds to that in Figure S15c.

	Ground state	^{3}MC state	^{5}MC state
Energy	0.00	0.61	0.49
Bond length			
Fe–C1	1.93	1.93	2.15
Fe–C2	1.93	2.02	2.15
Fe–N1	2.00	2.22	2.21
Fe–N2	2.04	2.04	2.30
Fe–N3	2.03	2.14	2.30
Fe–N4	2.01	2.28	2.21
Bond angle			
C1–Fe–C2	90.83	91.49	99.90
Spin population on Fe			
Mulliken	0.00	2.06	3.82
Löwdin	0.00	1.97	3.71

Table S9. Energies (eV), Fe–ligand bond lengths (Å), and spin populations on Fe for the B3LYP DFT optimized structures of the ground, ^{3}MC and ^{5}MC states of $[Fe(phen)(CN)_4]^{2-}$. Numbering for C and N atoms corresponds to that in Figure S15d.

	Ground state	^{3}MC state	^{5}MC state
Energy	0.00	0.78	0.96
Bond length			
Fe–C1	1.95	1.96	2.25
Fe–C2	1.95	1.96	2.25
Fe–C3	1.92	2.10	2.20
Fe–C4	1.92	2.09	2.20
Fe–N1	2.04	2.28	2.30
Fe–N2	2.03	2.29	2.30
Spin population on Fe			
Mulliken	0.00	2.04	3.82
Löwdin	0.00	1.96	3.71

7.3. Calculations of modified complex 1 having two monodentate carbene ligands

DFT calculations were performed to gain insight into the influence of structural rigidity on the relative energies of the ^{3}MC and ^{5}MC excited states. As discussed in the Discussion section of the main text, for complex **1**, breaking the N–N bond in the bidentate carbene ligand generates modified complex **1** which has two monodentate carbene ligands. The optimized structure of the ground state of this modified complex **1** is shown in Figures S16 and 9a, and is broadly similar to those of the ^{3}MC and ^{5}MC states. The energies, Fe–ligand bond lengths, bond angles, and spin populations on Fe of the optimized structures are summarized in Table S10. Note that the spin population on Fe for each state (i.e., 0, ~2, and ~4 for the ground, ^{3}MC, and ^{5}MC states, respectively) confirms that the optimized structures obtained correspond to the intended states.

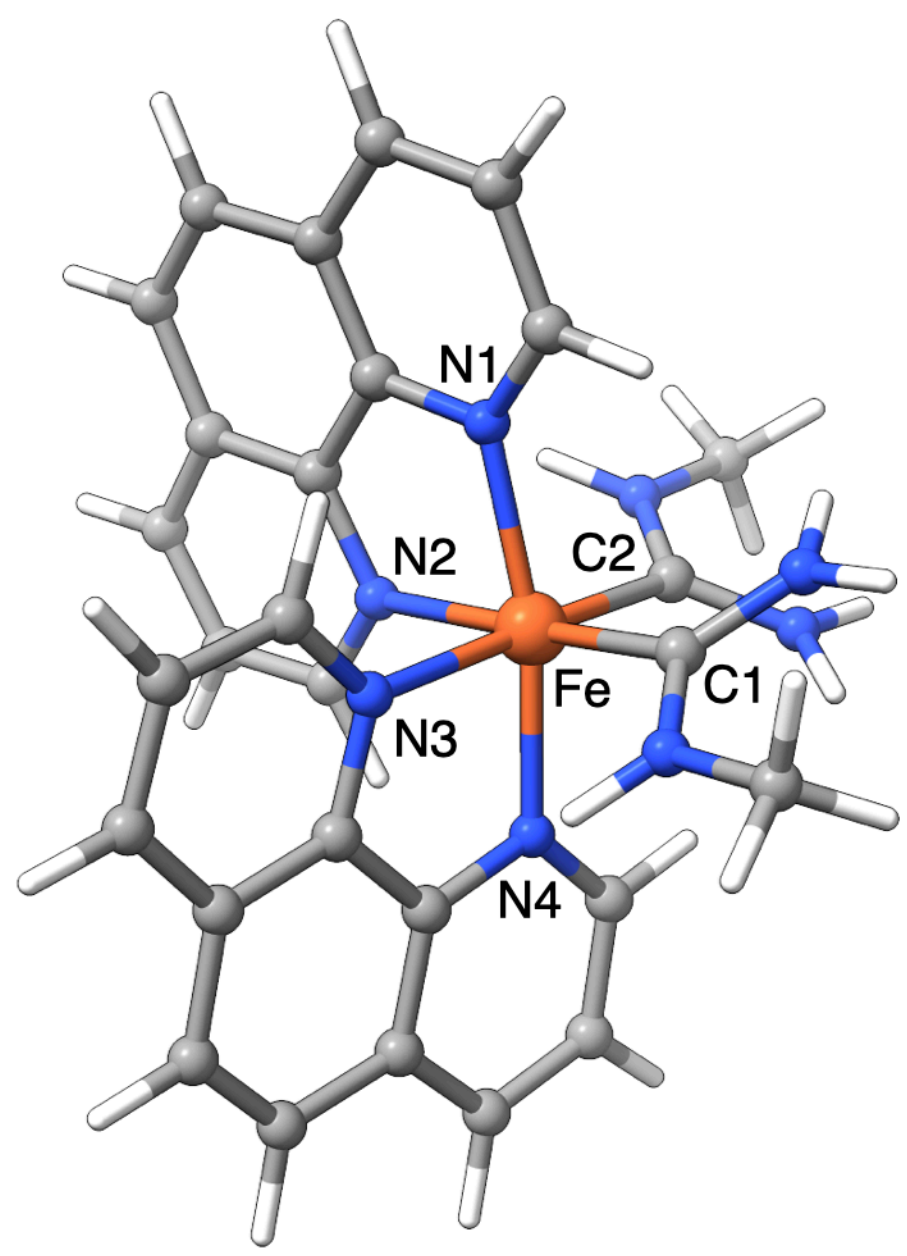

Figure S16. B3LYP DFT optimized structure of the ground state of modified complex **1**. Fe is shown in orange, N in blue, C in gray, and H in white.

Table S10. Energies (eV), Fe–ligand bond lengths (Å), bond angles (º), and spin populations on Fe for the B3LYP DFT optimized structures of the ground, ^{3}MC and ^{5}MC states of modified complex **1**. Numbering for C and N atoms corresponds to that in Figure S16.

	Ground state	^{3}MC state	^{5}MC state
Energy	0.00	0.49	0.50
Bond length			
Fe–C1	1.99	2.00	2.19
Fe–C2	2.00	2.00	2.19
Fe–N1	2.02	2.36	2.23
Fe–N2	2.05	2.10	2.32
Fe–N3	2.05	2.09	2.31
Fe–N4	2.02	2.35	2.24
Bond angle			
C1–Fe–C2	93.05	90.92	99.07
Spin population on Fe			
Mulliken	0.00	2.15	3.81
Löwdin	0.00	2.03	3.70

7.4. Calculations of $[Fe(phen)_2(CN^{tBu}Ar_3NC)]^{2+}$

DFT calculations were performed to gain insight into the influence of structural rigidity on the relative energies of the ^{3}MC and ^{5}MC excited states. As discussed in the Discussion section of the main text, for $Fe(phen)_2(CN)_2$, replacing the two monodentate cyanide ligands with a bidentate diisocyanide ligand generates $[Fe(phen)_2(CN^{tBu}Ar_3NC)]^{2+}$.[37] The optimized structure of the ground state of $[Fe(phen)_2(CN^{tBu}Ar_3NC)]^{2+}$ is shown in Figures S17 and 9a, and is broadly similar to those of the ^{3}MC and ^{5}MC states. The energies, Fe–ligand bond lengths, bond angles, and spin populations on Fe of the optimized structures are summarized in Table S11. Note that the spin population on Fe for each state (i.e., 0, ~2, and ~4 for the ground, ^{3}MC, and ^{5}MC states, respectively) confirms that the optimized structures obtained correspond to the intended states.

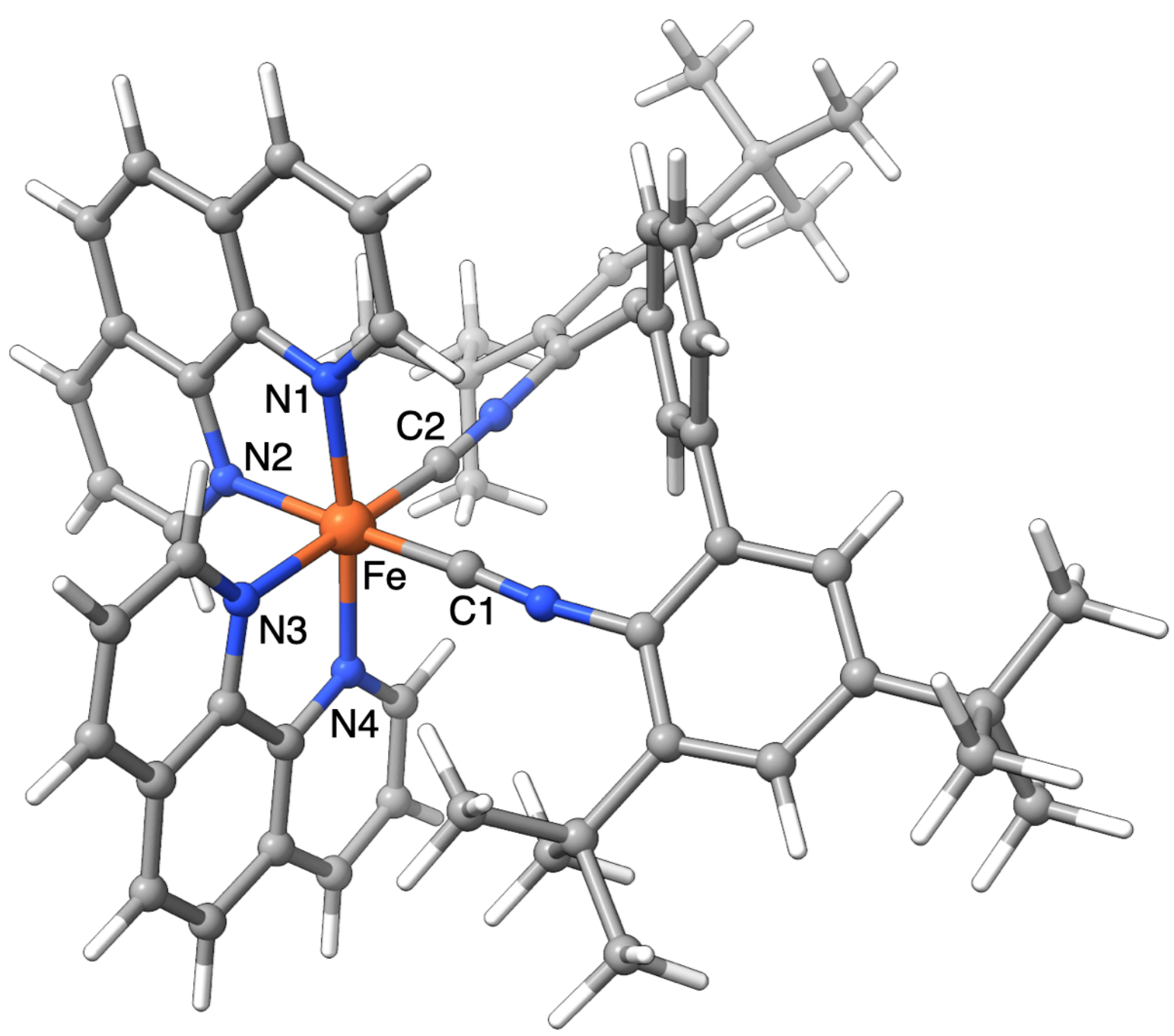

Figure S17. B3LYP DFT optimized structure of the ground state of $[Fe(phen)_2(CN^{tBu}Ar_3NC)]^{2+}$. Fe is shown in orange, N in blue, C in gray, and H in white.

Table S11. Energies (eV), Fe–ligand bond lengths (Å), bond angles (º), and spin populations on Fe for the B3LYP DFT optimized structures of the ground, ^{3}MC and ^{5}MC states of $[Fe(phen)_2(CN^{tBu}Ar_3NC)]^{2+}$. Numbering for C and N atoms corresponds to that in Figure S17.

	Ground state	^{3}MC state	^{5}MC state
Energy	0.00	0.82	0.87
Bond length			
Fe–C1	1.86	1.87	2.14
Fe–C2	1.87	2.00	2.15
Fe–N1	2.01	2.23	2.18
Fe–N2	2.03	2.03	2.22
Fe–N3	2.03	2.10	2.20
Fe–N4	2.01	2.29	2.17
Bond angle			
C1–Fe–C2	88.52	86.75	87.56
Spin population on Fe			
Mulliken	0.00	2.05	3.77
Löwdin	0.00	1.96	3.68

7.5. Calculations of *trans*- and *cis*-$[Fe(py)_2(CN)_4]^{2-}$

DFT calculations were performed to gain insight into the influence of ligand arrangement on the relative energies of the ^{3}MC and ^{5}MC excited states. As discussed in the Discussion section of the main text, two $[Fe(py)_2(CN)_4]^{2-}$ isomers were considered: *trans*-$[Fe(py)_2(CN)_4]^{2-}$, in which the two moderate field py ligands occupy the axial positions, and *cis*-$[Fe(py)_2(CN)_4]^{2-}$, where the two py ligands neighbor each other. The optimized structures of the ground states of these isomers are shown in Figures S18 and 10a, and are broadly similar to those of their respective ^{3}MC and ^{5}MC states. The energies, Fe–ligand bond lengths, and spin populations on Fe of the optimized structures of *trans*- and *cis*-$[Fe(py)_2(CN)_4]^{2-}$ are summarized in Tables S12 and S13, respectively. Note that the spin population on Fe for each state (i.e., 0, ~2, and ~4 for the ground, ^{3}MC, and ^{5}MC states, respectively) confirms that the optimized structures obtained correspond to the intended states.

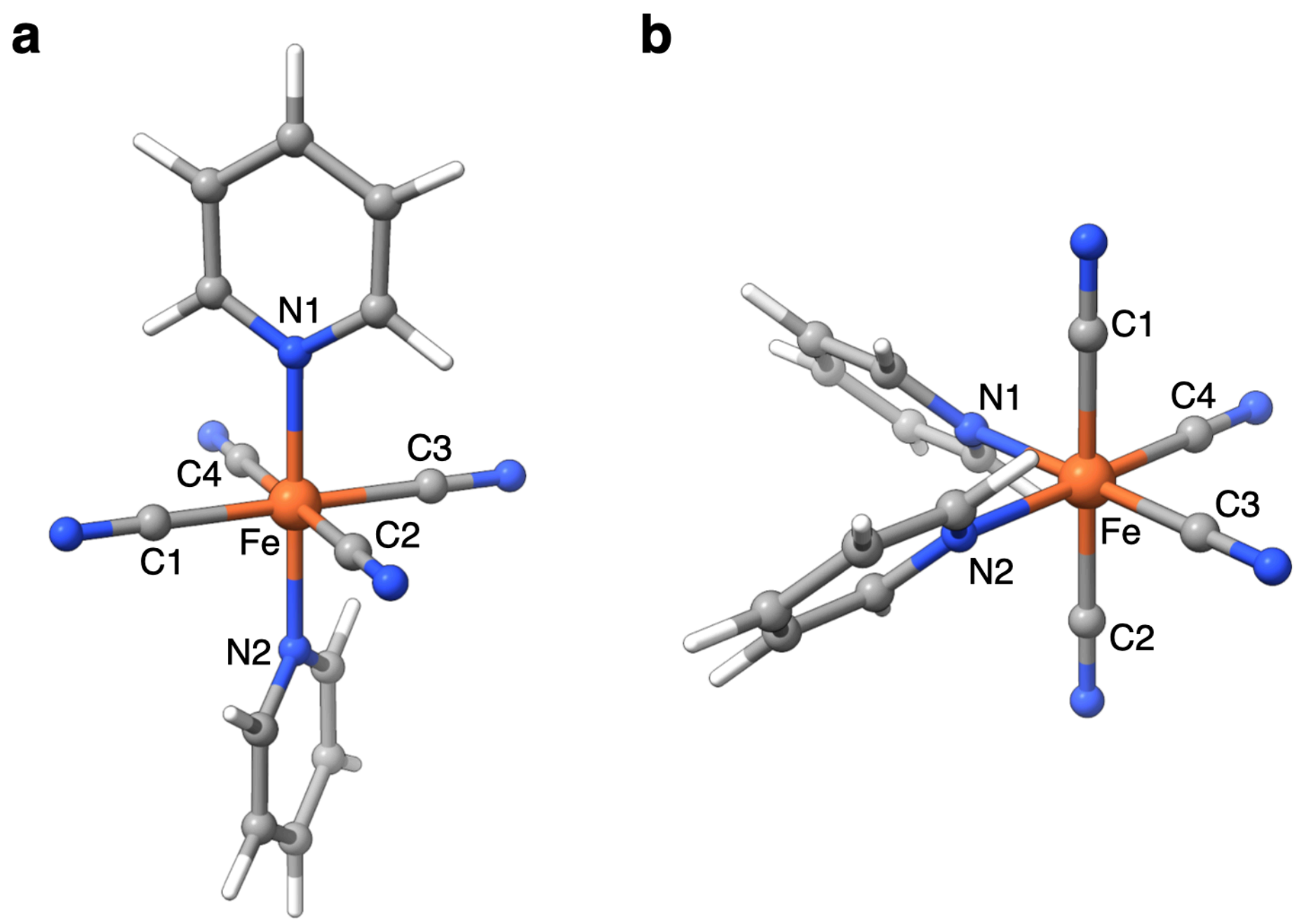

Figure S18. B3LYP DFT optimized structures of the ground states of (a) *trans*-$[Fe(py)_2(CN)_4]^{2-}$ and (b) *cis*-$[Fe(py)_2(CN)_4]^{2-}$. Fe is shown in orange, N in blue, C in gray, and H in white.

Table S12. Energies (eV), Fe–ligand bond lengths (Å), and spin populations on Fe for the B3LYP DFT optimized structures of the ground, ^{3}MC and ^{5}MC states of *trans*-$[Fe(py)_2(CN)_4]^{2-}$. Numbering for C and N atoms corresponds to that in Figure S18a.

	Ground state	^{3}MC state	^{5}MC state
Energy	0.00	0.44	0.82
Bond length			
Fe–C1	1.96	1.99	2.25
Fe–C2	1.96	1.99	2.25
Fe–C3	1.96	1.99	2.25
Fe–C4	1.96	1.99	2.25
Fe–N1	2.06	2.47	2.28
Fe–N2	2.06	2.47	2.28
Spin population on Fe			
Mulliken	0.00	2.12	3.84
Löwdin	0.00	1.98	3.72

Table S13. Energies (eV), Fe–ligand bond lengths (Å), and spin populations on Fe for the B3LYP DFT optimized structures of the ground, ^{3}MC and ^{5}MC states of *cis*-$[Fe(py)_2(CN)_4]^{2-}$. Numbering for C and N atoms corresponds to that in Figure S18b.

	Ground state	^{3}MC state	^{5}MC state
Energy	0.00	0.74	0.84
Bond length			
Fe–C1	1.96	1.96	2.21
Fe–C2	1.96	1.95	2.22
Fe–C3	1.92	2.09	2.20
Fe–C4	1.92	2.09	2.20
Fe–N1	2.08	2.36	2.39
Fe–N2	2.08	2.36	2.39
Spin population on Fe			
Mulliken	0.00	2.03	3.81
Löwdin	0.00	1.94	3.70

SI Note 8: Cartesian Coordinates of the DFT Optimized Structures

Complex 1 – Ground state (BP86)

Fe	0.02837	-0.00336	-0.09186
N	-1.70758	0.93918	1.83354
N	-0.82269	2.00082	1.60212
N	-2.42474	-1.09761	1.07748
N	1.15274	2.60766	0.62153
N	1.07763	-0.95088	1.25450
N	-0.18883	-1.84116	-0.84632
N	1.61052	0.26070	-1.28381
N	-0.92023	0.82612	-1.58434
C	-1.52228	-0.11736	1.00900
C	0.19216	1.69258	0.76146
C	-3.59439	-1.11515	1.95090
C	1.20190	3.90001	1.29938
C	1.69412	-0.46390	2.34361
C	2.43473	-1.27564	3.21674
C	2.55610	-2.63515	2.97542
C	1.91620	-3.18364	1.84258
C	1.96294	-4.57047	1.47524
C	1.31759	-5.03296	0.35922
C	0.56387	-4.14862	-0.48282
C	-0.13472	-4.54731	-1.64406
C	-0.83164	-3.59187	-2.36725
C	-0.83998	-2.25219	-1.94419
C	0.50615	-2.77878	-0.13290
C	1.18353	-2.29744	1.01744
C	2.89702	-0.06846	-1.09634
C	3.87683	0.12754	-2.08397
C	3.53198	0.67879	-3.30814
C	2.18444	1.03312	-3.54134
C	1.69399	1.61337	-4.75855
C	0.37076	1.93375	-4.90786
C	-0.57701	1.69530	-3.85660
C	-1.95452	1.99327	-3.94163
C	-2.76623	1.70745	-2.85525
C	-2.22394	1.12733	-1.69782
C	-0.10981	1.11801	-2.65172
C	1.26332	0.79665	-2.49361
H	-4.29519	-0.30622	1.69006
H	-3.29197	-0.99957	3.00259
H	-4.10555	-2.07497	1.83130
H	0.29753	4.48669	1.07813
H	2.07851	4.44804	0.94155
H	1.28549	3.77217	2.39034
H	1.60416	0.60623	2.51796
H	2.91085	-0.81310	4.08047
H	3.13295	-3.27820	3.64087
H	2.53017	-5.25283	2.10973
H	1.36329	-6.08941	0.09084
H	-0.12026	-5.59229	-1.95603
H	-1.38285	-3.86047	-3.26768
H	-1.39140	-1.49580	-2.50051
H	3.15945	-0.49433	-0.12907
H	4.90496	-0.15916	-1.86610
H	4.27953	0.84093	-4.08554
H	2.40337	1.79707	-5.56672
H	0.00908	2.37711	-5.83663
H	-2.36091	2.43863	-4.85030
H	-3.83396	1.92208	-2.87889
H	-2.85859	0.89081	-0.84614
H	-0.75602	2.66452	2.37525
H	-2.28603	-1.88885	0.45812
H	1.91202	2.37548	-0.00909
H	-2.60407	1.22048	2.23324

Complex 1 – ^{3}MLCT state (BP86)

Fe	0.00411	-0.01199	-0.07884
N	-1.76426	1.00036	1.79767
N	-0.81018	1.99977	1.64173
N	-2.35751	-1.14699	1.24331
N	1.03363	2.68019	0.45290
N	0.99814	-0.99786	1.29031
N	-0.23015	-1.85661	-0.88000
N	1.62049	0.21409	-1.23240
N	-0.92215	0.83327	-1.58108
C	-1.54446	-0.11271	1.08252
C	0.13939	1.74198	0.72971
C	-3.50574	-1.18399	2.14732
C	1.08960	4.00320	1.07199
C	1.59477	-0.52542	2.40516
C	2.38047	-1.33653	3.22293
C	2.55828	-2.68901	2.91326
C	1.93030	-3.21658	1.76939
C	2.00244	-4.59227	1.35815
C	1.33540	-5.04603	0.25089
C	0.54766	-4.16025	-0.55597
C	-0.16369	-4.53936	-1.70908
C	-0.89144	-3.58092	-2.41080
C	-0.90364	-2.24996	-1.96623
C	0.48459	-2.79229	-0.17328
C	1.15930	-2.32870	0.97569
C	2.90794	-0.09531	-1.00485
C	3.90475	0.10062	-1.96405
C	3.57528	0.64310	-3.21303
C	2.24254	0.98800	-3.48201
C	1.77695	1.57326	-4.70818
C	0.46168	1.91108	-4.88151
C	-0.51540	1.69369	-3.84833
C	-1.86956	2.04945	-3.94181
C	-2.71393	1.81306	-2.84490
C	-2.21236	1.22039	-1.68995
C	-0.07060	1.08636	-2.64185
C	1.28052	0.75039	-2.45918
H	-4.21146	-0.37554	1.90481
H	-3.17894	-1.07708	3.19282
H	-4.00905	-2.14739	2.02822
H	0.17645	4.57492	0.84730
H	1.95345	4.53706	0.66649
H	1.19954	3.91249	2.16301
H	1.43900	0.52675	2.63637
H	2.84059	-0.89810	4.10734
H	3.16482	-3.33517	3.54825
H	2.60127	-5.27686	1.96074
H	1.39602	-6.09583	-0.04020
H	-0.13908	-5.57791	-2.04246
H	-1.44588	-3.84178	-3.31088
H	-1.46486	-1.48203	-2.49762
H	3.14554	-0.50870	-0.02528
H	4.92968	-0.17704	-1.72343
H	4.34153	0.80566	-3.97217
H	2.50297	1.75340	-5.50271
H	0.12749	2.36587	-5.81552
H	-2.24995	2.51128	-4.85337
H	-3.76937	2.07889	-2.88109
H	-2.85674	1.02138	-0.83520
H	-1.07197	2.91457	2.01160
H	-2.17644	-1.96662	0.67218
H	1.74979	2.43982	-0.22540
H	-2.36471	1.09325	2.61815

Complex 1 – ^{3}MC state (BP86)

Fe	0.05265	-0.03184	-0.12555
N	-1.69565	0.93996	1.83881
N	-0.82901	2.00020	1.59370
N	-2.39242	-1.12031	1.12459
N	1.11820	2.63613	0.57388
N	1.32482	-1.06826	1.33635
N	-0.08839	-1.94986	-0.76134
N	1.55526	0.30906	-1.43671
N	-1.04823	0.79490	-1.84374
C	-1.48990	-0.13967	1.05966
C	0.15495	1.72388	0.71593
C	-3.59583	-1.11358	1.95147
C	1.20466	3.90110	1.29862
C	1.96363	-0.59859	2.41091
C	2.60715	-1.43844	3.33513
C	2.59194	-2.81071	3.13568
C	1.91774	-3.33890	2.01308
C	1.85623	-4.73729	1.70284
C	1.20150	-5.18221	0.58723
C	0.53313	-4.27041	-0.29624
C	-0.17182	-4.67910	-1.44883
C	-0.80013	-3.72144	-2.22896
C	-0.74063	-2.37105	-1.85575
C	0.56293	-2.88300	0.00008
C	1.28172	-2.41390	1.14338
C	2.84597	-0.01084	-1.25501
C	3.83588	0.26284	-2.20972
C	3.49296	0.89451	-3.39451
C	2.14249	1.23496	-3.62418
C	1.68690	1.90623	-4.80755
C	0.36869	2.23044	-4.97603
C	-0.60785	1.88482	-3.98487
C	-1.98596	2.16822	-4.10546
C	-2.85172	1.77047	-3.09775
C	-2.34487	1.08257	-1.98262
C	-0.18583	1.20186	-2.81351
C	1.19855	0.90257	-2.61828
H	-4.25358	-0.27347	1.67847
H	-3.33467	-1.03045	3.01759
H	-4.13668	-2.05048	1.78912
H	0.32573	4.53004	1.08932
H	2.10631	4.42817	0.97331
H	1.26803	3.72682	2.38426
H	1.97631	0.48608	2.53238
H	3.12136	-0.99929	4.18955
H	3.09441	-3.48582	3.82999
H	2.35155	-5.44028	2.37437
H	1.16658	-6.24670	0.35045
H	-0.21533	-5.73756	-1.70899
H	-1.35258	-3.99730	-3.12639
H	-1.24230	-1.60323	-2.44267
H	3.09662	-0.48696	-0.30820
H	4.86667	-0.01755	-1.99634
H	4.24607	1.13295	-4.14675
H	2.42432	2.16159	-5.57002
H	0.03410	2.75159	-5.87434
H	-2.35345	2.69185	-4.98927
H	-3.92102	1.96948	-3.16308
H	-3.00669	0.73875	-1.18560
H	-0.79773	2.70852	2.32842
H	-2.21572	-1.94169	0.55642
H	1.83813	2.43647	-0.11175
H	-2.57328	1.19788	2.29257

Complex 1 – ^{5}MC state (BP86)

Fe	0.09985	-0.08149	-0.19571
N	-1.73901	0.95297	1.79233
N	-0.77133	1.96264	1.66588
N	-2.59401	-1.05580	1.14350
N	1.16476	2.74098	0.75182
N	1.33814	-1.17462	1.21580
N	-0.11144	-2.18216	-0.83769
N	1.74375	0.35438	-1.59792
N	-0.91497	0.74644	-1.92832
C	-1.63170	-0.13949	1.03441
C	0.24277	1.78017	0.81833
C	-3.76598	-0.97742	2.01345
C	1.17085	3.98516	1.51938
C	2.06254	-0.66369	2.21536
C	2.74149	-1.46398	3.14838
C	2.65694	-2.84297	3.04902
C	1.89808	-3.41588	2.00414
C	1.76164	-4.83120	1.81715
C	1.03359	-5.33872	0.77721
C	0.38154	-4.46983	-0.15825
C	-0.36977	-4.93829	-1.25918
C	-0.96220	-4.02864	-2.11904
C	-0.81091	-2.65350	-1.87131
C	0.48754	-3.06341	0.00852
C	1.25564	-2.52966	1.09920
C	3.04675	0.12589	-1.42522
C	4.01839	0.55009	-2.34780
C	3.61845	1.23796	-3.48124
C	2.24484	1.49106	-3.69469
C	1.74106	2.18858	-4.84145
C	0.39926	2.39280	-5.00601
C	-0.54607	1.91407	-4.03954
C	-1.94248	2.08093	-4.17427
C	-2.78344	1.57692	-3.19577
C	-2.23036	0.92026	-2.08452
C	-0.07671	1.22685	-2.88883
C	1.33410	1.01677	-2.71357
H	-4.37427	-0.09211	1.77012
H	-3.46431	-0.92019	3.07095
H	-4.37477	-1.87467	1.86686
H	0.27092	4.58157	1.30232
H	2.05615	4.56622	1.24377
H	1.20562	3.77718	2.60043
H	2.10296	0.42517	2.27422
H	3.32177	-0.98857	3.93851
H	3.16803	-3.49228	3.76144
H	2.25938	-5.49578	2.52504
H	0.93743	-6.41648	0.63707
H	-0.47243	-6.01273	-1.41960
H	-1.54325	-4.35871	-2.97935
H	-1.27120	-1.91009	-2.52395
H	3.32902	-0.41090	-0.51821
H	5.06933	0.33517	-2.15800
H	4.34793	1.58592	-4.21443
H	2.45575	2.54951	-5.58263
H	0.01959	2.92137	-5.88180
H	-2.34024	2.60114	-5.04696
H	-3.86514	1.68337	-3.27049
H	-2.86675	0.51991	-1.29370
H	-0.89769	2.72784	2.32925
H	-2.49725	-1.88487	0.56694
H	1.92505	2.59547	0.09585
H	-2.51038	1.20812	2.41034

Complex 1 – Ground state (B3LYP)

Fe	0.03112	-0.01689	-0.10120
N	-1.71790	0.95225	1.80488
N	-0.80423	1.99085	1.60393
N	-2.46087	-1.08004	1.11485
N	1.14949	2.63443	0.64390
N	1.11173	-0.99371	1.26423
N	-0.18226	-1.89823	-0.84961
N	1.63142	0.26766	-1.32668
N	-0.93125	0.81953	-1.64145
C	-1.54897	-0.12110	1.02645
C	0.20040	1.71642	0.76537
C	-3.63425	-1.07018	1.97822
C	1.19791	3.91394	1.33917
C	1.73813	-0.51255	2.33207
C	2.47628	-1.32301	3.20275
C	2.57182	-2.67493	2.96324
C	1.91732	-3.21747	1.84274
C	1.94956	-4.60573	1.49213
C	1.29695	-5.06832	0.39385
C	0.54981	-4.18375	-0.44860
C	-0.15430	-4.59491	-1.59517
C	-0.84410	-3.65734	-2.32976
C	-0.83575	-2.31594	-1.92627
C	0.50444	-2.81550	-0.12193
C	1.19399	-2.32945	1.02452
C	2.91099	-0.03005	-1.13885
C	3.89140	0.20663	-2.11116
C	3.53490	0.76558	-3.31733
C	2.18556	1.08969	-3.54910
C	1.70042	1.67840	-4.76096
C	0.38378	1.97421	-4.91840
C	-0.56449	1.70494	-3.87960
C	-1.93912	1.98501	-3.98046
C	-2.76211	1.68020	-2.92048
C	-2.22376	1.09833	-1.76661
C	-0.10986	1.12214	-2.68151
C	1.27064	0.81649	-2.51507
H	-4.29546	-0.23578	1.72949
H	-3.34200	-0.98787	3.02784
H	-4.17817	-1.99991	1.83595
H	0.31373	4.51307	1.10830
H	2.08360	4.45264	1.01391
H	1.25262	3.76916	2.42153
H	1.66324	0.55123	2.50340
H	2.96448	-0.86891	4.05371
H	3.13996	-3.32087	3.62034
H	2.51050	-5.28103	2.12554
H	1.32806	-6.11924	0.13550
H	-0.14757	-5.63807	-1.88495
H	-1.39705	-3.93624	-3.21584
H	-1.37749	-1.57003	-2.49030
H	3.17773	-0.46531	-0.18632
H	4.91819	-0.05448	-1.89629
H	4.27388	0.95994	-4.08431
H	2.41085	1.88518	-5.55128
H	0.02421	2.42097	-5.83662
H	-2.33229	2.43316	-4.88409
H	-3.82385	1.87984	-2.95983
H	-2.86190	0.84829	-0.93165
H	-0.82647	2.71340	2.31145
H	-2.32312	-1.88665	0.52977
H	1.89873	2.42508	0.00654
H	-2.55640	1.21960	2.30359

Complex 1 – ^{3}MC state (B3LYP)

Fe	0.02415	0.02550	-0.07719
N	-1.73123	0.98585	1.88979
N	-0.85324	2.04287	1.66799
N	-2.42516	-1.07891	1.24143
N	1.05482	2.74562	0.65500
N	1.33156	-1.12498	1.41308
N	-0.09941	-1.93006	-0.74546
N	1.55212	0.36749	-1.42149
N	-1.06618	0.89215	-1.89456
C	-1.53853	-0.09916	1.13792
C	0.12454	1.81180	0.79002
C	-3.59929	-1.08319	2.10424
C	1.11272	4.00648	1.38334
C	1.99409	-0.70783	2.47773
C	2.67726	-1.58271	3.33388
C	2.66425	-2.93110	3.05952
C	1.96406	-3.40282	1.93339
C	1.90611	-4.78459	1.56808
C	1.22420	-5.18372	0.46515
C	0.53185	-4.23969	-0.35853
C	-0.19043	-4.61586	-1.50531
C	-0.83630	-3.64989	-2.24018
C	-0.76881	-2.31630	-1.82519
C	0.55933	-2.87036	-0.01811
C	1.29924	-2.44559	1.13666
C	2.83012	0.08599	-1.19664
C	3.83745	0.32786	-2.13612
C	3.50510	0.87907	-3.35110
C	2.15922	1.18367	-3.62335
C	1.73090	1.76222	-4.86078
C	0.42389	2.04817	-5.08617
C	-0.56823	1.77198	-4.09360
C	-1.93801	2.04023	-4.27530
C	-2.82775	1.73602	-3.27067
C	-2.34607	1.15876	-2.08748
C	-0.17904	1.19483	-2.86546
C	1.20564	0.90406	-2.62157
H	-4.27261	-0.26107	1.84855
H	-3.30908	-0.98904	3.15354
H	-4.12753	-2.02260	1.96753
H	0.22535	4.61147	1.18140
H	1.99417	4.55364	1.06110
H	1.18205	3.82993	2.45982
H	1.99313	0.35996	2.66162
H	3.20568	-1.18919	4.19118
H	3.18585	-3.63489	3.69600
H	2.42333	-5.50436	2.18982
H	1.18582	-6.22908	0.18608
H	-0.23010	-5.65869	-1.79374
H	-1.40046	-3.89951	-3.12778
H	-1.27971	-1.54218	-2.37880
H	3.06736	-0.34078	-0.23272
H	4.86038	0.07996	-1.89007
H	4.26092	1.08285	-4.09902
H	2.48038	1.96903	-5.61410
H	0.10678	2.48860	-6.02306
H	-2.27559	2.48233	-5.20437
H	-3.88578	1.93021	-3.37944
H	-3.02259	0.90133	-1.28123
H	-0.88610	2.77425	2.36645
H	-2.25595	-1.90093	0.68659
H	1.77240	2.57330	-0.02853
H	-2.56023	1.22632	2.41777

Complex 1 – ^{5}MC state (B3LYP)

Fe	0.05859	0.00745	-0.10559
N	-1.83047	1.05009	1.87026
N	-0.84326	2.04721	1.79100
N	-2.70634	-0.92786	1.20672
N	1.12105	2.83252	0.99060
N	1.33045	-1.17371	1.28456
N	-0.15126	-2.11002	-0.80215
N	1.72616	0.42045	-1.52884
N	-0.93882	0.83686	-1.91674
C	-1.72426	-0.04195	1.12742
C	0.19048	1.88910	0.97592
C	-3.90340	-0.82316	2.03301
C	1.12314	4.03160	1.82120
C	2.04525	-0.70561	2.29496
C	2.79437	-1.53313	3.14097
C	2.78576	-2.89107	2.92421
C	2.03105	-3.41854	1.85966
C	1.97268	-4.81806	1.56798
C	1.23932	-5.28308	0.52569
C	0.50093	-4.38745	-0.31047
C	-0.26943	-4.82377	-1.40470
C	-0.95088	-3.90344	-2.16584
C	-0.86459	-2.54712	-1.82733
C	0.53180	-3.00094	-0.04690
C	1.31230	-2.50727	1.05597
C	3.01639	0.20700	-1.32744
C	3.99106	0.49750	-2.29045
C	3.59734	1.02471	-3.49781
C	2.23111	1.26101	-3.74089
C	1.74451	1.80705	-4.97046
C	0.41834	2.01899	-5.16227
C	-0.53201	1.70066	-4.14137
C	-1.91667	1.89969	-4.29783
C	-2.76911	1.56735	-3.27133
C	-2.23502	1.03814	-2.08954
C	-0.08602	1.15894	-2.91645
C	1.32065	0.93742	-2.71196
H	-4.48494	0.06254	1.76412
H	-3.64024	-0.76240	3.09206
H	-4.51640	-1.70597	1.87338
H	0.24845	4.65177	1.60930
H	2.02099	4.60478	1.60672
H	1.11890	3.76854	2.88230
H	2.03036	0.36701	2.44153
H	3.36400	-1.09540	3.94884
H	3.35146	-3.56137	3.55909
H	2.53130	-5.49736	2.19924
H	1.19854	-6.34227	0.30522
H	-0.31533	-5.88104	-1.63446
H	-1.54970	-4.20563	-3.01355
H	-1.39349	-1.79984	-2.40454
H	3.29547	-0.20868	-0.36794
H	5.03209	0.30490	-2.07223
H	4.32268	1.26202	-4.26607
H	2.46219	2.04768	-5.74448
H	0.05187	2.43320	-6.09298
H	-2.29349	2.31233	-5.22535
H	-3.83750	1.70716	-3.35883
H	-2.88105	0.76663	-1.26434
H	-0.96758	2.77510	2.48283
H	-2.60836	-1.75610	0.64339
H	1.89632	2.70858	0.36066
H	-2.61491	1.32095	2.44983

$[Fe(phen)_3]^{2+}$ – Ground state (B3LYP)

Fe	0.05452	-0.04069	-0.02261
N	1.20196	-1.35695	-1.00565
N	-1.25808	-0.08179	-1.53292
N	0.76585	1.53025	-1.03548
N	1.33948	0.15262	1.49871
N	-1.05552	1.18102	1.10906
N	-0.66286	-1.70574	0.82655
C	2.16632	3.39009	-1.57301
C	-1.44146	2.11494	-4.51954
C	1.78375	2.32847	-0.74306
C	-0.40381	2.95251	-4.25724
C	1.47423	3.63071	-2.73789
C	2.81269	0.60450	3.83090
C	3.30188	-0.18947	2.81891
C	2.53606	-0.39515	1.66440
C	-3.07623	-0.83126	-2.89133
C	0.93364	2.04133	4.66739
C	2.58277	-3.49030	-2.17360
C	-0.29460	2.58485	4.45981
C	-2.84190	0.15181	-3.82534
C	1.60222	-3.74878	-1.19795
C	-1.02231	2.32913	3.25331
C	-0.39337	-4.12275	0.78506
C	1.54427	1.19639	3.68577
C	-1.78181	1.05430	-3.61937
C	2.84060	-2.18794	-2.53509
C	-2.25336	1.69675	0.86751
C	-1.98712	-3.09241	2.25380
C	0.38644	2.80314	-3.07250
C	0.07043	1.76546	-2.17730
C	2.12994	-1.14375	-1.92934
C	-0.06402	-2.82680	0.34854
C	1.25015	-5.05925	-0.74094
C	-2.26224	-0.91945	-1.75459
C	-2.29586	2.85712	2.97100
C	-1.01731	0.88980	-2.45052
C	-1.39198	-4.23491	1.77028
C	0.93709	-2.63924	-0.64565
C	0.29458	-5.23841	0.20853
C	-2.90306	2.54155	1.77682
C	-1.59675	-1.84157	1.75860
C	0.84600	0.93316	2.49377
C	-0.44228	1.49807	2.27832
H	3.00660	4.00581	-1.28452
H	-2.03354	2.23510	-5.41774
H	2.32363	2.12009	0.16912
H	-0.15381	3.75164	-4.94333
H	1.75466	4.44276	-3.39667
H	3.38800	0.77752	4.73147
H	4.27178	-0.66039	2.89535
H	2.90833	-1.01271	0.86013
H	-3.88081	-1.54203	-3.01693
H	1.47608	2.23938	5.58303
H	3.12132	-4.31305	-2.62635
H	-0.74781	3.22346	5.20722
H	-3.45761	0.23955	-4.71142
H	3.58621	-1.95127	-3.28102
H	-2.72243	1.43052	-0.06836
H	-2.75652	-3.13951	3.01151
H	2.32873	-0.11725	-2.20035
H	1.76540	-5.90836	-1.17137
H	-2.43727	-1.68407	-1.01191
H	-2.78265	3.50253	3.69102
H	-1.68005	-5.21259	2.13517
H	0.03462	-6.23303	0.54770
H	-3.87954	2.93085	1.52514
H	-2.06177	-0.93822	2.12512

[Fe(phen)$_3$]$^{2+}$ – ^{3}MC state (B3LYP)

Fe	0.02542	-0.09587	-0.11015	H	2.93542	3.95068	-1.32264
N	1.32054	-1.50086	-1.09803	H	-2.14870	2.41088	-5.48609
N	-1.43080	-0.07978	-1.70723	H	2.23242	2.03234	0.07850
N	0.66333	1.49678	-1.13389	H	-0.23456	3.86746	-4.95523
N	1.38105	0.16938	1.64223	H	1.67097	4.46417	-3.41310
N	-1.07358	1.26216	1.23138	H	3.37585	0.82063	4.88803
N	-0.62248	-1.79125	0.72422	H	4.27720	-0.67101	3.10120
C	2.08573	3.35492	-1.62471	H	2.93439	-1.02878	1.04097
C	-1.55679	2.24385	-4.59528	H	-4.09275	-1.40352	-3.24764
C	1.69397	2.27464	-0.82618	H	1.51902	2.31102	5.68531
C	-0.50154	3.04652	-4.30224	H	3.31643	-4.50177	-2.49868
C	1.38738	3.63658	-2.77534	H	-0.68388	3.34282	5.29016
C	2.81679	0.63497	3.97950	H	-3.61135	0.43856	-4.85763
C	3.31650	-0.18661	2.99645	H	3.79661	-2.17266	-3.24914
C	2.56245	-0.39365	1.83441	H	-2.74070	1.54298	0.06897
C	-3.26761	-0.72366	-3.08855	H	-2.76811	-3.18953	2.88295
C	0.97089	2.11404	4.77273	H	2.46935	-0.29662	-2.30125
C	2.75634	-3.66227	-2.10649	H	1.92806	-6.04224	-1.03781
C	-0.24214	2.68191	4.55525	H	-2.64072	-1.67284	-1.24595
C	-2.99979	0.29367	-3.97600	H	-2.68524	3.64956	3.80865
C	1.74070	-3.88767	-1.15800	H	-1.62958	-5.27052	2.10361
C	-0.97221	2.42074	3.35279	H	0.13451	-6.31378	0.62857
C	-0.31922	-4.20125	0.76235	H	-3.84226	3.09760	1.66632
C	1.56007	1.24330	3.80242	H	-2.06468	-1.00343	1.95633
C	-1.91556	1.15854	-3.73437				
C	3.02340	-2.37767	-2.52217				
C	-2.25670	1.80787	1.00014				
C	-1.97559	-3.15395	2.14897				
C	0.28664	2.83336	-3.12623				
C	-0.04260	1.76886	-2.26566				
C	2.27823	-1.31593	-1.99260				
C	0.00935	-2.91622	0.29047				
C	1.38772	-5.18280	-0.66215				
C	-2.45375	-0.88045	-1.95877				
C	-2.23366	2.98379	3.08387				
C	-1.15516	0.92543	-2.57208				
C	-1.34710	-4.29899	1.71853				
C	1.04610	-2.75965	-0.68039				
C	0.39917	-5.33220	0.25681				
C	-2.87399	2.68051	1.90512				
C	-1.58438	-1.91445	1.63050				
C	0.86861	0.96961	2.60312				
C	-0.42295	1.55985	2.37899				

$[Fe(phen)_3]^{2+}$ – ^{5}MC state (B3LYP)

Fe	0.03747	-0.03597	-0.02162
N	1.28789	-1.52929	-1.00673
N	-1.38076	0.02354	-1.69587
N	0.78699	1.60476	-1.24329
N	1.39687	0.24058	1.68284
N	-1.11236	1.22881	1.33188
N	-0.71580	-1.91269	0.79589
C	2.21635	3.42227	-1.84123
C	-1.50373	2.22470	-4.68124
C	1.82934	2.38078	-0.98980
C	-0.43116	3.02189	-4.44729
C	1.49163	3.65159	-2.98739
C	2.87275	0.78225	3.98023
C	3.38611	-0.01301	2.98286
C	2.61036	-0.26211	1.84369
C	-3.25590	-0.62896	-3.02625
C	0.97607	2.16518	4.82677
C	2.72272	-3.66595	-2.06966
C	-0.26769	2.67491	4.64180
C	-2.97378	0.34331	-3.95673
C	1.69014	-3.91238	-1.14592
C	-1.01294	2.38280	3.45581
C	-0.39735	-4.31121	0.71861
C	1.58165	1.32557	3.83908
C	-1.86317	1.18698	-3.76392
C	3.00783	-2.37193	-2.43770
C	-2.32277	1.72509	1.12708
C	-2.08631	-3.34599	2.13092
C	0.37463	2.84555	-3.27834
C	0.05340	1.82454	-2.36017
C	2.26228	-1.32650	-1.88101
C	-0.06680	-3.00012	0.31463
C	1.33069	-5.22819	-0.71487
C	-2.42795	-0.75814	-1.90380
C	-2.30301	2.89196	3.21476
C	-1.08768	0.98452	-2.60354
C	-1.44075	-4.46141	1.65086
C	0.99174	-2.79777	-0.63461
C	0.32772	-5.41981	0.17807
C	-2.95585	2.56793	2.04823
C	-1.68989	-2.08275	1.67711
C	0.87353	1.01881	2.65866
C	-0.44873	1.54948	2.46809
H	3.07705	4.02556	-1.58925
H	-2.11285	2.36030	-5.56585
H	2.38508	2.16889	-0.08555
H	-0.16417	3.80924	-5.14051
H	1.76600	4.44482	-3.67158
H	3.44648	0.99692	4.87305
H	4.37302	-0.44723	3.05997
H	2.98979	-0.88319	1.04276
H	-4.10088	-1.29268	-3.14440
H	1.53746	2.38707	5.72542
H	3.27941	-4.49903	-2.48000
H	-0.72185	3.31306	5.38909
H	-3.59236	0.47173	-4.83603
H	3.79320	-2.14707	-3.14556
H	-2.81288	1.44307	0.20427
H	-2.89007	-3.42541	2.84926
H	2.46670	-0.29926	-2.15232
H	1.87941	-6.06829	-1.12109
H	-2.62606	-1.51515	-1.15625
H	-2.76662	3.53538	3.95206
H	-1.72100	-5.45422	1.97982
H	0.05662	-6.41675	0.50143
H	-3.94547	2.94526	1.83255
H	-2.17935	-1.18857	2.04001

$Fe(phen)_2(CN)_2$ – Ground state (B3LYP)

Fe	-0.04131	0.00551	-0.01372
N	2.12536	2.20780	-0.19901
N	2.00263	-2.01205	1.14271
N	0.27709	-0.69698	-1.86366
N	-1.47063	-1.44457	-0.03064
N	-0.62113	0.69118	1.77984
N	-1.45997	1.31966	-0.64364
C	1.31614	1.37215	-0.13550
C	1.24116	-1.24331	0.71080
C	1.17379	-0.30492	-2.75968
C	1.26932	-0.88329	-4.03187
C	0.41196	-1.89906	-4.38787
C	-0.54594	-2.34127	-3.45741
C	-1.48625	-3.39029	-3.71458
C	-2.38748	-3.77237	-2.77243
C	-2.42832	-3.13860	-1.48892
C	-3.33271	-3.48550	-0.46861
C	-3.28122	-2.80940	0.72892
C	-2.33408	-1.79278	0.91200
C	-1.51373	-2.10415	-1.21484
C	-0.56882	-1.70388	-2.20249
C	-0.16831	0.36369	2.98343
C	-0.69642	0.90966	4.16060
C	-1.72378	1.82260	4.09056
C	-2.22253	2.19374	2.82864
C	-3.28926	3.12925	2.63362
C	-3.72652	3.44554	1.38663
C	-3.13428	2.85551	0.22414
C	-3.53464	3.14139	-1.09391
C	-2.89110	2.51782	-2.13830
C	-1.85533	1.61159	-1.87413
C	-2.08552	1.93145	0.39257
C	-1.62905	1.59785	1.69983
H	1.83299	0.49699	-2.46074
H	2.02031	-0.51839	-4.71864
H	0.46459	-2.35998	-5.36601
H	-1.46165	-3.87482	-4.68236
H	-3.09418	-4.56711	-2.97520
H	-4.05390	-4.27568	-0.63522
H	-3.95929	-3.04805	1.53640
H	-2.28096	-1.25610	1.84832
H	0.63335	-0.35970	3.01437
H	-0.28655	0.60061	5.11206
H	-2.15100	2.25533	4.98634
H	-3.74170	3.58101	3.50729
H	-4.53363	4.15377	1.24811
H	-4.33806	3.84524	-1.27118
H	-3.16876	2.71373	-3.16459
H	-1.34004	1.11767	-2.68526

$Fe(phen)_2(CN)_2$ – ^{3}MC state (B3LYP)

Fe	0.06186	-0.04818	0.00117
N	2.22627	2.16476	-0.10387
N	2.09778	-2.15573	1.25338
N	0.29329	-0.72595	-2.10047
N	-1.35276	-1.50585	-0.11495
N	-0.71237	0.74713	1.99175
N	-1.44606	1.37110	-0.53552
C	1.41710	1.32907	-0.04922
C	1.36518	-1.37536	0.80016
C	1.14002	-0.34142	-3.03983
C	1.18716	-0.94522	-4.30401
C	0.31947	-1.97528	-4.58716
C	-0.58925	-2.40792	-3.60308
C	-1.52843	-3.46795	-3.80557
C	-2.38057	-3.84040	-2.81698
C	-2.36098	-3.19405	-1.53937
C	-3.21697	-3.55581	-0.48357
C	-3.12104	-2.88919	0.71520
C	-2.17167	-1.87149	0.86177
C	-1.44271	-2.14883	-1.30889
C	-0.55439	-1.74552	-2.35820
C	-0.31537	0.44180	3.21424
C	-0.87831	1.02359	4.35916
C	-1.88783	1.94688	4.21240
C	-2.32692	2.29257	2.92067
C	-3.37116	3.23879	2.67334
C	-3.75365	3.53693	1.40603
C	-3.12218	2.92078	0.27879
C	-3.48014	3.21176	-1.04985
C	-2.81705	2.58327	-2.07732
C	-1.80219	1.66750	-1.77618
C	-2.08900	1.98183	0.48849
C	-1.69179	1.65944	1.83052
H	1.80046	0.47856	-2.78706
H	1.89793	-0.59271	-5.03847
H	0.32543	-2.45909	-5.55596
H	-1.54462	-3.96531	-4.76707
H	-3.09174	-4.64166	-2.97305
H	-3.93585	-4.35265	-0.62650
H	-3.76038	-3.13845	1.55048
H	-2.07118	-1.34362	1.79851
H	0.47290	-0.29699	3.29467
H	-0.51746	0.73725	5.33740
H	-2.35006	2.41126	5.07457
H	-3.84949	3.71199	3.52165
H	-4.54424	4.25307	1.22037
H	-4.26929	3.92660	-1.24659
H	-3.06151	2.78245	-3.11135
H	-1.26563	1.16472	-2.56785

Fe(phen)$_2$(CN)$_2$ – ^{5}MC state (B3LYP)

Fe	0.36127	0.03230	0.07430
N	2.52696	2.48077	-0.44723
N	2.30841	-2.19836	1.55575
N	0.41973	-0.81709	-1.96091
N	-1.34170	-1.51316	-0.00983
N	-0.55423	0.81561	1.92267
N	-1.35937	1.38906	-0.60987
C	1.78185	1.60094	-0.29980
C	1.63305	-1.38939	1.06514
C	1.30600	-0.49476	-2.89038
C	1.28451	-1.04298	-4.17837
C	0.29748	-1.94368	-4.50370
C	-0.65779	-2.30538	-3.53604
C	-1.71601	-3.23209	-3.79814
C	-2.61124	-3.55942	-2.83297
C	-2.51858	-2.99367	-1.52226
C	-3.41300	-3.31672	-0.48515
C	-3.24745	-2.74163	0.75274
C	-2.19086	-1.84194	0.94819
C	-1.48784	-2.07277	-1.23059
C	-0.54804	-1.71573	-2.25789
C	-0.13475	0.54792	3.14983
C	-0.77385	1.04327	4.29277
C	-1.88980	1.83279	4.14343
C	-2.35717	2.13570	2.85128
C	-3.50987	2.94931	2.61354
C	-3.92092	3.22373	1.35020
C	-3.21198	2.71066	0.21849
C	-3.59251	2.98054	-1.10911
C	-2.85219	2.45787	-2.14326
C	-1.73640	1.66312	-1.84656
C	-2.07300	1.89934	0.41751
C	-1.64208	1.60360	1.75640
H	2.05542	0.23305	-2.60745
H	2.03524	-0.74580	-4.89712
H	0.24363	-2.37969	-5.49343
H	-1.78359	-3.66686	-4.78733
H	-3.41051	-4.26157	-3.03415
H	-4.21768	-4.01637	-0.67412
H	-3.91331	-2.96989	1.57337
H	-2.03848	-1.37984	1.91491
H	0.73445	-0.09165	3.23184
H	-0.38761	0.79273	5.27084
H	-2.41547	2.22548	5.00489
H	-4.04505	3.34297	3.46837
H	-4.79258	3.84132	1.17352
H	-4.46127	3.59844	-1.29926
H	-3.11348	2.64747	-3.17508
H	-1.13775	1.24093	-2.64324

$[Fe(phen)(CN)_4]^{2-}$ – Ground state (B3LYP)

Fe	-0.04062	-0.01603	-0.00520
N	1.76365	0.29271	0.88005
N	0.85733	1.06428	-1.47840
N	1.08368	-2.60661	-1.33285
N	-1.06164	2.63766	1.28040
N	-2.67783	-0.35733	-1.57878
N	-1.12767	-1.63635	2.39082
C	4.74449	2.10699	-0.39089
C	4.27932	2.50077	-1.60504
C	2.18961	-0.10741	2.07015
C	3.47447	0.18065	2.54955
C	4.34669	0.90753	1.77148
C	3.92443	1.34685	0.50397
C	2.61552	1.01024	0.10585
C	2.12712	1.42536	-1.16687
C	2.95227	2.17172	-2.03123
C	2.41177	2.54879	-3.27363
C	1.12184	2.17796	-3.57934
C	0.37424	1.43475	-2.65618
C	0.64998	-1.65092	-0.82341
C	-0.69884	1.63817	0.80026
C	-1.68532	-0.22623	-0.97807
C	-0.72969	-1.02470	1.47937
H	5.74881	2.36108	-0.07583
H	4.90608	3.07445	-2.27623
H	1.48792	-0.67697	2.66265
H	3.76139	-0.17503	3.52962
H	5.34504	1.14335	2.11817
H	3.01066	3.12245	-3.96966
H	0.67052	2.44914	-4.52390
H	-0.63955	1.13040	-2.87451

[Fe(phen)(CN)$_4$]$^{2-}$ – ^{3}MC state (B3LYP)

Fe	-0.24554	-0.11482	0.03838
N	1.82606	0.32905	0.90419
N	0.89522	1.12158	-1.50527
N	0.97063	-2.64605	-1.32303
N	-1.24111	2.54113	1.34131
N	-2.89696	-0.29784	-1.84240
N	-1.10803	-1.83577	2.66928
C	4.79992	2.11382	-0.41130
C	4.33722	2.49920	-1.62731
C	2.26008	-0.05667	2.09079
C	3.54874	0.23347	2.56087
C	4.40780	0.94786	1.75863
C	3.97246	1.37117	0.48972
C	2.65431	1.03436	0.10758
C	2.16110	1.45060	-1.17714
C	3.00557	2.18074	-2.04358
C	2.48044	2.56164	-3.29178
C	1.18706	2.21882	-3.61040
C	0.42534	1.49521	-2.68212
C	0.49422	-1.71577	-0.80574
C	-0.89445	1.53844	0.85703
C	-1.93481	-0.21051	-1.19234
C	-0.76893	-1.21405	1.74484
H	5.80726	2.36005	-0.09919
H	4.96767	3.05848	-2.30732
H	1.56110	-0.62058	2.69565
H	3.84849	-0.10779	3.54232
H	5.41125	1.18892	2.08731
H	3.09905	3.11933	-3.98398
H	0.74978	2.49512	-4.56015
H	-0.59528	1.20858	-2.90254

$[Fe(phen)(CN)_4]^{2-}$ – ^{5}MC state (B3LYP)

Fe	-0.25905	-0.12098	0.03991
N	1.81750	0.32722	0.92002
N	0.88076	1.12339	-1.51538
N	1.24588	-2.78178	-1.48488
N	-1.18247	2.85478	1.43007
N	-3.01886	-0.36230	-1.86026
N	-1.18284	-1.89214	2.74401
C	4.79210	2.10166	-0.41165
C	4.32926	2.48975	-1.62600
C	2.25787	-0.05594	2.10580
C	3.54959	0.23120	2.56768
C	4.40340	0.94083	1.75662
C	3.96201	1.36255	0.48895
C	2.64095	1.02930	0.11122
C	2.14792	1.44611	-1.17525
C	2.99641	2.17538	-2.03959
C	2.47700	2.56292	-3.28847
C	1.18436	2.22793	-3.61581
C	0.41740	1.50317	-2.69350
C	0.70754	-1.90103	-0.94493
C	-0.91343	1.82897	0.94809
C	-2.05234	-0.26408	-1.22040
C	-0.85359	-1.27791	1.81257
H	5.80058	2.34292	-0.09953
H	4.95958	3.04748	-2.30731
H	1.56174	-0.61629	2.71741
H	3.85513	-0.10800	3.54793
H	5.40985	1.18070	2.07684
H	3.10197	3.12071	-3.97483
H	0.75252	2.51036	-4.56611
H	-0.60309	1.22084	-2.92087

Modified complex 1 – Ground state (B3LYP)

Fe	-0.08648	0.12383	0.05871
N	-2.60449	0.89218	1.40840
N	-0.05077	2.19997	2.16500
N	-2.23235	-1.32467	1.41137
N	0.92941	2.85347	0.25080
N	0.96810	-0.95910	1.40193
N	-0.28702	-1.72902	-0.79957
N	1.57831	0.30771	-1.12274
N	-0.94804	0.97331	-1.56183
C	-1.79482	-0.11626	1.06163
C	0.28802	1.88840	0.90803
C	-3.47027	-1.62782	2.11808
C	1.26306	4.17743	0.76023
C	1.58508	-0.56224	2.50960
C	2.36524	-1.41809	3.29632
C	2.51986	-2.73365	2.92767
C	1.88321	-3.19080	1.76048
C	1.98063	-4.53677	1.28299
C	1.34260	-4.92772	0.14968
C	0.54947	-4.00546	-0.60378
C	-0.15301	-4.35024	-1.77263
C	-0.90283	-3.38884	-2.40983
C	-0.94810	-2.08867	-1.89151
C	0.44799	-2.67278	-0.15840
C	1.11487	-2.26131	1.03194
C	2.84047	-0.00120	-0.85832
C	3.85890	0.09764	-1.81442
C	3.55981	0.53154	-3.08539
C	2.23234	0.87873	-3.39490
C	1.81192	1.35636	-4.67651
C	0.51643	1.69341	-4.90538
C	-0.47019	1.58662	-3.87359
C	-1.82295	1.92446	-4.05539
C	-2.69091	1.77892	-2.99909
C	-2.21872	1.30034	-1.77090
C	-0.08267	1.11445	-2.60398
C	1.27522	0.75016	-2.36934
H	-4.34204	-1.30270	1.54455
H	-3.49312	-1.14359	3.09749
H	-3.52924	-2.70352	2.26042
H	0.36335	4.72687	1.04826
H	1.77039	4.72912	-0.02668
H	1.92767	4.11271	1.62562
H	1.47025	0.47395	2.78553
H	2.83870	-1.02448	4.18492
H	3.12100	-3.41517	3.51572
H	2.57649	-5.23914	1.85179
H	1.41843	-5.94823	-0.20349
H	-0.10166	-5.36301	-2.15167
H	-1.46388	-3.61693	-3.30525
H	-1.54200	-1.33022	-2.37933
H	3.06402	-0.33489	0.14406
H	4.86874	-0.16933	-1.53597
H	4.32785	0.61550	-3.84365
H	2.55279	1.44488	-5.46077
H	0.20306	2.05462	-5.87662
H	-2.16270	2.28821	-5.01672
H	-3.73965	2.02254	-3.09630
H	-2.89723	1.16968	-0.94302
H	0.14957	3.08870	2.60072
H	-1.66470	-2.11283	1.15772
H	1.22285	2.65584	-0.68861
H	-3.48828	0.77069	1.88166
H	-0.58255	1.54836	2.71393
H	-2.33587	1.83924	1.20910

Modified complex 1 – ^{3}MC state (B3LYP)

Fe	-0.13380	0.18926	0.12788
N	-2.77946	0.92617	1.14512
N	0.22122	2.09331	2.33148
N	-2.11544	-1.12250	1.83822
N	0.45237	3.05115	0.29452
N	1.18149	-1.17758	1.52787
N	-0.34491	-1.74039	-0.65987
N	1.52936	0.46813	-1.10043
N	-1.01766	1.05339	-1.87139
C	-1.84090	-0.02303	1.14797
C	0.20776	1.95741	1.00408
C	-3.33851	-1.39277	2.58406
C	0.72308	4.38206	0.82483
C	1.89683	-0.89693	2.60478
C	2.69595	-1.84350	3.25942
C	2.75037	-3.12450	2.76314
C	1.99931	-3.45604	1.62075
C	1.99450	-4.76772	1.05332
C	1.23475	-5.04879	-0.03391
C	0.41861	-4.04433	-0.64331
C	-0.40599	-4.31715	-1.74862
C	-1.18812	-3.31212	-2.26348
C	-1.12946	-2.03955	-1.68787
C	0.41640	-2.73060	-0.12266
C	1.21885	-2.43410	1.03291
C	2.78208	0.25969	-0.71518
C	3.87580	0.35398	-1.58058
C	3.66202	0.67590	-2.89898
C	2.35021	0.92566	-3.33854
C	2.05288	1.29108	-4.68945
C	0.78223	1.56190	-5.07739
C	-0.29619	1.49611	-4.14167
C	-1.62518	1.79957	-4.48892
C	-2.60717	1.73185	-3.52869
C	-2.25669	1.35171	-2.22657
C	-0.04061	1.12267	-2.80231
C	1.30424	0.81958	-2.39461
H	-4.20718	-1.40624	1.92129
H	-3.49838	-0.64063	3.36021
H	-3.24431	-2.36679	3.05621
H	-0.10691	4.73203	1.44337
H	0.84876	5.06543	-0.01046
H	1.63667	4.38732	1.42389
H	1.85800	0.12382	2.96282
H	3.25965	-1.55344	4.13520
H	3.36026	-3.88326	3.23741
H	2.60535	-5.53178	1.51719
H	1.22234	-6.04361	-0.46069
H	-0.42046	-5.31369	-2.17134
H	-1.84686	-3.48170	-3.10362
H	-1.73937	-1.24113	-2.07911
H	2.93210	0.00240	0.32123
H	4.86936	0.16796	-1.19751
H	4.48348	0.74967	-3.60024
H	2.87281	1.35276	-5.39369
H	0.56109	1.84309	-6.09927
H	-1.85739	2.08421	-5.50758
H	-3.63854	1.95876	-3.76042
H	-3.01571	1.27040	-1.45922
H	0.40258	2.96524	2.80892
H	-1.41794	-1.84628	1.83922
H	0.44384	2.95630	-0.70613
H	-3.66707	0.84638	1.62125
H	-0.05844	1.32247	2.91188
H	-2.58834	1.81246	0.71201

Modified complex 1 – ^{5}MC state (B3LYP)

Fe	-0.22914	0.26098	0.24554
N	-2.93417	0.98149	1.63430
N	-0.07259	2.52323	2.39945
N	-2.66398	-1.24727	1.36034
N	1.15559	2.99552	0.55947
N	1.04110	-1.05714	1.52436
N	-0.30604	-1.85418	-0.70073
N	1.56850	0.38926	-1.20587
N	-1.03201	1.02332	-1.69705
C	-2.17387	-0.02628	1.20655
C	0.34595	2.15677	1.18816
C	-3.96256	-1.59735	1.92560
C	1.65321	4.27054	1.06505
C	1.66660	-0.67140	2.62584
C	2.53227	-1.50770	3.34137
C	2.75939	-2.78355	2.88137
C	2.10786	-3.22395	1.71469
C	2.29279	-4.53498	1.17197
C	1.63931	-4.92364	0.04857
C	0.73973	-4.03609	-0.62183
C	0.02391	-4.40134	-1.77725
C	-0.83735	-3.49793	-2.35473
C	-0.97363	-2.22814	-1.77980
C	0.53850	-2.73279	-0.11598
C	1.23940	-2.31661	1.06917
C	2.83224	0.09647	-0.94643
C	3.83431	0.12415	-1.92446
C	3.49766	0.46901	-3.21241
C	2.16264	0.79309	-3.51723
C	1.73584	1.17517	-4.82783
C	0.44101	1.49696	-5.07189
C	-0.53551	1.46373	-4.02674
C	-1.88543	1.80180	-4.23477
C	-2.76550	1.74826	-3.17981
C	-2.29571	1.34779	-1.92286
C	-0.15092	1.08120	-2.72294
C	1.22229	0.73948	-2.46477
H	-4.77337	-1.14430	1.34967
H	-4.04051	-1.26424	2.96325
H	-4.07309	-2.67789	1.89555
H	0.82953	4.96001	1.26461
H	2.30333	4.71012	0.31351
H	2.22387	4.12890	1.98606
H	1.48633	0.34386	2.95419
H	3.01412	-1.13680	4.23524
H	3.43175	-3.45396	3.40181
H	2.96729	-5.21056	1.68270
H	1.78063	-5.91664	-0.35907
H	0.15625	-5.39175	-2.19470
H	-1.40649	-3.74786	-3.23923
H	-1.64588	-1.49930	-2.21317
H	3.06919	-0.17128	0.07480
H	4.85106	-0.12554	-1.65507
H	4.24480	0.49955	-3.99563
H	2.47310	1.20537	-5.62004
H	0.12026	1.79010	-6.06355
H	-2.21402	2.09914	-5.22280
H	-3.80932	2.00103	-3.30314
H	-2.97029	1.28229	-1.07932
H	0.19544	3.38121	2.86473
H	-2.09263	-2.00637	1.02915
H	1.47576	2.71372	-0.35148
H	-3.84949	0.87197	2.05213
H	-0.66380	1.90424	2.92785
H	-2.61593	1.92823	1.51428

$[Fe(phen)_2(CN^{tBu}Ar_3NC)]^{2+}$ – Ground state (B3LYP)

Fe	0.01998	-0.13313	-0.30388
N	2.02443	2.11831	-0.26227
N	2.02248	-1.79553	1.22673
N	0.76067	-1.15911	-1.86164
N	-1.31183	-1.65948	-0.29335
N	-0.92657	0.85380	1.16543
N	-1.24410	0.95252	-1.45747
C	1.27499	1.23769	-0.34803
C	1.21007	-1.12350	0.74294
C	1.82938	-0.89261	-2.59966
C	2.26422	-1.74755	-3.61943
C	1.57621	-2.91091	-3.87426
C	0.44545	-3.22575	-3.09721
C	-0.33410	-4.41530	-3.26004
C	-1.40453	-4.66750	-2.46176
C	-1.78790	-3.75304	-1.42909
C	-2.87005	-3.95986	-0.55342
C	-3.13823	-3.02178	0.41626
C	-2.33318	-1.87987	0.52001
C	-1.03954	-2.57496	-1.25654
C	0.07859	-2.30941	-2.09576
C	-0.72314	0.79471	2.47459
C	-1.44342	1.58248	3.38032
C	-2.39204	2.46332	2.91440
C	-2.62151	2.55645	1.52948
C	-3.56663	3.45047	0.93177
C	-3.72920	3.50054	-0.41642
C	-2.96094	2.66088	-1.28490
C	-3.06532	2.67084	-2.68810
C	-2.26538	1.83027	-3.42730
C	-1.36022	0.98191	-2.77624
C	-2.02962	1.77322	-0.71763
C	-1.85847	1.72159	0.69379
C	3.08605	-2.56185	1.66286
C	2.88732	-3.62824	2.56655
C	4.01890	-4.35540	2.91723
C	5.29737	-4.09036	2.41643
C	5.43414	-3.04969	1.50757
C	4.34635	-2.27192	1.11502
C	3.04632	3.04114	-0.17461
C	2.77942	4.40580	0.05830
C	3.88712	5.24476	0.09336
C	5.20170	4.80342	-0.09421
C	5.40893	3.44770	-0.32135
C	4.34695	2.54872	-0.35621
C	4.56787	-1.21628	0.09190
C	5.13517	-1.58115	-1.13301
C	5.43373	-0.61785	-2.08626
C	5.17687	0.72250	-1.82857
C	4.60063	1.10807	-0.61654
C	4.29373	0.13063	0.33323
C	6.59757	-4.89571	4.40064
C	6.47891	-4.95234	2.86566
C	7.80650	-4.47956	2.26134
C	6.23617	-6.40844	2.42371
C	0.57328	-4.43044	1.95826
C	1.50183	-4.00741	3.11618
C	0.88951	-2.83115	3.90091
C	1.58162	-5.19803	4.08402
C	1.35553	6.46588	0.50243
C	1.35724	4.94608	0.27760
C	0.74445	4.30172	1.53722
C	0.47219	4.67613	-0.95737
C	7.71629	5.15540	-0.24498
C	6.34791	5.81491	-0.03846
C	6.34514	6.50891	1.33766
C	6.14555	6.86958	-1.14415
H	2.36494	0.01898	-2.37995
H	3.14295	-1.47850	-4.18748
H	1.89485	-3.58853	-4.65608
H	-0.04723	-5.11441	-4.03490
H	-1.98721	-5.57085	-2.58833
H	-3.47357	-4.85343	-0.64851
H	-3.95874	-3.14975	1.10795
H	-2.52376	-1.14137	1.28482
H	0.03382	0.10828	2.82210
H	-1.23590	1.48761	4.43665
H	-2.95643	3.08747	3.59517
H	-4.14798	4.09099	1.58218
H	-4.44257	4.18193	-0.86198
H	-3.76785	3.33908	-3.16958
H	-2.31730	1.81222	-4.50671
H	-0.71914	0.32219	-3.34242
H	3.90604	-5.17460	3.60749
H	6.39750	-2.81155	1.08351
H	3.72711	6.29485	0.27348

H	6.40440	3.05596	-0.46535
H	5.33762	-2.62485	-1.33491
H	5.86454	-0.91267	-3.03448
H	5.41078	1.47477	-2.57084
H	3.86576	0.42663	1.28256
H	6.76450	-3.87024	4.73701
H	7.44161	-5.50588	4.72888
H	5.69886	-5.27269	4.88958
H	8.03648	-3.45184	2.54895
H	8.61355	-5.11827	2.62368
H	7.79821	-4.53850	1.17141
H	7.06779	-7.03765	2.74820
H	5.31835	-6.81154	2.85342
H	6.16213	-6.47329	1.33614
H	0.40948	-3.63867	1.23274
H	-0.39980	-4.71775	2.36119
H	0.99252	-5.29093	1.43356
H	0.72993	-1.95417	3.28159
H	-0.07803	-3.13089	4.30790
H	1.53736	-2.54794	4.73270
H	0.57680	-5.42041	4.44541
H	1.96592	-6.09501	3.59602
H	2.20751	-4.97836	4.95049
H	1.92717	6.74883	1.38768
H	0.32589	6.79391	0.65178
H	1.75676	7.00431	-0.35768
H	1.33878	4.55425	2.41735
H	-0.26755	4.68156	1.68701
H	0.68712	3.21901	1.47160
H	-0.52708	5.07720	-0.77704
H	0.88659	5.17477	-1.83570
H	0.36635	3.61983	-1.18624
H	7.92815	4.41440	0.52823
H	8.49369	5.91968	-0.19877
H	7.78570	4.66670	-1.21862
H	6.48067	5.77909	2.13854
H	7.16284	7.23088	1.38911
H	5.41158	7.04307	1.51702
H	6.95745	7.59926	-1.11214
H	6.14547	6.39957	-2.12978
H	5.20398	7.40585	-1.02110

[Fe(phen)$_2$(CN^{tBu}Ar$_3$NC)]$^{2+}$ – ^{3}MC state (B3LYP)

Fe	-0.03559	-0.14525	-0.33519
N	1.99706	2.09181	-0.18480
N	2.07592	-1.86032	1.22740
N	0.79271	-1.26035	-2.07334
N	-1.32391	-1.70353	-0.44949
N	-1.19319	0.97774	1.28832
N	-1.36204	1.07514	-1.41736
C	1.22799	1.22989	-0.27668
C	1.23512	-1.20660	0.77952
C	1.86710	-1.03562	-2.81043
C	2.33005	-1.95719	-3.75883
C	1.64666	-3.13773	-3.93600
C	0.50482	-3.40561	-3.15599
C	-0.26315	-4.60742	-3.26455
C	-1.35036	-4.81237	-2.47797
C	-1.75493	-3.84101	-1.50766
C	-2.86276	-4.01734	-0.65835
C	-3.17261	-3.04061	0.25787
C	-2.37326	-1.89504	0.33741
C	-1.01341	-2.65032	-1.37493
C	0.11992	-2.42439	-2.22085
C	-1.06543	0.92995	2.60262
C	-1.75890	1.79813	3.45713
C	-2.60381	2.73786	2.91418
C	-2.75262	2.81266	1.51652
C	-3.60000	3.76405	0.86668
C	-3.70285	3.79526	-0.48612
C	-2.96104	2.88531	-1.30408
C	-3.02513	2.89119	-2.70938
C	-2.26809	1.99397	-3.42531
C	-1.44034	1.09865	-2.73922
C	-2.11091	1.94111	-0.69289
C	-2.01228	1.89880	0.73832
C	3.14820	-2.60829	1.66832
C	2.96086	-3.66226	2.58783
C	4.10029	-4.37846	2.93257
C	5.37054	-4.11270	2.40967
C	5.49444	-3.07722	1.49160
C	4.39959	-2.30709	1.10701
C	3.02243	3.01094	-0.11436
C	2.76034	4.37491	0.13015
C	3.86773	5.21436	0.14125
C	5.17756	4.77407	-0.07920
C	5.37969	3.41966	-0.31775
C	4.31805	2.51959	-0.33204
C	4.59365	-1.24602	0.08468
C	5.14507	-1.59617	-1.15101
C	5.40702	-0.62268	-2.10482
C	5.13234	0.71159	-1.83473
C	4.57314	1.08219	-0.60989
C	4.29948	0.09468	0.33894
C	6.70352	-4.88676	4.38326
C	6.56014	-4.96688	2.85132
C	7.87677	-4.50373	2.21668
C	6.30963	-6.42970	2.43586
C	0.63146	-4.47023	2.03724
C	1.58442	-4.02633	3.16781
C	0.99284	-2.83256	3.94436
C	1.68176	-5.19887	4.15601
C	1.34786	6.43086	0.62514
C	1.34278	4.91447	0.37821
C	0.74259	4.25421	1.63565
C	0.44300	4.66528	-0.85054
C	7.68881	5.12523	-0.27658
C	6.32425	5.78570	-0.04897
C	6.34628	6.48717	1.32308
C	6.10141	6.83377	-1.15703
H	2.38490	-0.09928	-2.64840
H	3.21534	-1.72833	-4.33482
H	1.97594	-3.86861	-4.66391
H	0.04263	-5.35019	-3.99018
H	-1.93219	-5.72115	-2.56334
H	-3.45442	-4.92071	-0.73437
H	-4.01605	-3.14201	0.92591
H	-2.58365	-1.12242	1.06223
H	-0.39170	0.18165	3.00115
H	-1.61979	1.71754	4.52607
H	-3.15268	3.42501	3.54561
H	-4.15834	4.45918	1.48045
H	-4.34586	4.51485	-0.97664
H	-3.66718	3.60443	-3.21057
H	-2.29335	1.97003	-4.50554
H	-0.82293	0.39181	-3.27579
H	4.00131	-5.18850	3.63550
H	6.45255	-2.83735	1.05696
H	3.71131	6.26386	0.32783

H	6.37161	3.02956	-0.48787
H	5.36238	-2.63516	-1.36161
H	5.82412	-0.90484	-3.06294
H	5.34115	1.47119	-2.57700
H	3.88481	0.37800	1.29806
H	6.87184	-3.85601	4.70218
H	7.55517	-5.48918	4.70582
H	5.81440	-5.26044	4.89186
H	8.11391	-3.47279	2.48637
H	8.68925	-5.13900	2.57264
H	7.84797	-4.57730	1.12798
H	7.14914	-7.05238	2.75221
H	5.40215	-6.82766	2.89131
H	6.21274	-6.51080	1.35127
H	0.44233	-3.69214	1.30236
H	-0.32885	-4.75740	2.46970
H	1.04282	-5.33679	1.51638
H	0.82496	-1.95998	3.31934
H	0.03191	-3.12230	4.37364
H	1.65749	-2.54364	4.76081
H	0.68413	-5.41248	4.54154
H	2.05482	-6.10531	3.67681
H	2.32512	-4.96417	5.00554
H	1.93379	6.69971	1.50551
H	0.32120	6.75842	0.79471
H	1.73661	6.98131	-0.23298
H	1.35674	4.47892	2.50973
H	-0.25905	4.64968	1.81082
H	0.66224	3.17446	1.54985
H	-0.55713	5.05360	-0.64874
H	0.84227	5.18656	-1.72267
H	0.34273	3.61374	-1.10223
H	7.91236	4.38530	0.49435
H	8.46709	5.88928	-0.24398
H	7.74269	4.63513	-1.25049
H	6.49747	5.76181	2.12519
H	7.16416	7.21001	1.35515
H	5.41591	7.02168	1.51695
H	6.91349	7.56378	-1.14367
H	6.08393	6.35782	-2.13965
H	5.16182	7.37035	-1.02048

$[Fe(phen)_2(CN^{tBu}Ar_3NC)]^{2+}$ – ^{5}MC state (B3LYP)

Fe	-0.15099	-0.06459	-0.46850	C	5.49444	3.45005	-0.13806
N	2.06460	2.31645	-0.49926	C	4.39647	2.62360	-0.34637
N	2.09925	-1.65494	1.26311	C	4.55438	-1.15356	-0.06157
N	0.63389	-1.40140	-1.99871	C	5.01487	-1.48919	-1.33745
N	-1.44535	-1.86164	-0.31683	C	5.25656	-0.50032	-2.28122
N	-1.25708	1.11134	0.97646	C	5.05399	0.83657	-1.96164
N	-1.64710	0.96830	-1.70545	C	4.59001	1.19053	-0.69447
C	1.28790	1.49729	-0.75593	C	4.33266	0.19055	0.24369
C	1.23964	-0.96704	0.90615	C	6.83560	-4.87519	4.01365
C	1.66961	-1.16176	-2.78803	C	6.52586	-5.00960	2.50980
C	2.21039	-2.13725	-3.63279	C	7.79305	-4.65927	1.72140
C	1.65254	-3.39402	-3.64523	C	6.14329	-6.46810	2.19193
C	0.55494	-3.67791	-2.80980	C	0.58917	-4.19394	2.16024
C	-0.07589	-4.96103	-2.75539	C	1.64907	-3.82016	3.21812
C	-1.12543	-5.18796	-1.92530	C	1.18505	-2.61165	4.05689
C	-1.62940	-4.15283	-1.07605	C	1.76109	-5.01447	4.17838
C	-2.70453	-4.34014	-0.18663	C	1.53565	6.58559	0.71822
C	-3.11987	-3.29448	0.60364	C	1.48047	5.10761	0.30374
C	-2.45827	-2.06275	0.50880	C	0.73635	4.34902	1.42129
C	-1.02971	-2.87713	-1.10731	C	0.68563	5.03457	-1.01659
C	0.07531	-2.63561	-1.99155	C	7.86525	5.01687	0.26112
C	-1.05405	1.15881	2.28477	C	6.51827	5.72236	0.45511
C	-1.76270	2.02612	3.12258	C	6.44589	6.24663	1.90271
C	-2.70214	2.86762	2.57309	C	6.44454	6.91080	-0.52310
C	-2.93767	2.83780	1.18591	H	2.09308	-0.16690	-2.74971
C	-3.89573	3.67914	0.53674	H	3.05808	-1.89057	-4.25548
C	-4.09098	3.60821	-0.80448	H	2.04709	-4.17261	-4.28596
C	-3.34420	2.69114	-1.60990	H	0.30583	-5.74759	-3.39366
C	-3.51096	2.57595	-3.00310	H	-1.60033	-6.16000	-1.88617
C	-2.75238	1.66745	-3.70356	H	-3.18875	-5.30707	-0.13360
C	-1.82432	0.87685	-3.01254	H	-3.94121	-3.40562	1.29754
C	-2.38875	1.85265	-1.00025	H	-2.75734	-1.22516	1.12577
C	-2.18261	1.92797	0.41811	H	-0.30274	0.49018	2.68499
C	3.16110	-2.46676	1.60604	H	-1.55952	2.02562	4.18401
C	2.99217	-3.51746	2.53375	H	-3.26313	3.55570	3.19273
C	4.11659	-4.29352	2.78498	H	-4.46244	4.37361	1.14339
C	5.35166	-4.09479	2.15933	H	-4.81751	4.24526	-1.29272
C	5.45705	-3.06161	1.23682	H	-4.23514	3.20416	-3.50634
C	4.37875	-2.22827	0.94873	H	-2.85733	1.55313	-4.77329
C	3.11197	3.17279	-0.22809	H	-1.20990	0.15594	-3.53716
C	2.88883	4.51893	0.12545	H	4.03350	-5.09893	3.49505
C	4.03014	5.28494	0.33075	H	6.38894	-2.86945	0.72780
C	5.33286	4.78921	0.20256	H	3.90810	6.31935	0.60580

H	6.47796	3.01628	-0.23595
H	5.17514	-2.52933	-1.58937
H	5.60204	-0.77266	-3.27019
H	5.24843	1.60893	-2.69473
H	3.98448	0.46734	1.23071
H	7.11495	-3.84841	4.25864
H	7.66804	-5.53000	4.27956
H	5.97797	-5.15137	4.62803
H	8.12777	-3.64085	1.92720
H	8.59487	-5.33979	2.01172
H	7.64009	-4.76097	0.64527
H	6.97736	-7.12984	2.43432
H	5.27607	-6.79291	2.76769
H	5.91142	-6.58310	1.13107
H	0.37919	-3.38760	1.46410
H	-0.34657	-4.45054	2.66008
H	0.91621	-5.06191	1.58472
H	1.01432	-1.71853	3.46169
H	0.24776	-2.85958	4.55882
H	1.92843	-2.37586	4.82075
H	0.78427	-5.18774	4.63159
H	2.05496	-5.92857	3.66015
H	2.47327	-4.82629	4.98351
H	2.05725	6.72260	1.66671
H	0.51543	6.95075	0.84277
H	2.02216	7.20307	-0.03858
H	1.28259	4.42661	2.36317
H	-0.25237	4.78851	1.56387
H	0.59939	3.29574	1.19487
H	-0.29374	5.49427	-0.87020
H	1.20525	5.58337	-1.80434
H	0.52305	4.01606	-1.35998
H	7.98709	4.18198	0.95374
H	8.67242	5.72687	0.44787
H	7.97993	4.63936	-0.75680
H	6.48490	5.42021	2.61532
H	7.29185	6.90888	2.09817
H	5.52829	6.80820	2.08095
H	7.28433	7.58600	-0.34656
H	6.49387	6.56391	-1.55732
H	5.52214	7.47835	-0.39680

trans-$[Fe(py)_2(CN)_4]^{2-}$ – Ground state (B3LYP)

Fe	-0.00923	0.00151	-0.00040
N	-0.01216	-0.01255	2.06034
N	-0.00578	0.01588	-2.06148
N	-3.13451	-0.00967	-0.00493
N	3.11590	0.00509	0.00375
N	-0.00564	3.12654	0.02146
N	-0.01037	-3.12367	-0.02135
C	1.94903	0.00323	0.00232
C	-1.96765	-0.00189	-0.00329
C	-0.00827	-1.95683	-0.01345
C	-0.00963	1.95970	0.01323
C	-0.82444	0.79464	2.76002
C	-0.85568	0.81788	4.14555
C	-0.01368	-0.02797	4.85497
C	0.82877	-0.86622	4.13714
C	0.79897	-0.82776	2.75189
C	-0.81509	-0.79183	-2.76399
C	-0.84283	-0.81378	-4.14962
C	-0.00024	0.03391	-4.85613
C	0.83941	0.87248	-4.13538
C	0.80619	0.83265	-2.75025
H	-1.46633	1.43870	2.17956
H	-1.53334	1.49247	4.65118
H	-0.01408	-0.03377	5.93731
H	1.50581	-1.54645	4.63601
H	1.44128	-1.46561	2.16505
H	-1.45756	-1.43739	-2.18587
H	-1.51829	-1.48882	-4.65757
H	0.00198	0.04083	-5.93846
H	1.51687	1.55400	-4.63191
H	1.44632	1.47061	-2.16116

***trans*-[Fe(py)$_2$(CN)$_4$]$^{2-}$ – ^{3}MC state (B3LYP)**

Fe	-0.00748	0.00249	-0.00227
N	-0.01112	-0.01420	2.46797
N	-0.00523	0.01890	-2.47202
N	-3.15282	-0.18955	0.00620
N	3.13782	0.19410	0.01574
N	-0.10453	3.15146	0.01753
N	0.08926	-3.14652	-0.02337
C	1.97508	0.12591	0.00645
C	-1.99008	-0.12091	0.00047
C	0.05015	-1.98239	-0.01690
C	-0.06528	1.98735	0.00844
C	-0.74953	0.85802	3.15778
C	-0.78441	0.88641	4.54620
C	-0.01665	-0.03313	5.25058
C	0.75399	-0.94298	4.53680
C	0.72459	-0.89573	3.14877
C	-0.83049	-0.77079	-3.16204
C	-0.86458	-0.79679	-4.55051
C	-0.00072	0.03502	-5.25272
C	0.86079	0.85868	-4.53809
C	0.82223	0.81657	-3.15014
H	-1.33117	1.55905	2.57156
H	-1.39963	1.61408	5.05870
H	-0.01883	-0.04055	6.33321
H	1.36713	-1.67761	5.04179
H	1.30845	-1.58885	2.55540
H	-1.48604	-1.40213	-2.57486
H	-1.55278	-1.45443	-5.06478
H	0.00108	0.04129	-6.33538
H	1.55066	1.52222	-5.04245
H	1.47589	1.44094	-2.55352

***trans*-[Fe(py)$_2$(CN)$_4$]$^{2-}$ – ^{5}MC state (B3LYP)**

Fe	-0.00918	0.00201	-0.00047
N	-0.01370	-0.01553	2.28102
N	-0.00633	0.01901	-2.28207
N	-3.42044	-0.01993	-0.00297
N	3.40197	0.01942	0.00930
N	-0.02085	3.41319	0.02519
N	0.00624	-3.40911	-0.03325
C	2.23709	0.01318	0.00561
C	-2.25558	-0.01006	-0.00229
C	0.00206	-2.24428	-0.02130
C	-0.01876	2.24832	0.01632
C	-0.82231	0.79807	2.96683
C	-0.85455	0.82439	4.35345
C	-0.01343	-0.02883	5.05696
C	0.82731	-0.87550	4.34511
C	0.79479	-0.83590	2.95879
C	-0.82152	-0.78620	-2.97002
C	-0.85108	-0.81153	-4.35670
C	0.00047	0.03312	-5.05801
C	0.84838	0.87074	-4.34395
C	0.81204	0.83130	-2.95772
H	-1.45888	1.44437	2.37761
H	-1.52603	1.50094	4.86443
H	-0.01313	-0.03385	6.13948
H	1.49896	-1.55684	4.84947
H	1.43136	-1.47639	2.36326
H	-1.46614	-1.42600	-2.38247
H	-1.52859	-1.48070	-4.86944
H	0.00325	0.03852	-6.14052
H	1.52843	1.54501	-4.84655
H	1.45388	1.46495	-2.36054

cis-$[Fe(py)_2(CN)_4]^{2-}$ – Ground state (B3LYP)

Fe	0.03025	-0.01254	-0.08945
N	-2.04899	0.01470	-0.05090
N	-0.00471	2.06458	0.00303
N	0.11826	-0.14363	3.02902
N	-0.04279	0.09439	-3.20914
N	3.11861	0.03946	-0.17063
N	-0.00402	-3.10037	-0.21466
C	0.08476	-0.09298	1.86360
C	-0.01610	0.05472	-2.04310
C	1.95186	0.00916	-0.13529
C	0.01681	-1.93379	-0.16892
C	-2.74461	0.83807	-0.84969
C	-4.12928	0.89810	-0.85107
C	-4.84088	0.06889	0.00563
C	-4.12703	-0.78860	0.83254
C	-2.74211	-0.78375	0.77455
C	-0.76936	2.70479	0.90050
C	-0.83945	4.08650	0.98459
C	-0.08381	4.85450	0.10858
C	0.71422	4.19812	-0.81939
C	0.72449	2.81223	-0.83910
H	-2.16351	1.46353	-1.51044
H	-4.63245	1.58463	-1.51837
H	-5.92276	0.09000	0.02804
H	-4.62927	-1.45669	1.51905
H	-2.15689	-1.43492	1.40553
H	-1.33765	2.07955	1.57258
H	-1.47655	4.54380	1.72951
H	-0.11539	5.93566	0.14831
H	1.32448	4.74575	-1.52477
H	1.33023	2.27087	-1.54938

cis-$[Fe(py)_2(CN)_4]^{2-}$ – ^{3}MC state (B3LYP)

Fe	0.23449	-0.21539	-0.10209
N	-2.11368	0.00802	-0.05231
N	-0.00260	2.12872	0.00173
N	0.24862	-0.26725	3.01817
N	0.08915	-0.03685	-3.21368
N	3.45617	0.23021	-0.18475
N	-0.18986	-3.43986	-0.20967
C	0.25645	-0.25598	1.85196
C	0.15472	-0.11928	-2.05217
C	2.30099	0.08594	-0.15214
C	-0.05326	-2.28407	-0.16754
C	-2.76053	0.79976	-0.91211
C	-4.14352	0.91577	-0.93455
C	-4.89015	0.17738	-0.02463
C	-4.22113	-0.64900	0.86946
C	-2.83457	-0.70274	0.81857
C	-0.73221	2.71582	0.95441
C	-0.85276	4.09379	1.06876
C	-0.18737	4.90018	0.15357
C	0.57377	4.29337	-0.83768
C	0.63859	2.90681	-0.87395
H	-2.14289	1.35685	-1.60350
H	-4.61901	1.57086	-1.65226
H	-5.97060	0.24445	-0.01313
H	-4.75829	-1.24269	1.59694
H	-2.27480	-1.33363	1.49598
H	-1.23429	2.05285	1.64601
H	-1.45513	4.51942	1.86015
H	-0.26027	5.97873	0.21225
H	1.10942	4.87846	-1.57321
H	1.22086	2.39408	-1.62784

cis-$[Fe(py)_2(CN)_4]^{2-}$ – ^{5}MC state (B3LYP)

Fe	0.21496	-0.19233	-0.08963
N	-2.17264	-0.02488	-0.04245
N	0.04373	2.18978	0.00751
N	0.04197	-0.16289	3.28249
N	-0.05850	0.16808	-3.43799
N	3.56692	0.09689	-0.15429
N	-0.05583	-3.54193	-0.25545
C	0.07980	-0.14619	2.11874
C	0.01854	0.06553	-2.28059
C	2.41083	-0.03329	-0.12844
C	0.06823	-2.38641	-0.19559
C	-2.84499	0.80015	-0.84953
C	-4.23046	0.88082	-0.85793
C	-4.95123	0.06286	0.00370
C	-4.25544	-0.80012	0.84126
C	-2.86827	-0.80714	0.78701
C	-0.70640	2.81003	0.92229
C	-0.80274	4.19212	1.00427
C	-0.08250	4.96544	0.10193
C	0.70304	4.32378	-0.84767
C	0.73309	2.93598	-0.85972
H	-2.24613	1.41231	-1.51081
H	-4.72766	1.56939	-1.52787
H	-6.03310	0.09776	0.02212
H	-4.77261	-1.45627	1.52833
H	-2.28673	-1.45606	1.42852
H	-1.24324	2.17019	1.61021
H	-1.42810	4.64642	1.76100
H	-0.13290	6.04626	0.13791
H	1.28188	4.88302	-1.57036
H	1.32278	2.39543	-1.58825

References

(1) Paulus, B. C.; Nielsen, K. C.; Tichnell, C. R.; Carey, M. C.; McCusker, J. K. A Modular Approach to Light Capture and Synthetic Tuning of the Excited-State Properties of Fe(II)-Based Chromophores. *J. Am. Chem. Soc.* **2021**, *143* (21), 8086–8098. DOI: 10.1021/jacs.1c02451.

(2) Carey, M. C.; Adelman, S. L.; McCusker, J. K. Insights into the excited state dynamics of Fe(II) polypyridyl complexes from variable-temperature ultrafast spectroscopy. *Chem. Sci.* **2019**, *10* (1), 134–144. DOI: 10.1039/C8SC04025G.

(3) Alonso-Mori, R.; Caronna, C.; Chollet, M.; Curtis, R.; Damiani, D. S.; Defever, J.; Feng, Y.; Flath, D. L.; Glownia, J. M.; Lee, S.; Lemke, H. T.; Nelson, S.; Bong, E.; Sikorski, M.; Song, S.; Srinivasan, V.; Stefanescu, D.; Zhu, D.; Robert, A. The X-ray Correlation Spectroscopy instrument at the Linac Coherent Light Source. *J. Synchrotron Radiat.* **2015**, *22* (3), 508–513. DOI: 10.1107/S1600577515004397.

(4) Antolini, C.; Sosa Alfaro, V.; Reinhard, M.; Chatterjee, G.; Ribson, R.; Sokaras, D.; Gee, L.; Sato, T.; Kramer, P. L.; Raj, S. L.; Hayes, B.; Schleissner, P.; Garcia-Esparza, A. T.; Lim, J.; Babicz, J. T.; Follmer, A. H.; Nelson, S.; Chollet, M.; Alonso-Mori, R.; van Driel, T. B. The Liquid Jet Endstation for Hard X-ray Scattering and Spectroscopy at the Linac Coherent Light Source. *Molecules* **2024**, *29* (10), 2323. DOI: 10.3390/molecules29102323.

(5) Alonso-Mori, R.; Kern, J.; Sokaras, D.; Weng, T.-C.; Nordlund, D.; Tran, R.; Montanez, P.; Delor, J.; Yachandra, V. K.; Yano, J.; Bergmann, U. A multi-crystal wavelength dispersive x-ray spectrometer. *Rev. Sci. Instrum.* **2012**, *83* (7), 073114. DOI: 10.1063/1.4737630.

(6) Blaj, G.; Caragiulo, P.; Carini, G.; Carron, S.; Dragone, A.; Freytag, D.; Haller, G.; Hart, P.; Hasi, J.; Herbst, R.; Herrmann, S.; Kenney, C.; Markovic, B.; Nishimura, K.; Osier, S.; Pines, J.; Reese, B.; Segal, J.; Tomada, A.; Weaver, M. X-ray detectors at the Linac Coherent Light Source. *J. Synchrotron Radiat.* **2015**, *22* (3), 577–583. DOI: 10.1107/S1600577515005317.

(7) Carini, G. A.; Alonso-Mori, R.; Blaj, G.; Caragiulo, P.; Chollet, M.; Damiani, D.; Dragone, A.; Feng, Y.; Haller, G.; Hart, P.; Hasi, J.; Herbst, R.; Herrmann, S.; Kenney, C.; Lemke, H.; Manger, L.; Markovic, B.; Mehta, A.; Nelson, S.; Nishimura, K.; Osier, S.; Pines, J.; Reese, B.; Robert, A.; Segal, J.; Sikorski, M.; Song, S.; Thayer, J.; Tomada, A.; Weaver, M.; Zhu, D. ePix100 camera: Use and applications at LCLS. *AIP Conf. Proc.* **2016**, *1741* (1), 040008. DOI: 10.1063/1.4952880.

(8) van Driel, T. B.; Nelson, S.; Armenta, R.; Blaj, G.; Boo, S.; Boutet, S.; Doering, D.; Dragone, A.; Hart, P.; Haller, G.; Kenney, C.; Kwaitowski, M.; Manger, L.; McKelvey, M.; Nakahara, K.; Oriunno, M.; Sato, T.; Weaver, M. The ePix10k 2-megapixel hard X-ray detector at LCLS. *J. Synchrotron Radiat.* **2020**, *27* (3), 608–615. DOI: 10.1107/S1600577520004257.

(9) Harmand, M.; Coffee, R.; Bionta, M. R.; Chollet, M.; French, D.; Zhu, D.; Fritz, D. M.; Lemke, H. T.; Medvedev, N.; Ziaja, B.; Toleikis, S.; Cammarata, M. Achieving few-femtosecond time-sorting at hard X-ray free-electron lasers. *Nat. Photonics* **2013**, *7* (3), 215–218. DOI: 10.1038/nphoton.2013.11.

(10) Zhang, W.; Alonso-Mori, R.; Bergmann, U.; Bressler, C.; Chollet, M.; Galler, A.; Gawelda, W.; Hadt, R. G.; Hartsock, R. W.; Kroll, T.; Kjær, K. S.; Kubiček, K.; Lemke, H. T.; Liang, H. W.; Meyer, D.

A.; Nielsen, M. M.; Purser, C.; Robinson, J. S.; Solomon, E. I.; Sun, Z.; Sokaras, D.; van Driel, T. B.; Vankó, G.; Weng, T.-C.; Zhu, D.; Gaffney, K. J. Tracking excited-state charge and spin dynamics in iron coordination complexes. *Nature* **2014**, *509* (7500), 345–348. DOI: 10.1038/nature13252.

(11) Newville, M.; Otten, R.; Nelson, A.; Stensitzki, T.; Ingargiola, A.; Allan, D.; Fox, A.; Carter, F.; Michał; Osborn, R.; Pustakhod, D.; Weigand, S.; lneuhaus; Aristov, A.; Glenn; Mark; mgunyho; Deil, C.; Hansen, A. L. R.; Pasquevich, G.; Foks, L.; Zobrist, N.; Frost, O.; Stuermer; Jaskula, J.-C.; Caldwell, S.; Eendebak, P.; Pompili, M.; Nielsen, J. H.; Persaud, A. lmfit/lmfit-py: 1.3.2, 2024. DOI: 10.5281/zenodo.12785036.

(12) Lim, H.; Yang, X.; Larsen, C. B.; Ledbetter, K.; Zoric, M. R.; Raj, S. L.; Kumar, G.; Powers-Riggs, N.; Hoffmann, M. C.; Chollet, M.; Gee, L. B.; van Driel, T. B.; Alonso-Mori, R.; Kabanova, V.; Kahraman, A.; Johnson, P. J. M.; Cirelli, C.; Bacellar, C.; Gaffney, K. J.; Li, X.; Cordones, A. A. Excited State Covalency, Dynamics, and Photochemistry of Square Planar Ni-Thiolate Complexes Revealed by Ultrafast X-ray Absorption. *J. Am. Chem. Soc.* **2025**, *147* (9), 7496–7506. DOI: 10.1021/jacs.4c16212.

(13) Neese, F. The ORCA program system. *Wiley Interdiscip. Rev.: Comput. Mol. Sci.* **2012**, *2* (1), 73–78. DOI: 10.1002/wcms.81.

(14) Neese, F. Software update: The ORCA program system—Version 5.0. *Wiley Interdiscip. Rev.: Comput. Mol. Sci.* **2022**, *12* (5), e1606. DOI: 10.1002/wcms.1606.

(15) Weigend, F.; Ahlrichs, R. Balanced basis sets of split valence, triple zeta valence and quadruple zeta valence quality for H to Rn: Design and assessment of accuracy. *Phys. Chem. Chem. Phys.* **2005**, *7* (18), 3297–3305. DOI: 10.1039/B508541A.

(16) Weigend, F. Accurate Coulomb-fitting basis sets for H to Rn. *Phys. Chem. Chem. Phys.* **2006**, *8* (9), 1057–1065. DOI: 10.1039/B515623H.

(17) Grimme, S.; Antony, J.; Ehrlich, S.; Krieg, H. A consistent and accurate *ab initio* parametrization of density functional dispersion correction (DFT-D) for the 94 elements H-Pu. *J. Chem. Phys.* **2010**, *132* (15), 154104. DOI: 10.1063/1.3382344.

(18) Grimme, S.; Ehrlich, S.; Goerigk, L. Effect of the Damping Function in Dispersion Corrected Density Functional Theory. *J. Comput. Chem.* **2011**, *32* (7), 1456–1465. DOI: 10.1002/jcc.21759.

(19) Barone, V.; Cossi, M. Quantum Calculation of Molecular Energies and Energy Gradients in Solution by a Conductor Solvent Model. *J. Phys. Chem. A* **1998**, *102* (11), 1995–2001. DOI: 10.1021/jp9716997.

(20) Becke, A. D. Density-functional exchange-energy approximation with correct asymptotic behavior. *Phys. Rev. A* **1988**, *38* (6), 3098–3100. DOI: 10.1103/PhysRevA.38.3098.

(21) Perdew, J. P. Density-functional approximation for the correlation energy of the inhomogeneous electron gas. *Phys. Rev. B* **1986**, *33* (12), 8822–8824. DOI: 10.1103/PhysRevB.33.8822.

(22) Perdew, J. P.; Burke, K.; Ernzerhof, M. Generalized Gradient Approximation Made Simple. *Phys. Rev. Lett.* **1996**, *77* (18), 3865–3868. DOI: 10.1103/PhysRevLett.77.3865.

(23) Becke, A. D. Density-functional thermochemistry. III. The role of exact exchange. *J. Chem. Phys.* **1993**, *98* (7), 5648–5652. DOI: 10.1063/1.464913.

(24) Stephens, P. J.; Devlin, F. J.; Chabalowski, C. F.; Frisch, M. J. *Ab Initio* Calculation of Vibrational Absorption and Circular Dichroism Spectra Using Density Functional Force Fields. *J. Phys. Chem.* **1994**, *98* (45), 11623–11627. DOI: 10.1021/j100096a001.

(25) Adamo, C.; Barone, V. Toward reliable density functional methods without adjustable parameters: The PBE0 model. *J. Chem. Phys.* **1999**, *110* (13), 6158–6170. DOI: 10.1063/1.478522.

(26) Ernzerhof, M.; Scuseria, G. E. Assessment of the Perdew–Burke–Ernzerhof exchange-correlation functional. *J. Chem. Phys.* **1999**, *110* (11), 5029–5036. DOI: 10.1063/1.478401.

(27) Tao, J.; Perdew, J. P.; Staroverov, V. N.; Scuseria, G. E. Climbing the Density Functional Ladder: Nonempirical Meta–Generalized Gradient Approximation Designed for Molecules and Solids. *Phys. Rev. Lett.* **2003**, *91* (14), 146401. DOI: 10.1103/PhysRevLett.91.146401.

(28) Staroverov, V. N.; Scuseria, G. E.; Tao, J.; Perdew, J. P. Comparative assessment of a new nonempirical density functional: Molecules and hydrogen-bonded complexes. *J. Chem. Phys.* **2003**, *119* (23), 12129–12137. DOI: 10.1063/1.1626543.

(29) Meng, E. C.; Goddard, T. D.; Pettersen, E. F.; Couch, G. S.; Pearson, Z. J.; Morris, J. H.; Ferrin, T. E. UCSF ChimeraX: Tools for structure building and analysis. *Protein Sci.* **2023**, *32* (11), e4792. DOI: 10.1002/pro.4792.

(30) Schaefer, A. J.; Ingman, V. M.; Wheeler, S. E. SEQCROW: A ChimeraX bundle to facilitate quantum chemical applications to complex molecular systems. *J. Comput. Chem.* **2021**, *42* (24), 1750–1754. DOI: 10.1002/jcc.26700.

(31) Ingman, V. M.; Schaefer, A. J.; Andreola, L. R.; Wheeler, S. E. QChASM: Quantum chemistry automation and structure manipulation. *Wiley Interdiscip. Rev.: Comput. Mol. Sci.* **2021**, *11* (4), e1510. DOI: 10.1002/wcms.1510.

(32) Neese, F. Prediction and interpretation of the ^{57}Fe isomer shift in Mössbauer spectra by density functional theory. *Inorg. Chim. Acta* **2002**, *337*, 181–192. DOI: 10.1016/S0020-1693(02)01031-9.

(33) Panja, A. Mononuclear cobalt(III) and iron(II) complexes with diimine ligands: Synthesis, structure, DNA binding and cleavage activities, and oxidation of 2-aminophenol. *Polyhedron* **2012**, *43* (1), 22–30. DOI: 10.1016/j.poly.2012.05.041.

(34) Zhan, S.; Meng, Q.; You, X.; Wang, G.; Zheng, P.-J. The properties, crystal and molecular structure of *cis*-dicyano-bis(1,10-phenanthroline)iron(II) trihydrate. *Polyhedron* **1996**, *15* (15), 2655–2658. DOI: 10.1016/0277-5387(95)00540-4.

(35) Lescouëzec, R.; Lloret, F.; Julve, M.; Vaissermann, J.; Verdaguer, M.; Llusar, R.; Uriel, S. $[Fe(Phen)(CN)_4]^-$: A Versatile Building Block for the Design of Heterometallic Systems. Crystal Structures and Magnetic Properties of $PPh_4[Fe(Phen)(CN)_4]\cdot 2H_2O$ and $[\{Fe(Phen)(CN)_4\}_2M(H_2O)_2]\cdot 4H_2O$ [Phen = 1,10-Phenanthroline; M = Mn(II) and Zn(II)]. *Inorg. Chem.* **2001**, *40* (9), 2065–2072. DOI: 10.1021/ic001241a.

(36) Hanwell, M. D.; Curtis, D. E.; Lonie, D. C.; Vandermeersch, T.; Zurek, E.; Hutchison, G. R. Avogadro: an advanced semantic chemical editor, visualization, and analysis platform. *J. Cheminf.* **2012**, *4* (1), 17. DOI: 10.1186/1758-2946-4-17.

(37) Büldt, L. A.; Guo, X.; Vogel, R.; Prescimone, A.; Wenger, O. S. A Tris(diisocyanide)chromium(0) Complex Is a Luminescent Analog of $Fe(2,2'\text{-Bipyridine})_3^{2+}$. *J. Am. Chem. Soc.* **2017**, *139* (2), 985–992. DOI: 10.1021/jacs.6b11803.

(38) Lichtenberg, C.; Adelhardt, M.; Wörle, M.; Büttner, T.; Meyer, K.; Grützmacher, H. Mono- and Dinuclear Neutral and Cationic Iron(II) Compounds Supported by an Amidinato-diolefin Ligand: Characterization and Catalytic Application. *Organometallics* **2015**, *34* (12), 3079–3089. DOI: 10.1021/acs.organomet.5b00395.

(39) Reiher, M. Theoretical Study of the $Fe(phen)_2(NCS)_2$ Spin-Crossover Complex with Reparametrized Density Functionals. *Inorg. Chem.* **2002**, *41* (25), 6928–6935. DOI: 10.1021/ic0258911.

(40) Bowman, D. N.; Jakubikova, E. Low-Spin versus High-Spin Ground State in Pseudo-Octahedral Iron Complexes. *Inorg. Chem.* **2012**, *51* (11), 6011–6019. DOI: 10.1021/ic202344w.

(41) Kjær, K. S.; Kunnus, K.; Harlang, T. C. B.; Van Driel, T. B.; Ledbetter, K.; Hartsock, R. W.; Reinhard, M. E.; Koroidov, S.; Li, L.; Laursen, M. G.; Biasin, E.; Hansen, F. B.; Vester, P.; Christensen, M.; Haldrup, K.; Nielsen, M. M.; Chabera, P.; Liu, Y.; Tatsuno, H.; Timm, C.; Uhlig, J.; Sundstöm, V.; Németh, Z.; Szemes, D. S.; Bajnóczi, É.; Vankó, G.; Alonso-Mori, R.; Glownia, J. M.; Nelson, S.; Sikorski, M.; Sokaras, D.; Lemke, H. T.; Canton, S. E.; Wärnmark, K.; Persson, P.; Cordones, A. A.; Gaffney, K. J. Solvent control of charge transfer excited state relaxation pathways in $[Fe(2,2'\text{-bipyridine})(CN)_4]^{2-}$. *Phys. Chem. Chem. Phys.* **2018**, *20* (6), 4238–4249. DOI: 10.1039/C7CP07838B.

(42) Kjær, K. S.; Van Driel, T. B.; Harlang, T. C. B.; Kunnus, K.; Biasin, E.; Ledbetter, K.; Hartsock, R. W.; Reinhard, M. E.; Koroidov, S.; Li, L.; Laursen, M. G.; Hansen, F. B.; Vester, P.; Christensen, M.; Haldrup, K.; Nielsen, M. M.; Dohn, A. O.; Pápai, M. I.; Møller, K. B.; Chabera, P.; Liu, Y.; Tatsuno, H.; Timm, C.; Jarenmark, M.; Uhlig, J.; Sundstöm, V.; Wärnmark, K.; Persson, P.; Németh, Z.; Szemes, D. S.; Bajnóczi, É.; Vankó, G.; Alonso-Mori, R.; Glownia, J. M.; Nelson, S.; Sikorski, M.; Sokaras, D.; Canton, S. E.; Lemke, H. T.; Gaffney, K. J. Finding intersections between electronic excited state potential energy surfaces with simultaneous ultrafast X-ray scattering and spectroscopy. *Chem. Sci.* **2019**, *10* (22), 5749–5760. DOI: 10.1039/C8SC04023K.

(43) Kjær, K. S.; Zhang, W.; Alonso-Mori, R.; Bergmann, U.; Chollet, M.; Hadt, R. G.; Hartsock, R. W.; Harlang, T.; Kroll, T.; Kubiček, K.; Lemke, H. T.; Liang, H. W.; Liu, Y.; Nielsen, M. M.; Robinson, J. S.; Solomon, E. I.; Sokaras, D.; van Driel, T. B.; Weng, T.-C.; Zhu, D.; Persson, P.; Wärnmark, K.; Sundström, V.; Gaffney, K. J. Ligand manipulation of charge transfer excited state relaxation and spin crossover in $[Fe(2,2'\text{-bipyridine})_2(CN)_2]$. *Struct. Dyn.* **2017**, *4* (4), 044030. DOI: 10.1063/1.4985017.
